# Supplementary material for: The Novel Oral mTORC1/2 Inhibitor TAK-228 Reverses Trastuzumab Resistance in HER2-Positive Breast Cancer Models
Source: Cancers (Basel). 2021 Jun 3;13(11):2778. doi: 10.3390/cancers13112778 (PMC8199905; doi:10.3390/cancers13112778)
Supplement: Supplementary file 1 [file cancers-13-02778-s001.zip › Figure.S1-S5.docx]

The Novel Oral mTORC1/2 Inhibitor TAK-228 Reverses Trastuzumab Resistance in HER2-Positive Breast Cancer Models

**Marta Sanz-Álvarez, Ester Martín-Aparicio, Melani Luque, Sandra Zazo, Javier Martínez-Useros, Pilar Eroles, Ana Rovira, Joan Albanell, Juan Madoz-Gúrpide and Federico Rojo**


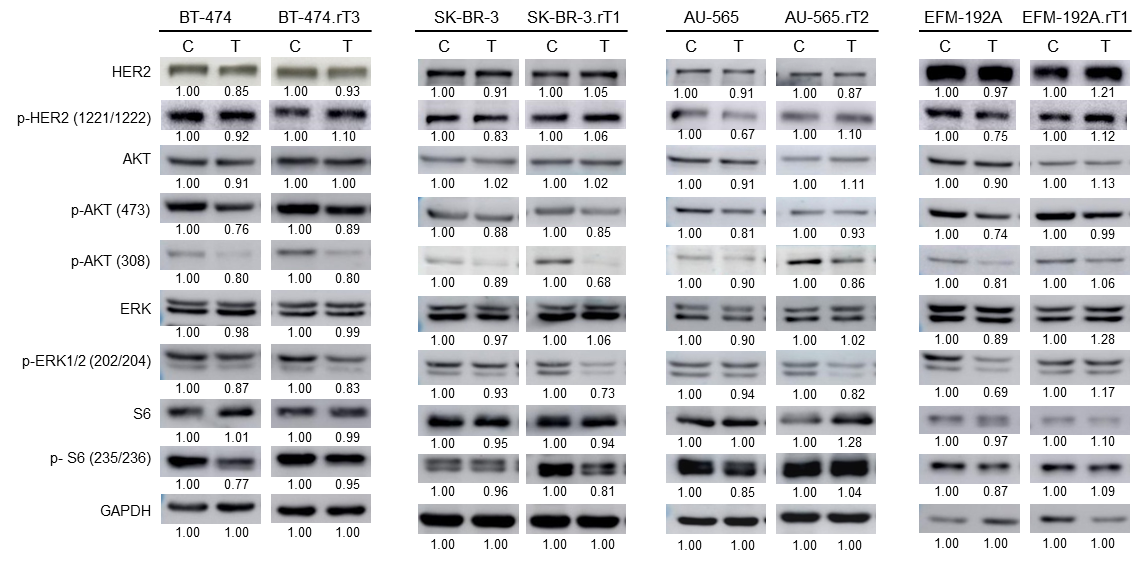


**Figure S1.** Immunoblotting analysis of trastuzumab-sensitive and -resistant cells. Cell lines were treated with 15 μg/mL trastuzumab for 24 h. Whole-cell protein extracts were analysed with the indicated antibodies. Images are representative of 3 independent experiments. C: control culture medium; T: trastuzumab 15 μg/mL.

**
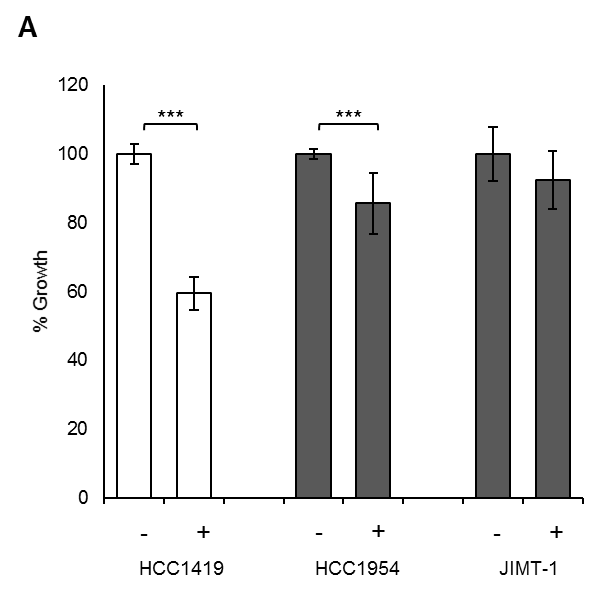
**

**
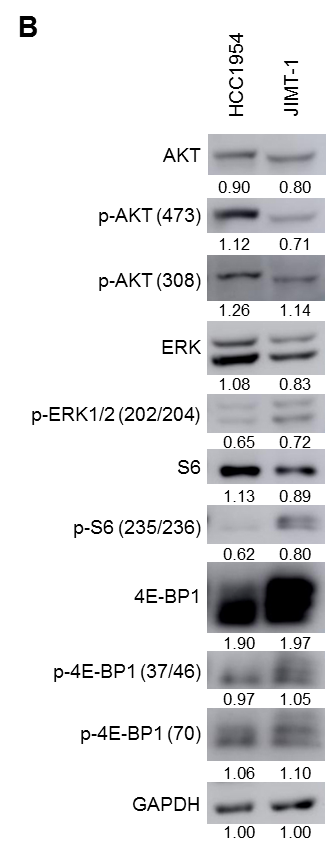
**

**Figure S2.** Characterization of a panel of cell line models of de novo trastuzumab resistance. **A.** Effect of trastuzumab treatment on primary resistant cells. Proliferation was measured after 7 days of treatment by trypan blue exclusion. Data are expressed as mean +/- SD from ≥ 3 independent experiments. *** denotes *p* ≤ 0.001. **B.** Immunoblotting analysis of primary resistant cell lines profile. Whole-cell protein extracts were analysed with the indicated antibodies. Images are representative of 3 independent experiments.


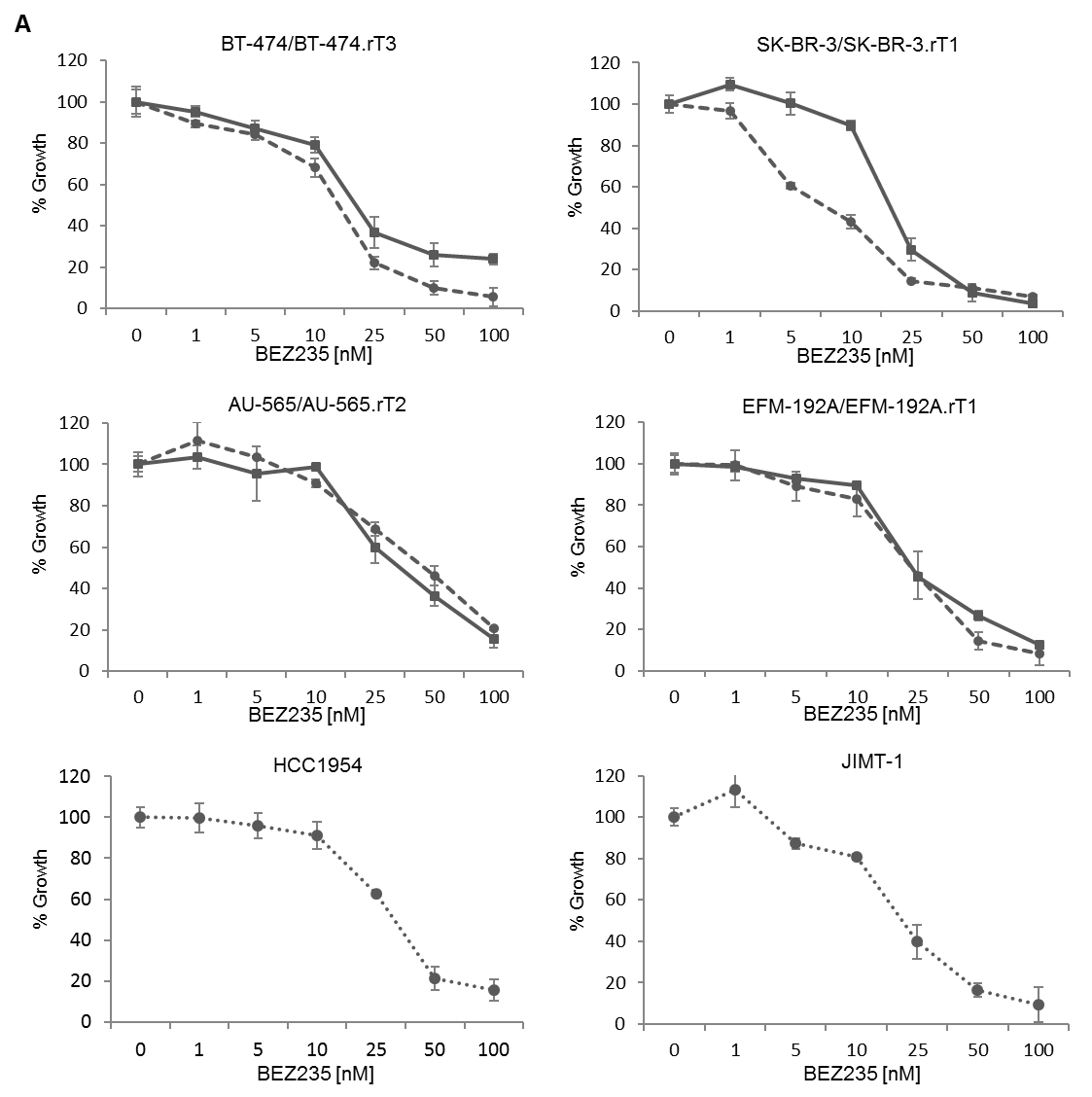


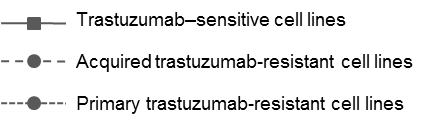


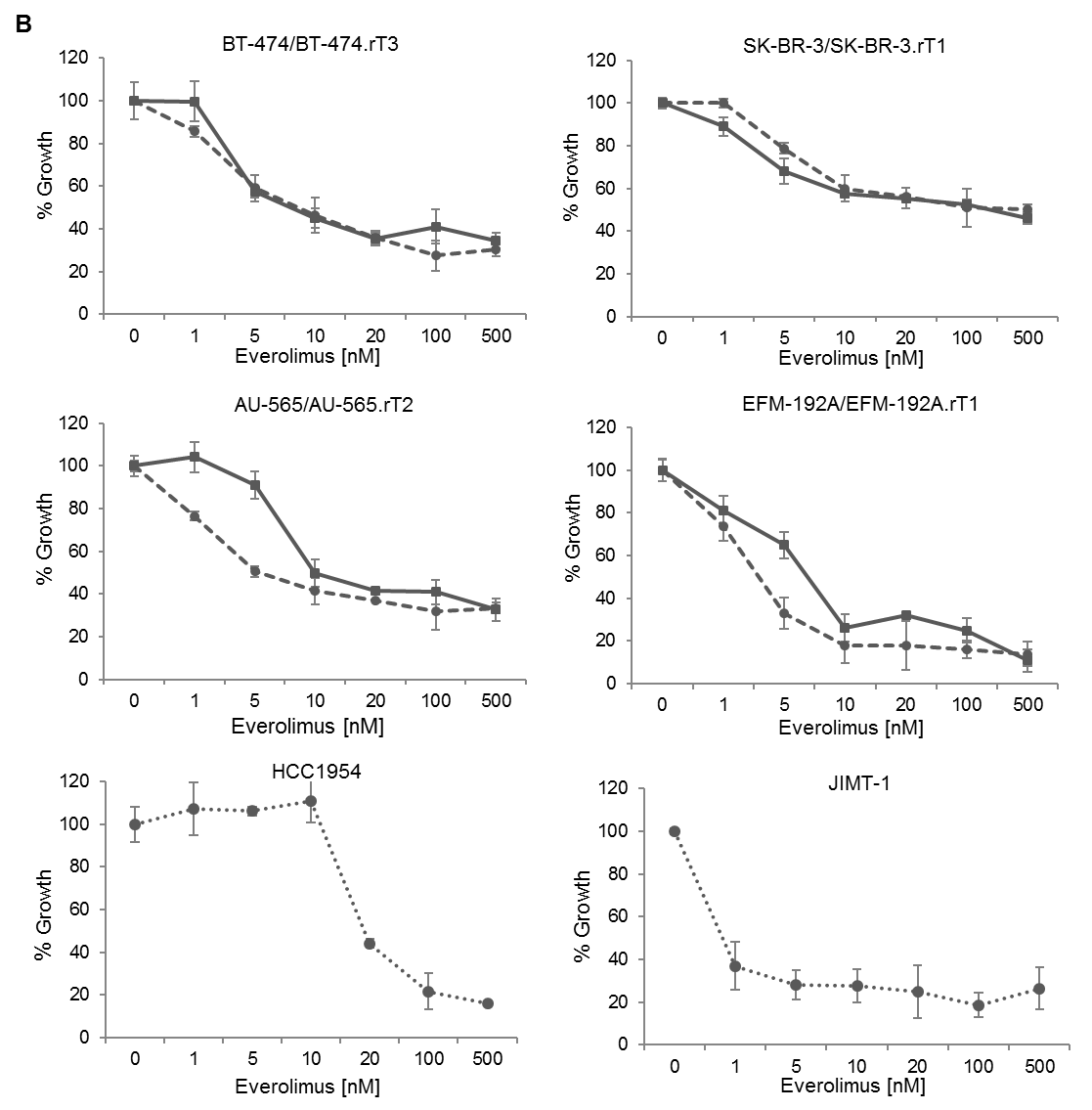


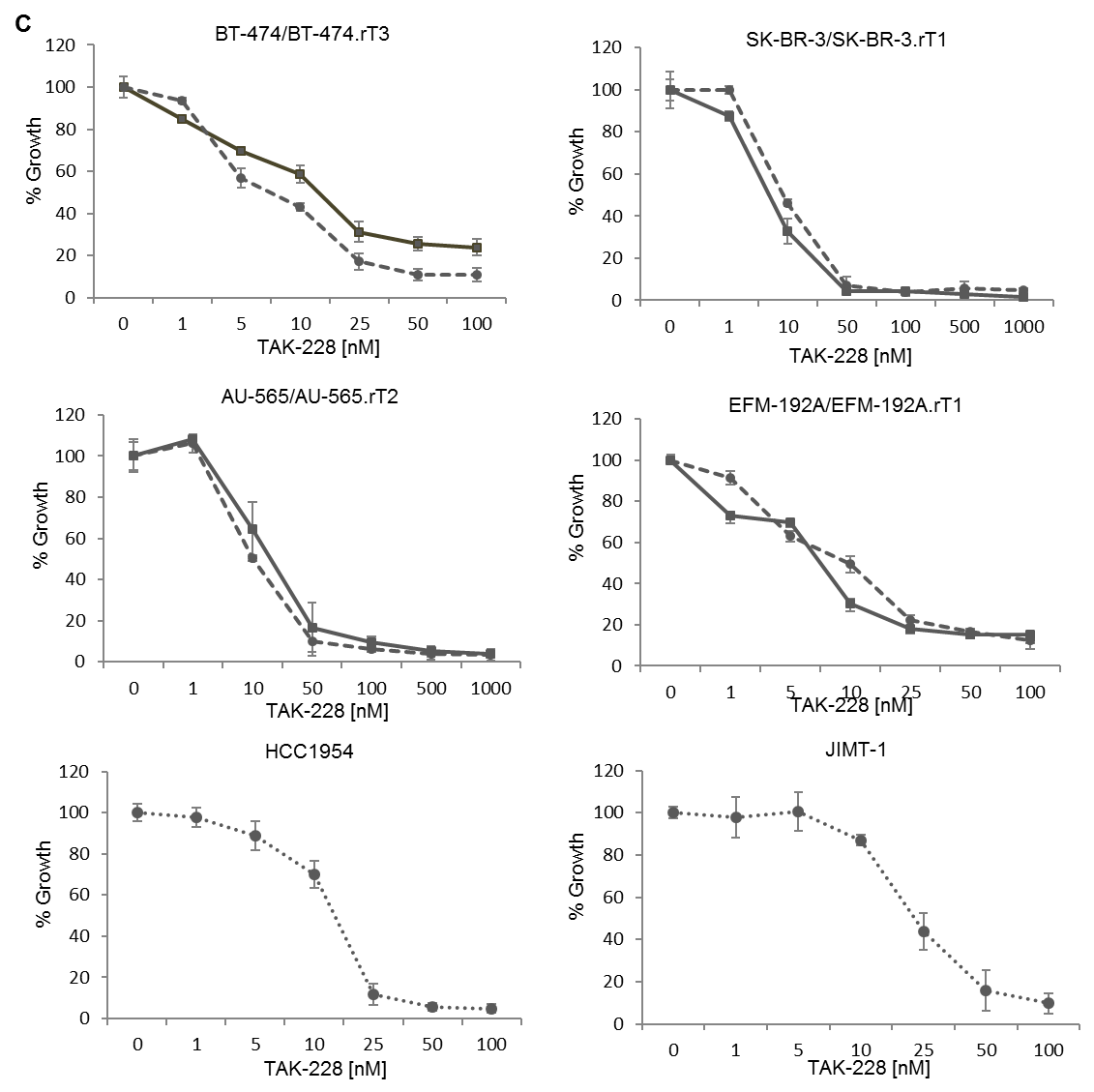


**Figure S3.** Effect of increasing concentration of PI3K/AKT/mTOR inhibitors during 7 days of treatment on cell lines BT-474, BT-474.rT3, SK-BR-3, SK-BR-3.rT1, AU-565, AU-565.rT2, EFM-192A, EFM-192A.rT1, HCC1954 and JIMT-1. **A.** Treatment with BEZ235. **B.** Treatment with everolimus. **C.** Treatment with TAK-228.


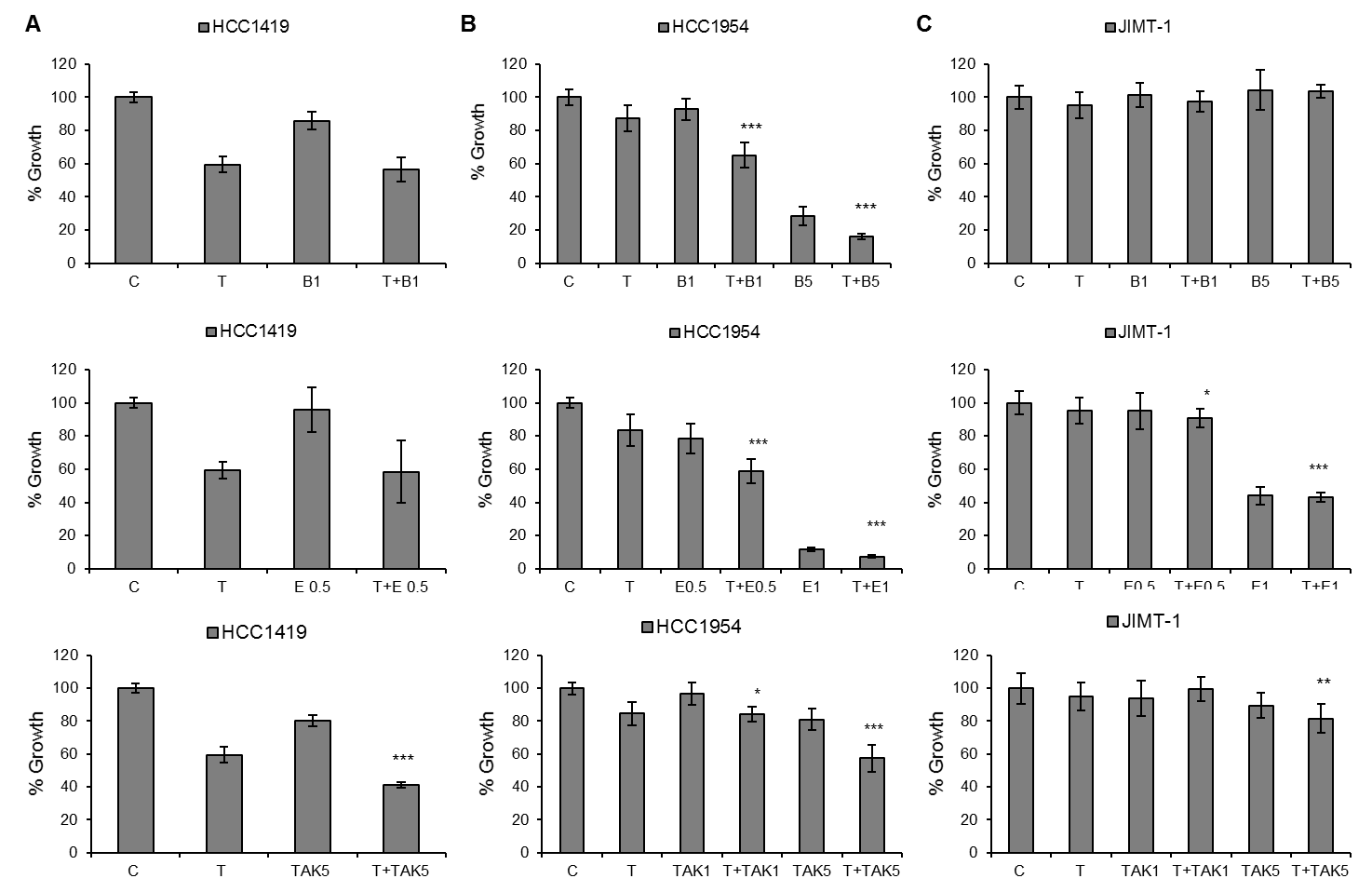


**Figure S4.** Effects of decrease in mTOR activation in trastuzumab sensitivity in primary resistant cell lines. Trastuzumab-resistant cells were treated for 7 days with DMSO, 15 μg/ml trastuzumab (T), 1 or 5 nM BEZ235 (B), 0.5 or 1 nM everolimus (E), 1 or 5 nM TAK-228 (TAK), or a combination of 15 μg/mL trastuzumab plus each mTOR inhibitor. Viable cells were then counted by trypan blue exclusion. Viability is presented as a percentage of DMSO-treated control vector group. Error bars represent standard deviation between replicates (*n* ≥ 3). * denotes *p* ≤ 0.05, ** denotes *p* ≤ 0.01 and *** denotes *p* ≤ 0.001. **A.** HCC1419 trastuzumab-sensitive/resistant cells. **B.** HCC1954 trastuzumab-resistant cells. **C.** JIMT-1 trastuzumab-resistant cells.


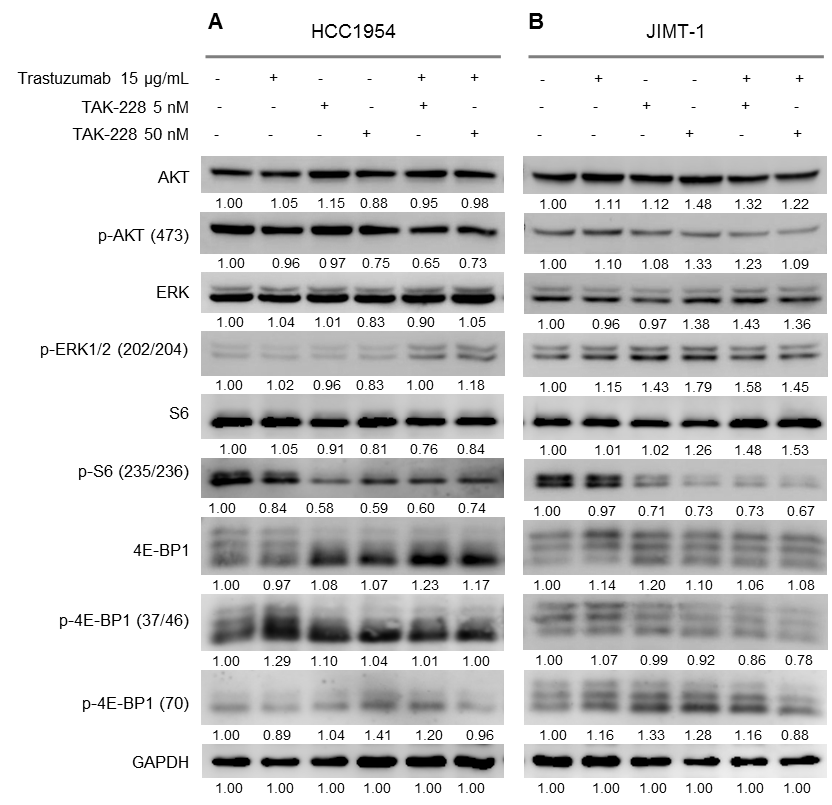


**Figure S5.** Biochemical analyses of primary trastuzumab-resistant cells treated with trastuzumab and TAK-228. HCC1954 and JIMT-1 cells were treated for 24 h with DMSO, 15 μg/mL trastuzumab (T), 5 or 50 nM TAK-228 (TAK), or a combination of 15 μg/mL trastuzumab plus 5 or 50 nM TAK-228. Whole-cell protein extracts were analysed with the indicated antibodies. Images are representative of 3 independent experiments.
