# Supplementary figures and images for: The Novel Oral mTORC1/2 Inhibitor TAK-228 Reverses Trastuzumab Resistance in HER2-Positive Breast Cancer Models (part 2 of 2)
Source: Cancers (Basel). 2021 Jun 3;13(11):2778. doi: 10.3390/cancers13112778 (PMC8199905; doi:10.3390/cancers13112778)

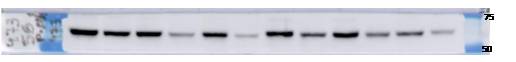

Supplement: Supplementary file 1 [file cancers-13-02778-s001.zip › Figure.S6/Figure5/SKBR3-SKBR3.rT1/pAKT 473 MW.jpg]

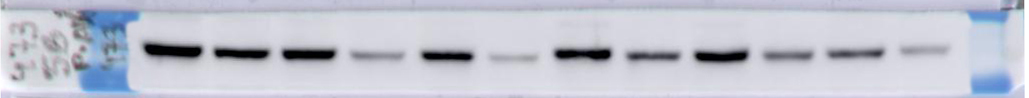

Supplement: Supplementary file 1 [file cancers-13-02778-s001.zip › Figure.S6/Figure5/SKBR3-SKBR3.rT1/pAKT 473.jpg]

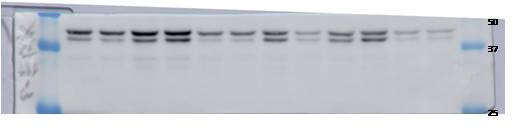

Supplement: Supplementary file 1 [file cancers-13-02778-s001.zip › Figure.S6/Figure5/SKBR3-SKBR3.rT1/pERK MW.jpg]

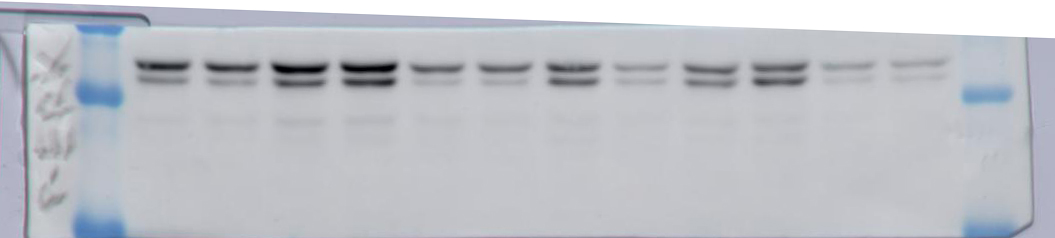

Supplement: Supplementary file 1 [file cancers-13-02778-s001.zip › Figure.S6/Figure5/SKBR3-SKBR3.rT1/pERK.jpg]

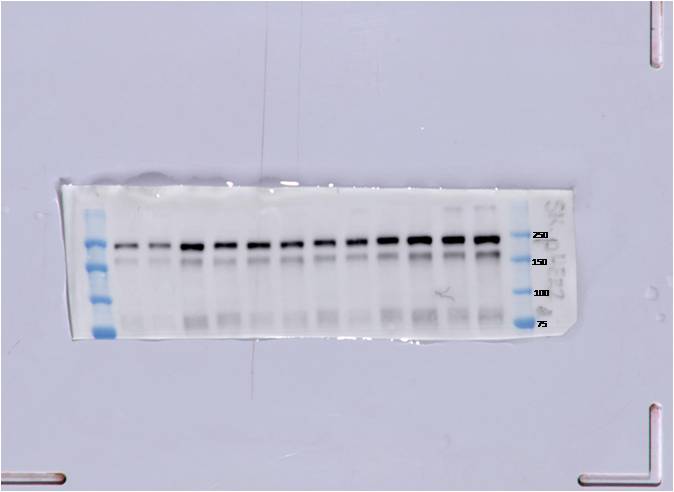

Supplement: Supplementary file 1 [file cancers-13-02778-s001.zip › Figure.S6/Figure5/SKBR3-SKBR3.rT1/pHER2 MW.jpg]

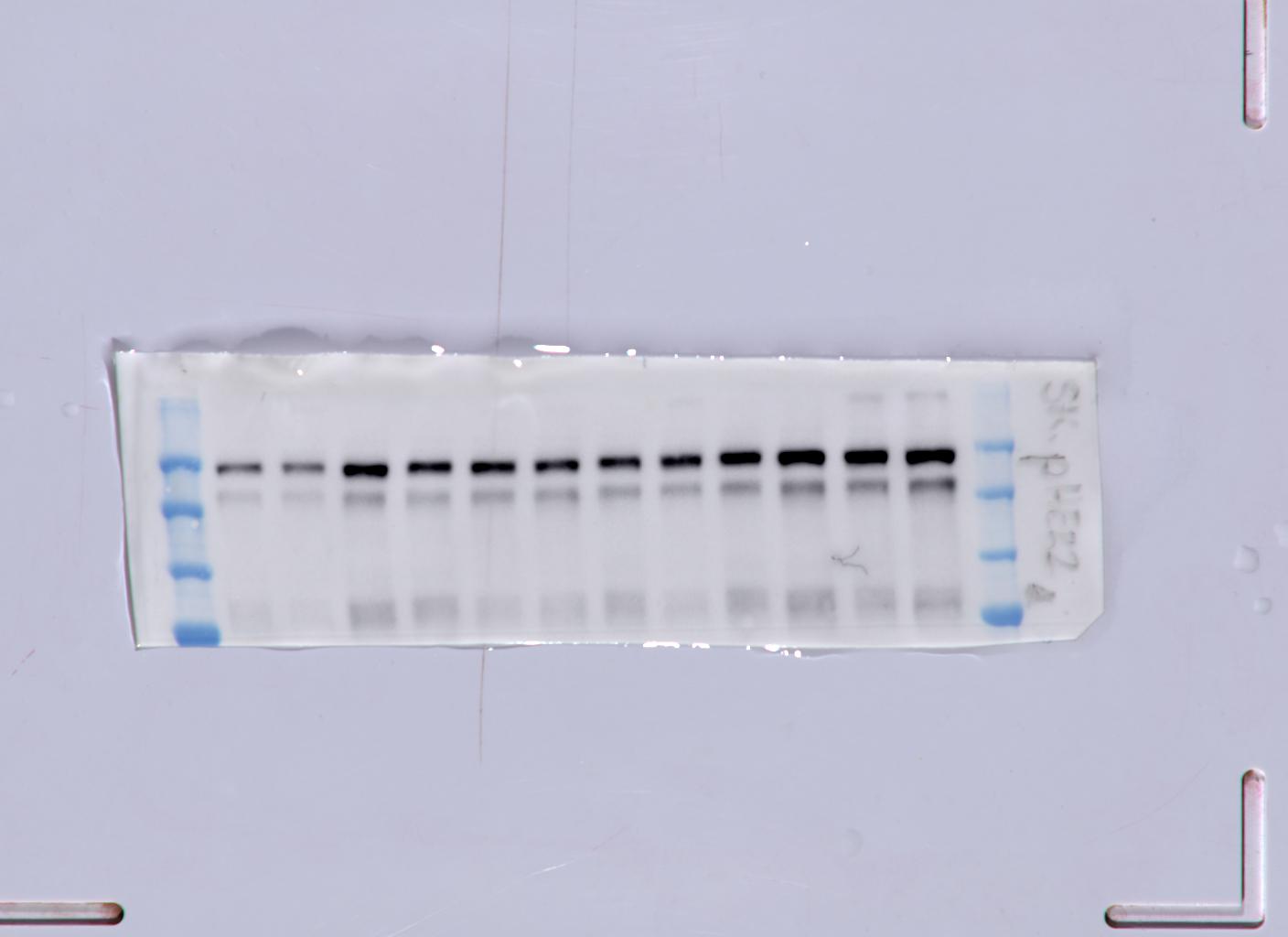

Supplement: Supplementary file 1 [file cancers-13-02778-s001.zip › Figure.S6/Figure5/SKBR3-SKBR3.rT1/pHER2.jpg]

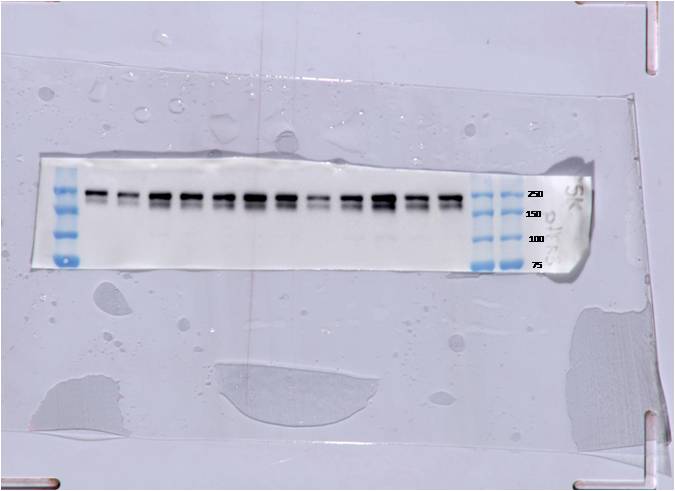

Supplement: Supplementary file 1 [file cancers-13-02778-s001.zip › Figure.S6/Figure5/SKBR3-SKBR3.rT1/pHER3 MW.jpg]

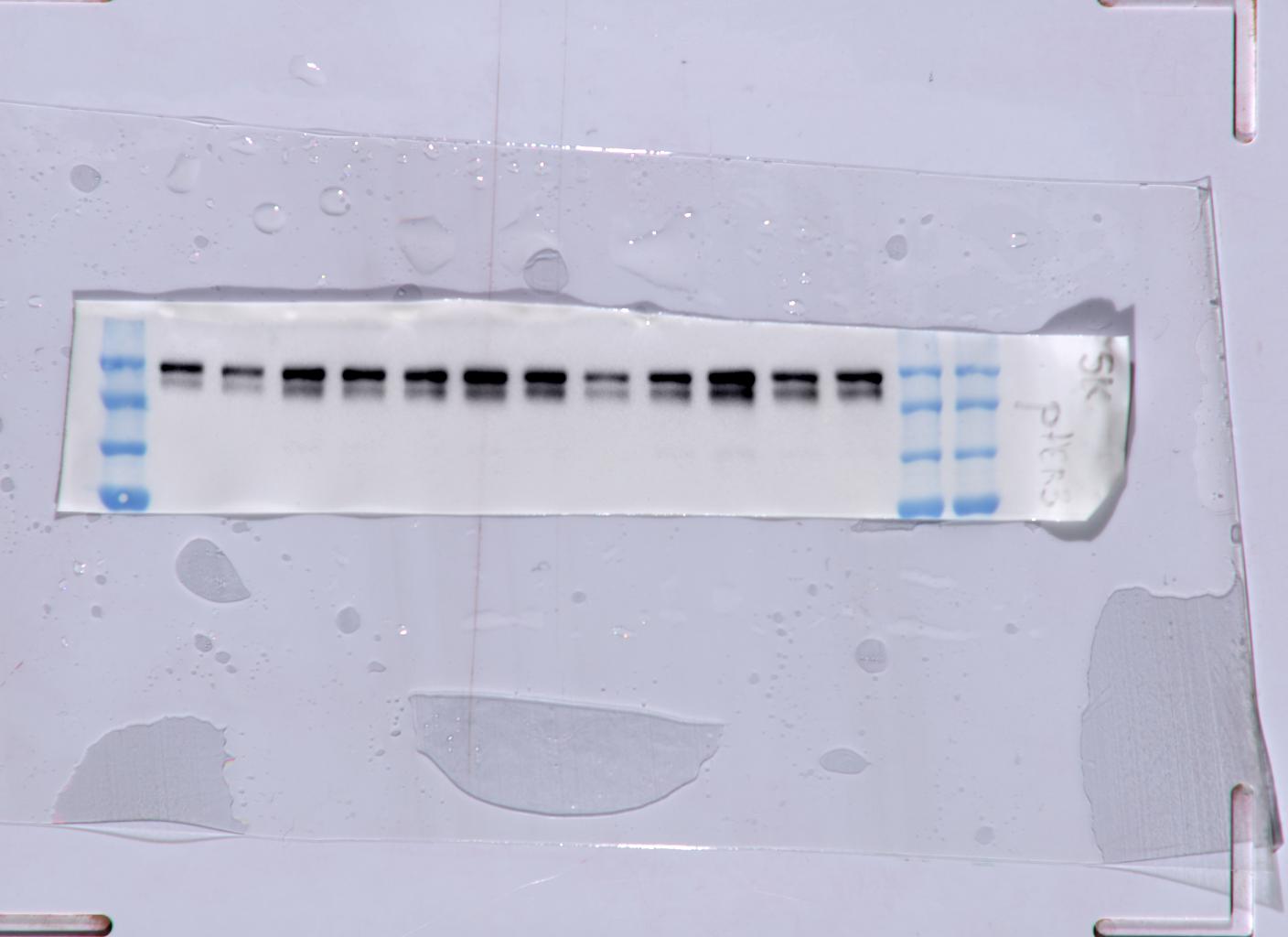

Supplement: Supplementary file 1 [file cancers-13-02778-s001.zip › Figure.S6/Figure5/SKBR3-SKBR3.rT1/pHER3.jpg]

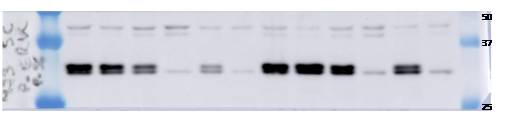

Supplement: Supplementary file 1 [file cancers-13-02778-s001.zip › Figure.S6/Figure5/SKBR3-SKBR3.rT1/pS6 MW.jpg]

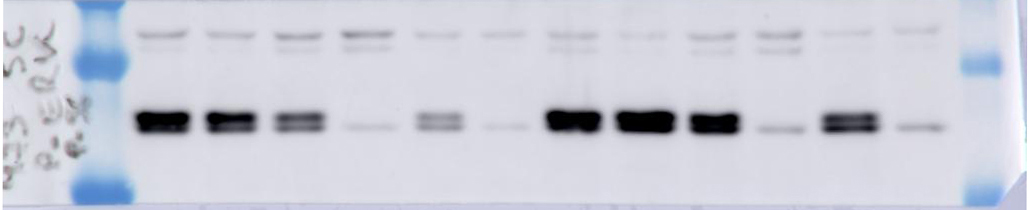

Supplement: Supplementary file 1 [file cancers-13-02778-s001.zip › Figure.S6/Figure5/SKBR3-SKBR3.rT1/pS6.jpg]

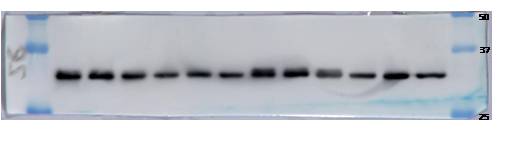

Supplement: Supplementary file 1 [file cancers-13-02778-s001.zip › Figure.S6/Figure5/SKBR3-SKBR3.rT1/S6 MW.jpg]

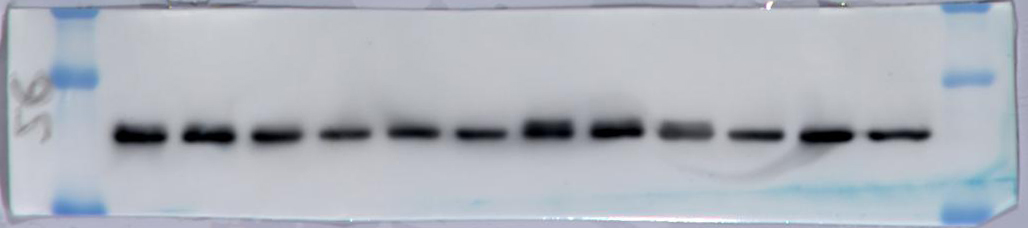

Supplement: Supplementary file 1 [file cancers-13-02778-s001.zip › Figure.S6/Figure5/SKBR3-SKBR3.rT1/S6.jpg]

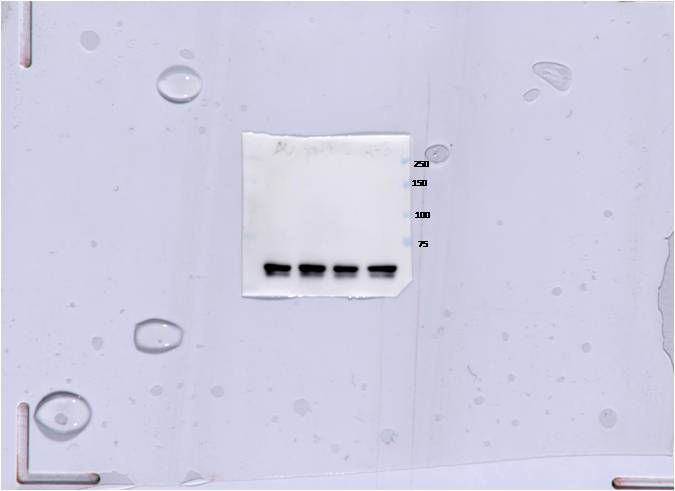

Supplement: Supplementary file 1 [file cancers-13-02778-s001.zip › Figure.S6/FigureS1/AU-565/AKT MW.jpg]

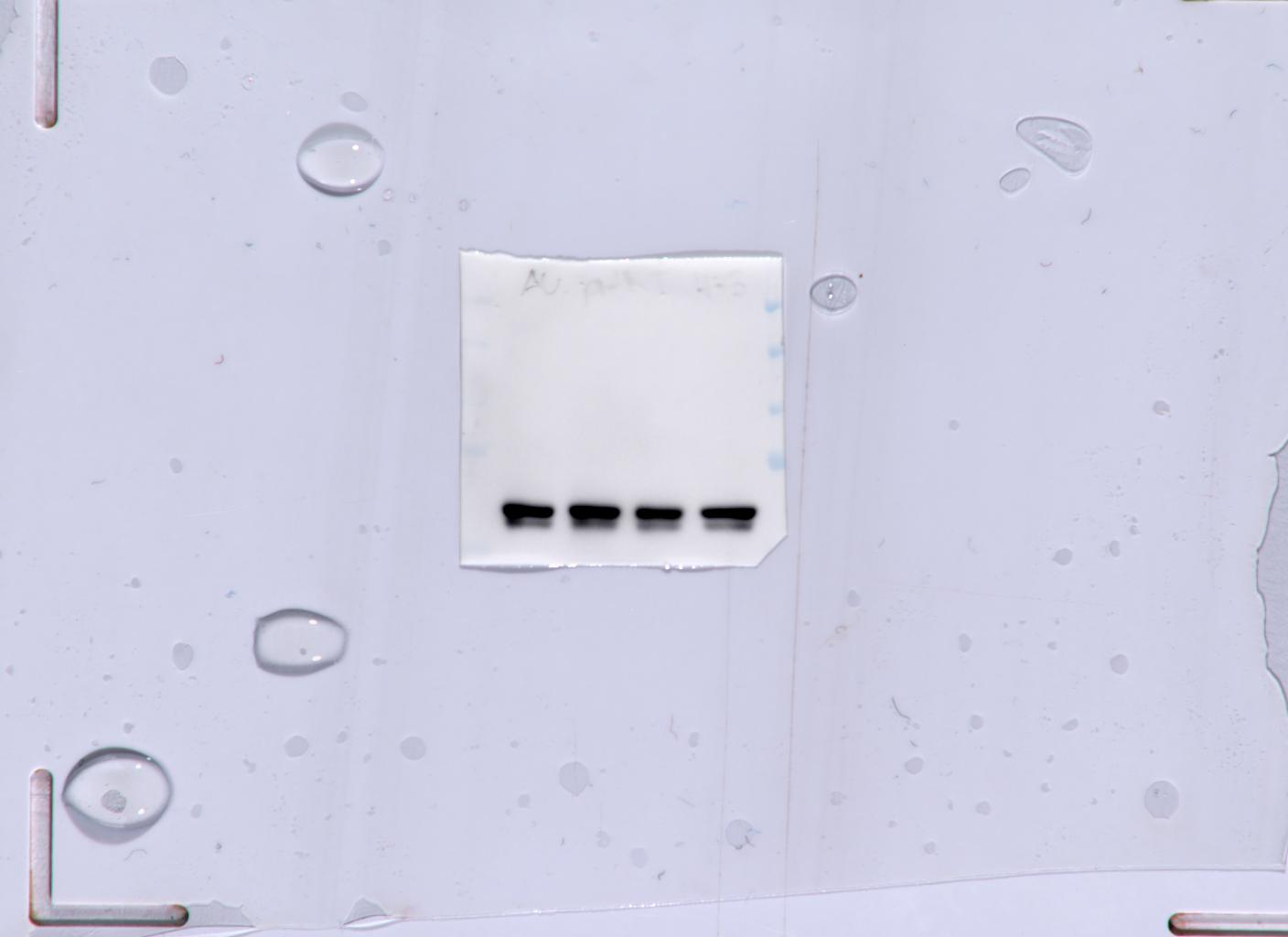

Supplement: Supplementary file 1 [file cancers-13-02778-s001.zip › Figure.S6/FigureS1/AU-565/AKT.jpg]

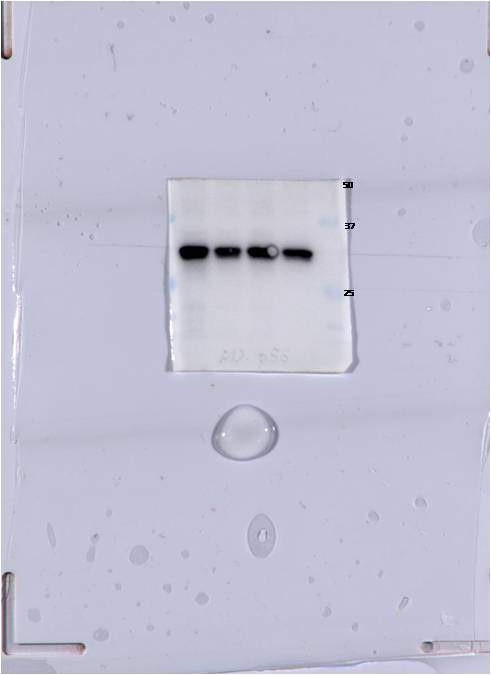

Supplement: Supplementary file 1 [file cancers-13-02778-s001.zip › Figure.S6/FigureS1/AU-565/AU565.pS6 MW.jpg]

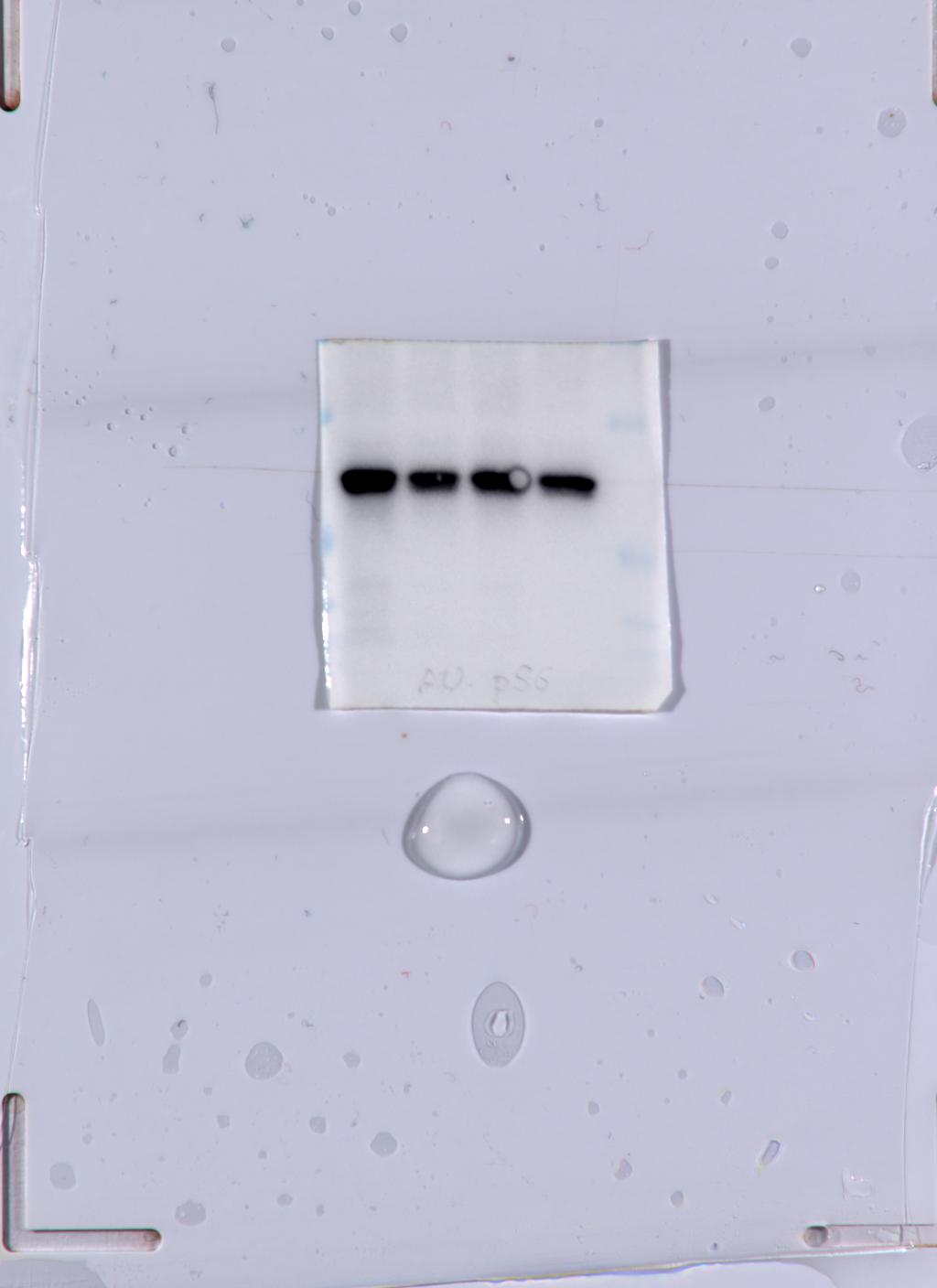

Supplement: Supplementary file 1 [file cancers-13-02778-s001.zip › Figure.S6/FigureS1/AU-565/AU565.pS6.jpg]

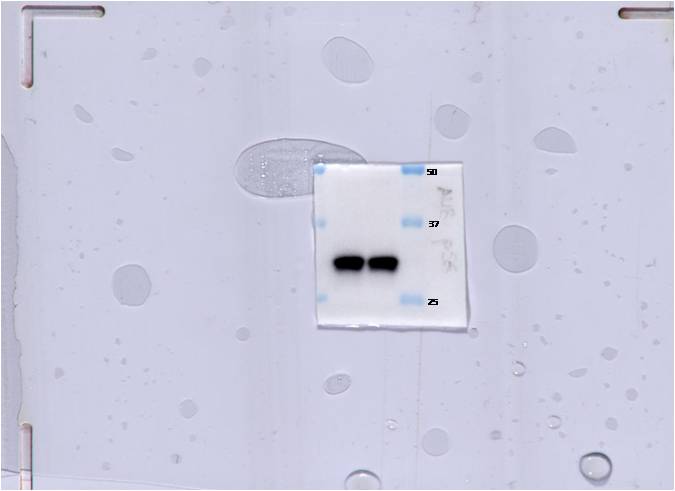

Supplement: Supplementary file 1 [file cancers-13-02778-s001.zip › Figure.S6/FigureS1/AU-565/AU565.rT2.pS6 MW.jpg]

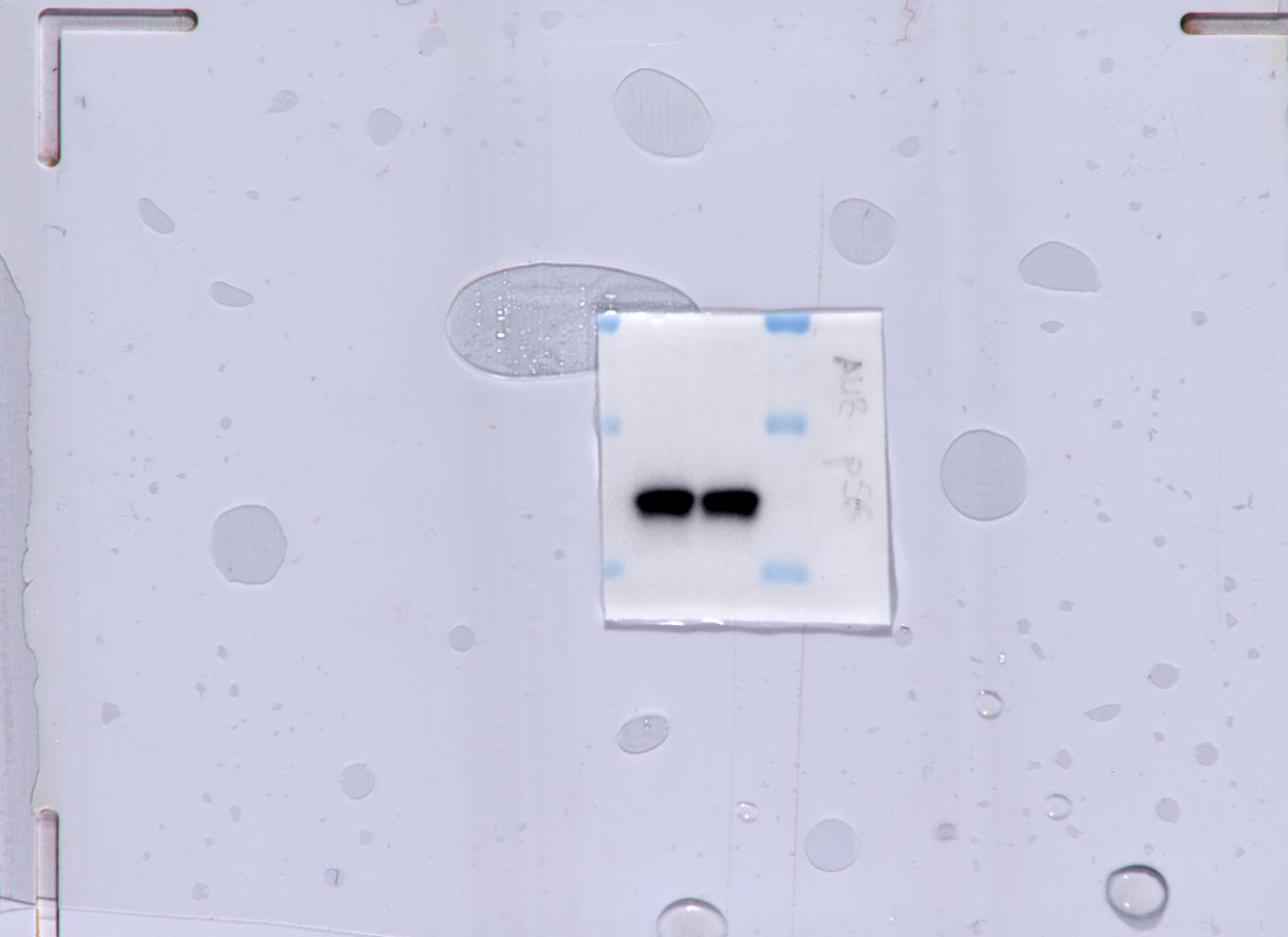

Supplement: Supplementary file 1 [file cancers-13-02778-s001.zip › Figure.S6/FigureS1/AU-565/AU565.rT2.pS6.jpg]

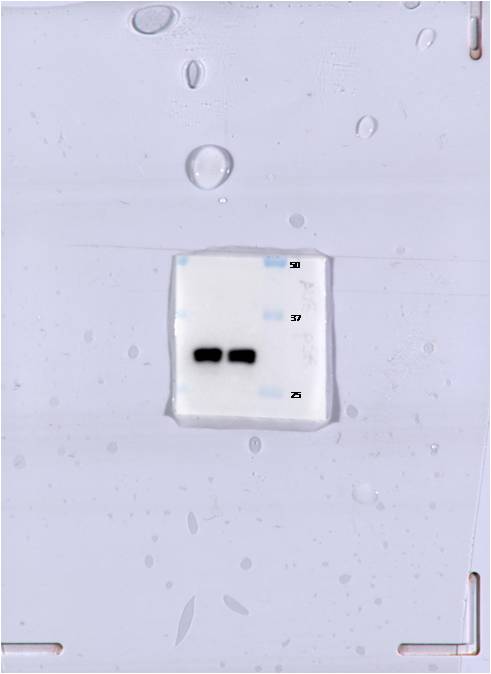

Supplement: Supplementary file 1 [file cancers-13-02778-s001.zip › Figure.S6/FigureS1/AU-565/AU565.rT2.S6 MW.jpg]

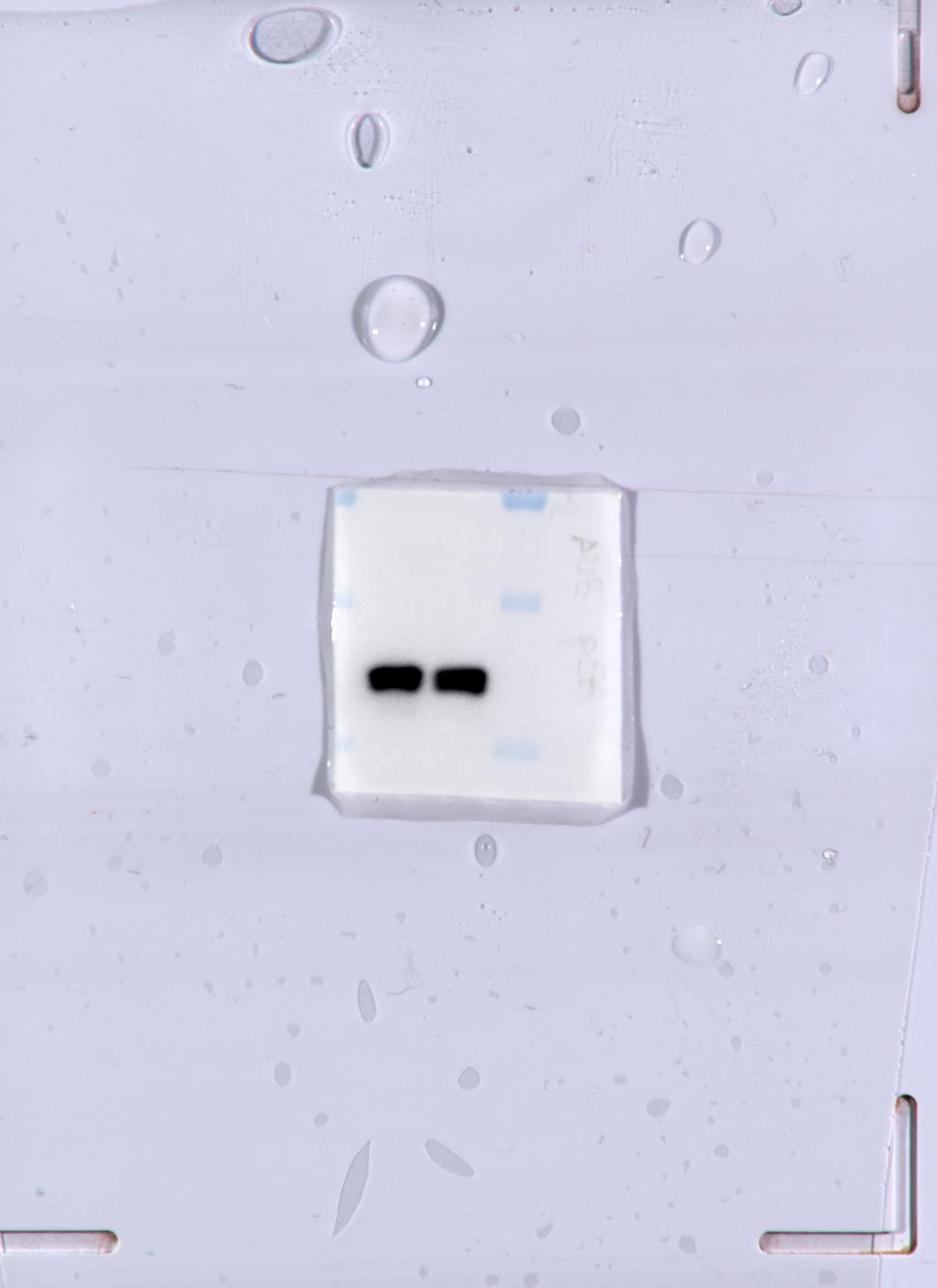

Supplement: Supplementary file 1 [file cancers-13-02778-s001.zip › Figure.S6/FigureS1/AU-565/AU565.rT2.S6.jpg]

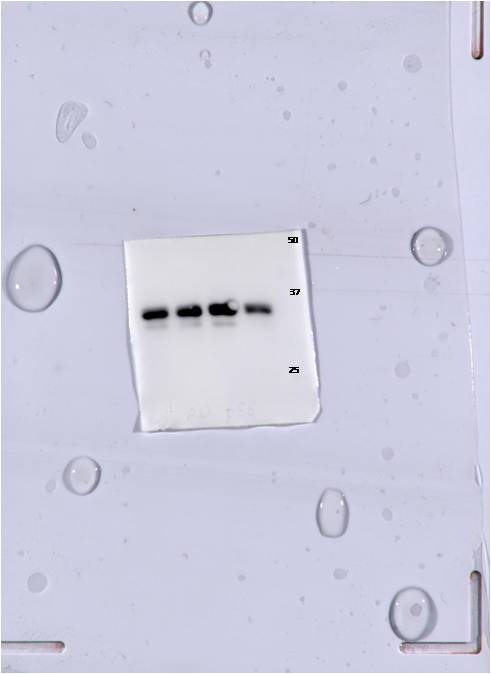

Supplement: Supplementary file 1 [file cancers-13-02778-s001.zip › Figure.S6/FigureS1/AU-565/AU565.S6 MW.jpg]

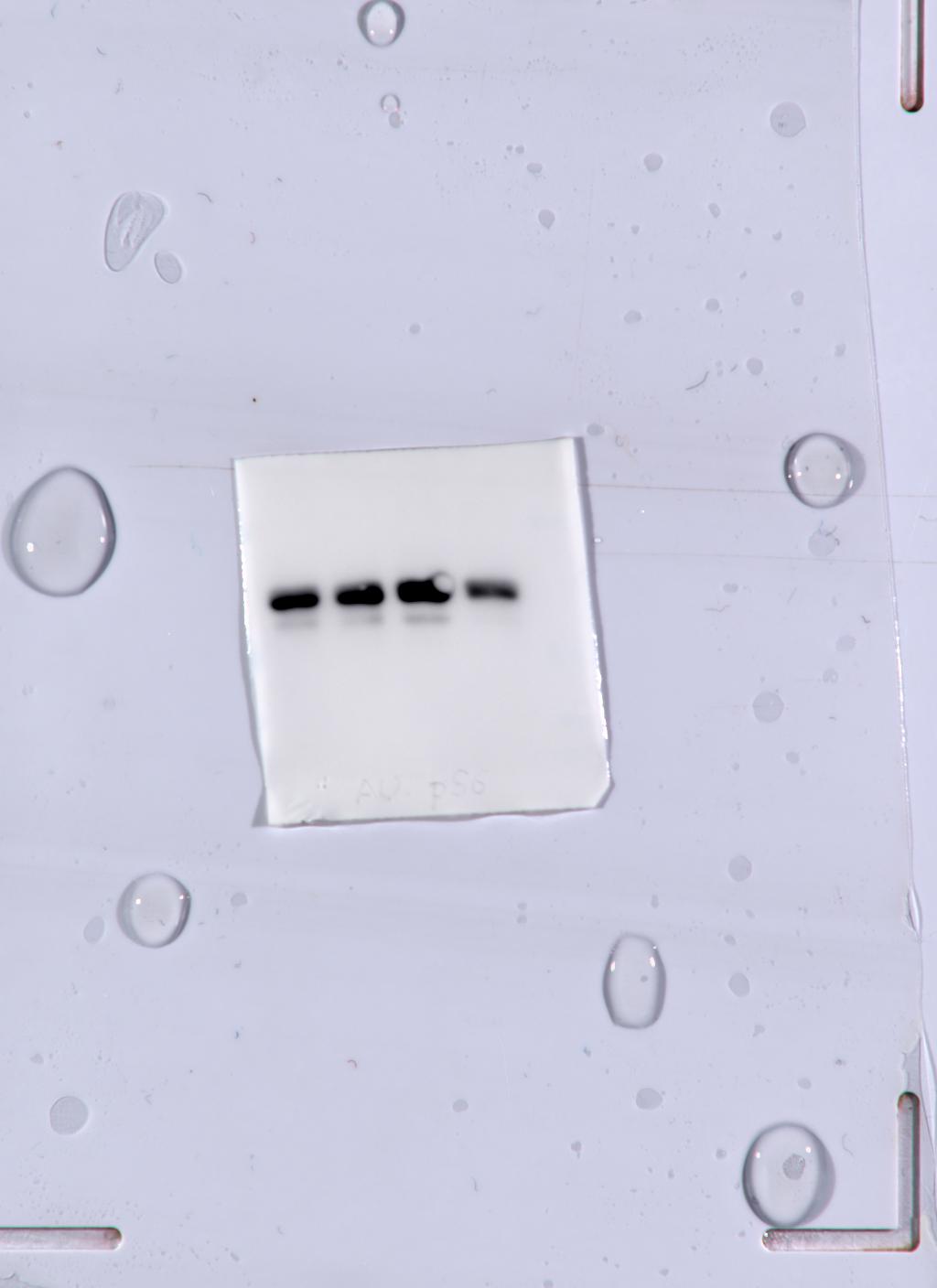

Supplement: Supplementary file 1 [file cancers-13-02778-s001.zip › Figure.S6/FigureS1/AU-565/AU565.S6.jpg]

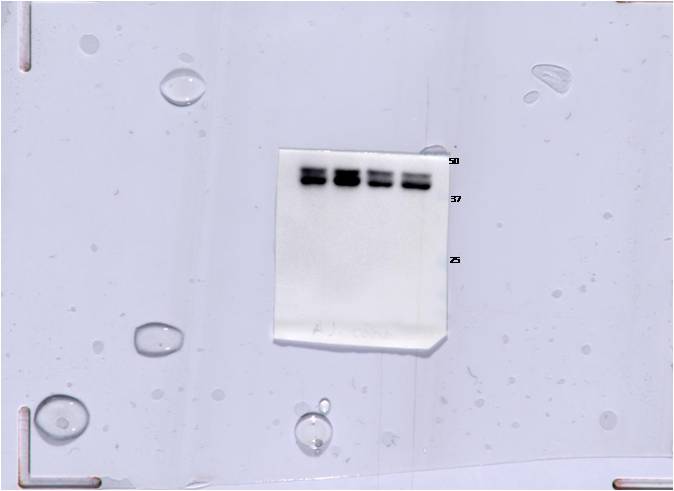

Supplement: Supplementary file 1 [file cancers-13-02778-s001.zip › Figure.S6/FigureS1/AU-565/ERK MW.jpg]

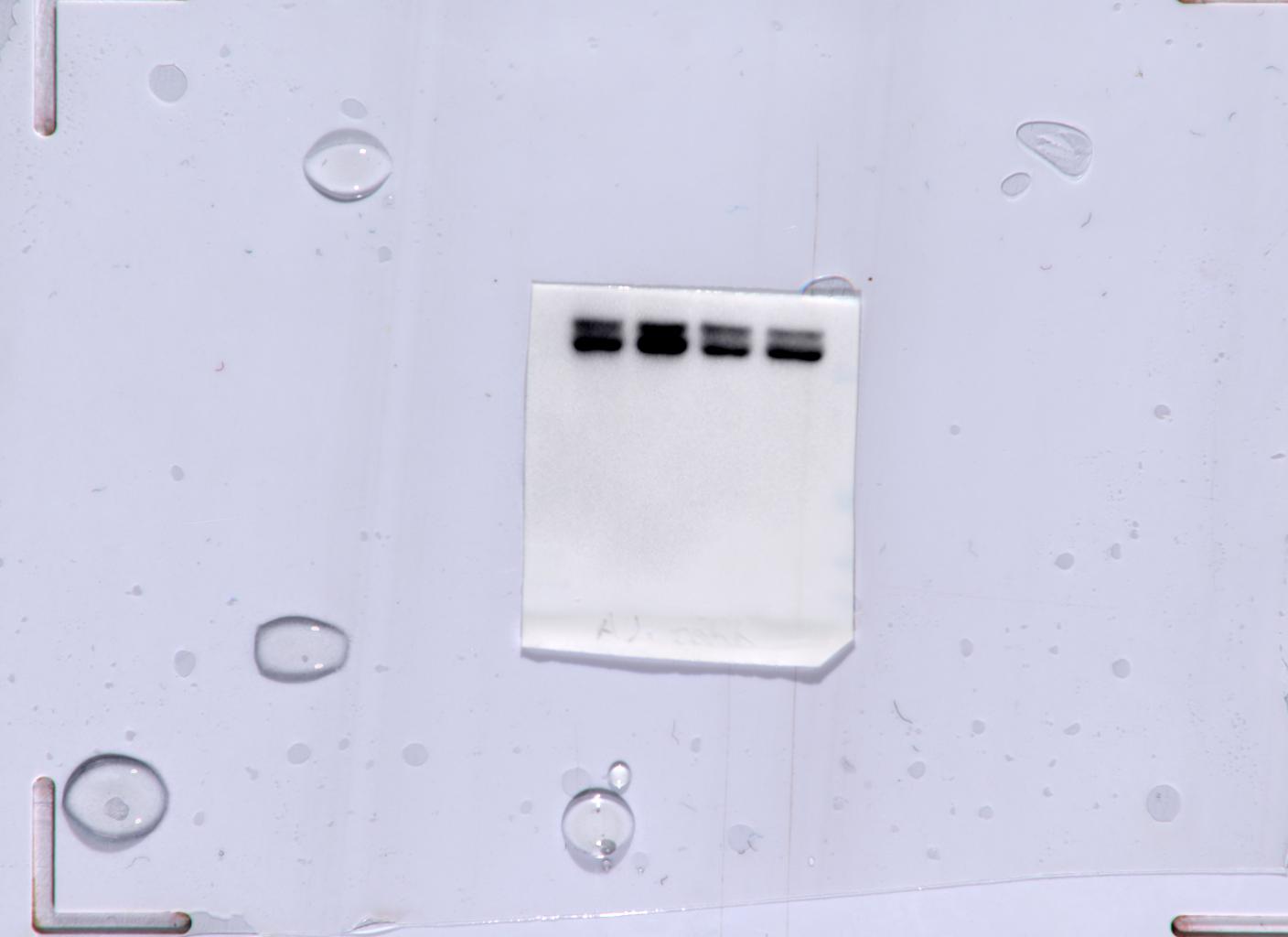

Supplement: Supplementary file 1 [file cancers-13-02778-s001.zip › Figure.S6/FigureS1/AU-565/ERK.jpg]

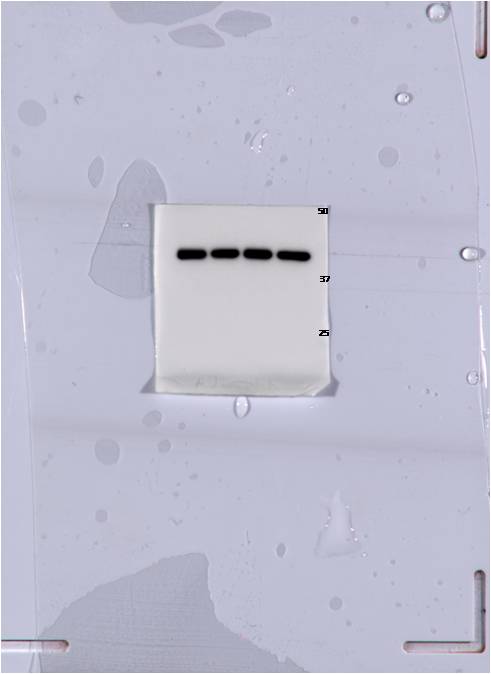

Supplement: Supplementary file 1 [file cancers-13-02778-s001.zip › Figure.S6/FigureS1/AU-565/GAPDH MW.jpg]

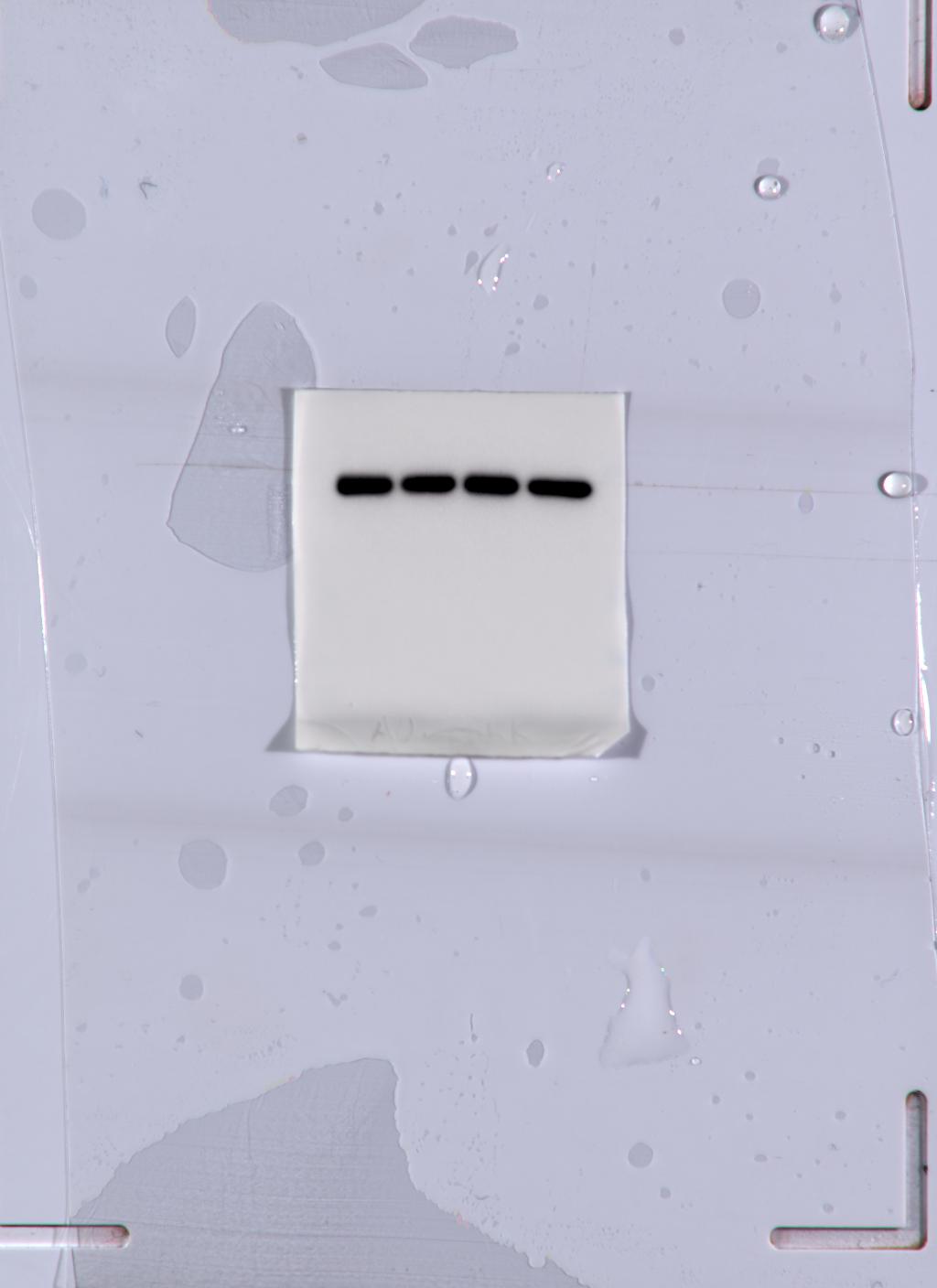

Supplement: Supplementary file 1 [file cancers-13-02778-s001.zip › Figure.S6/FigureS1/AU-565/GAPDH.jpg]

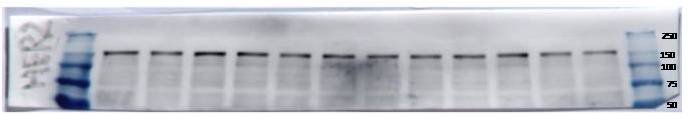

Supplement: Supplementary file 1 [file cancers-13-02778-s001.zip › Figure.S6/FigureS1/AU-565/HER2 MW.jpg]

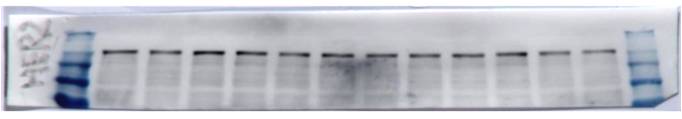

Supplement: Supplementary file 1 [file cancers-13-02778-s001.zip › Figure.S6/FigureS1/AU-565/HER2.jpg]

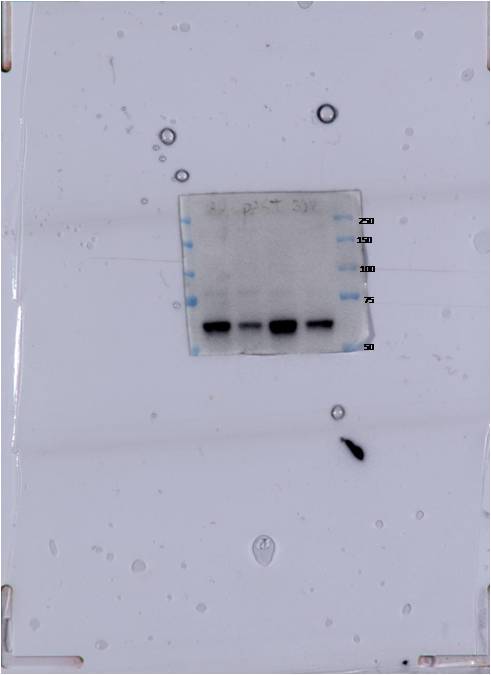

Supplement: Supplementary file 1 [file cancers-13-02778-s001.zip › Figure.S6/FigureS1/AU-565/pAKT 308 MW.jpg]

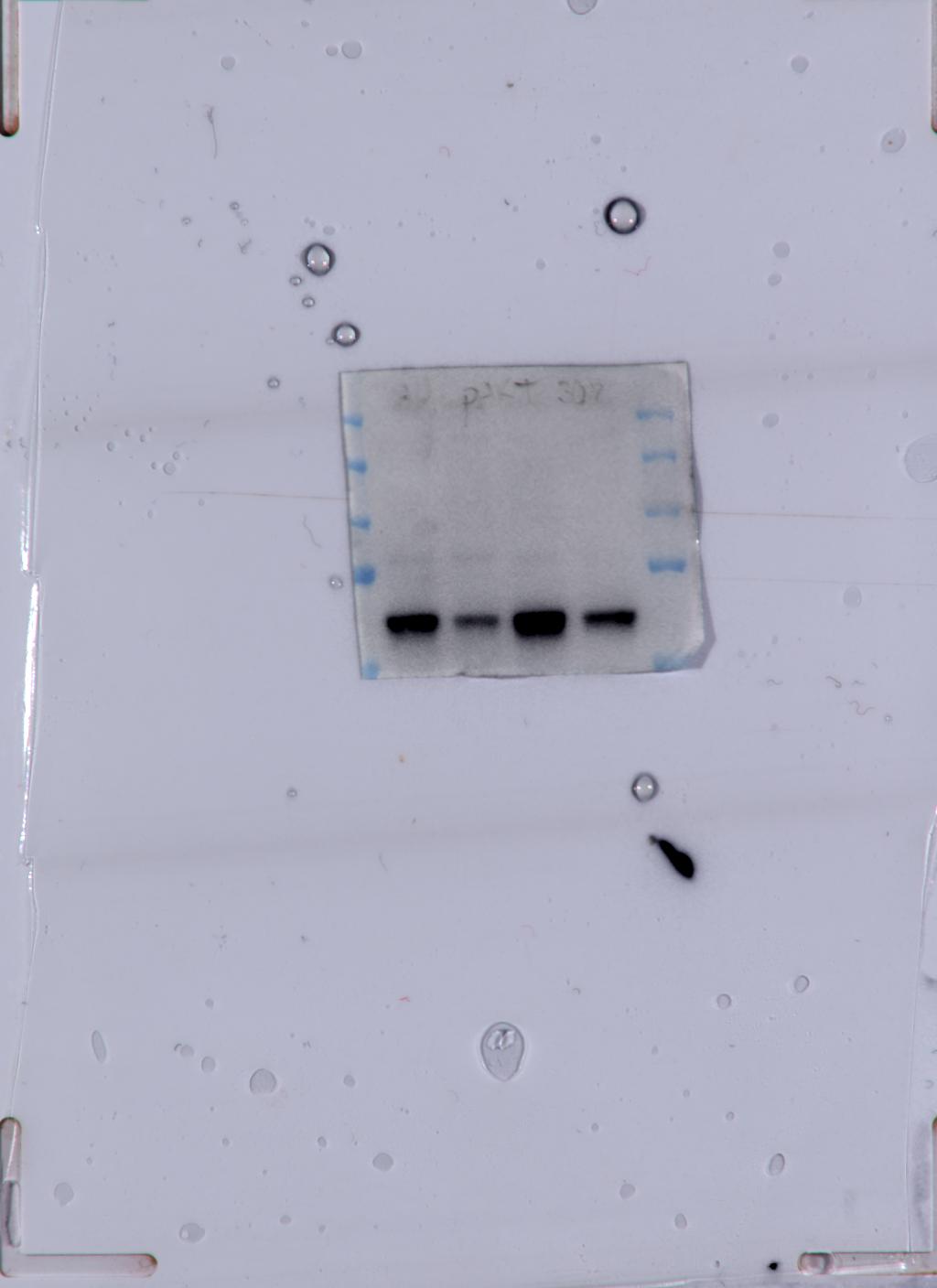

Supplement: Supplementary file 1 [file cancers-13-02778-s001.zip › Figure.S6/FigureS1/AU-565/pAKT 308.jpg]

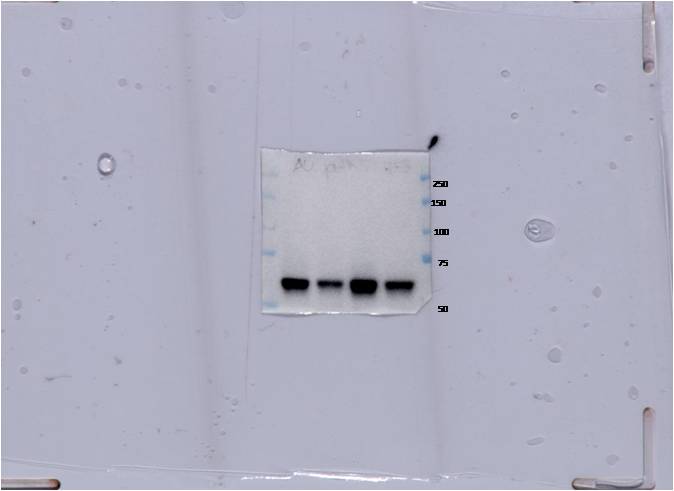

Supplement: Supplementary file 1 [file cancers-13-02778-s001.zip › Figure.S6/FigureS1/AU-565/pAKT 473 MW.jpg]

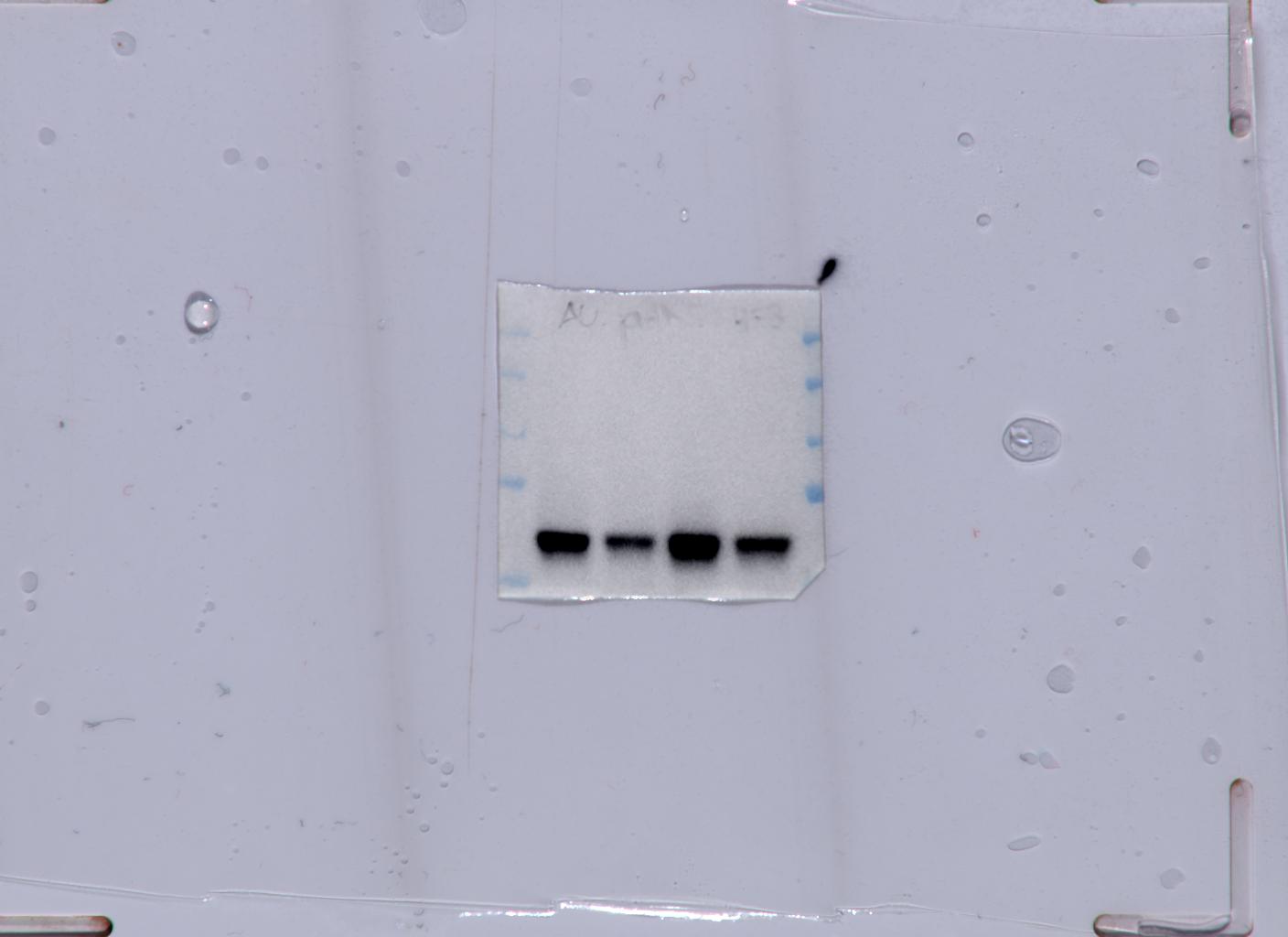

Supplement: Supplementary file 1 [file cancers-13-02778-s001.zip › Figure.S6/FigureS1/AU-565/pAKT 473.jpg]

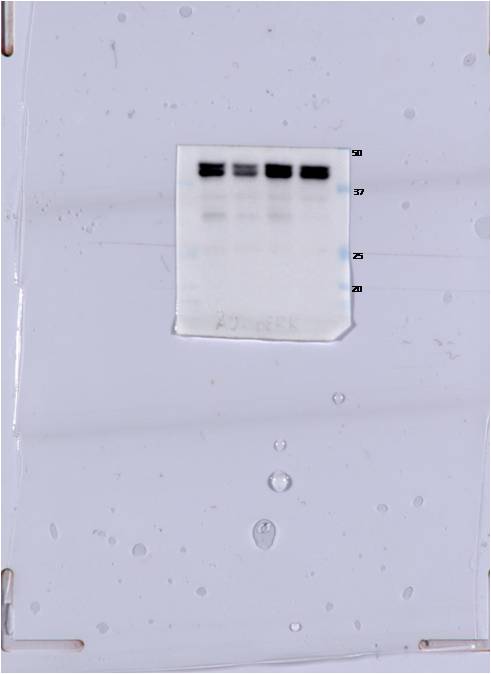

Supplement: Supplementary file 1 [file cancers-13-02778-s001.zip › Figure.S6/FigureS1/AU-565/pERK MW.jpg]

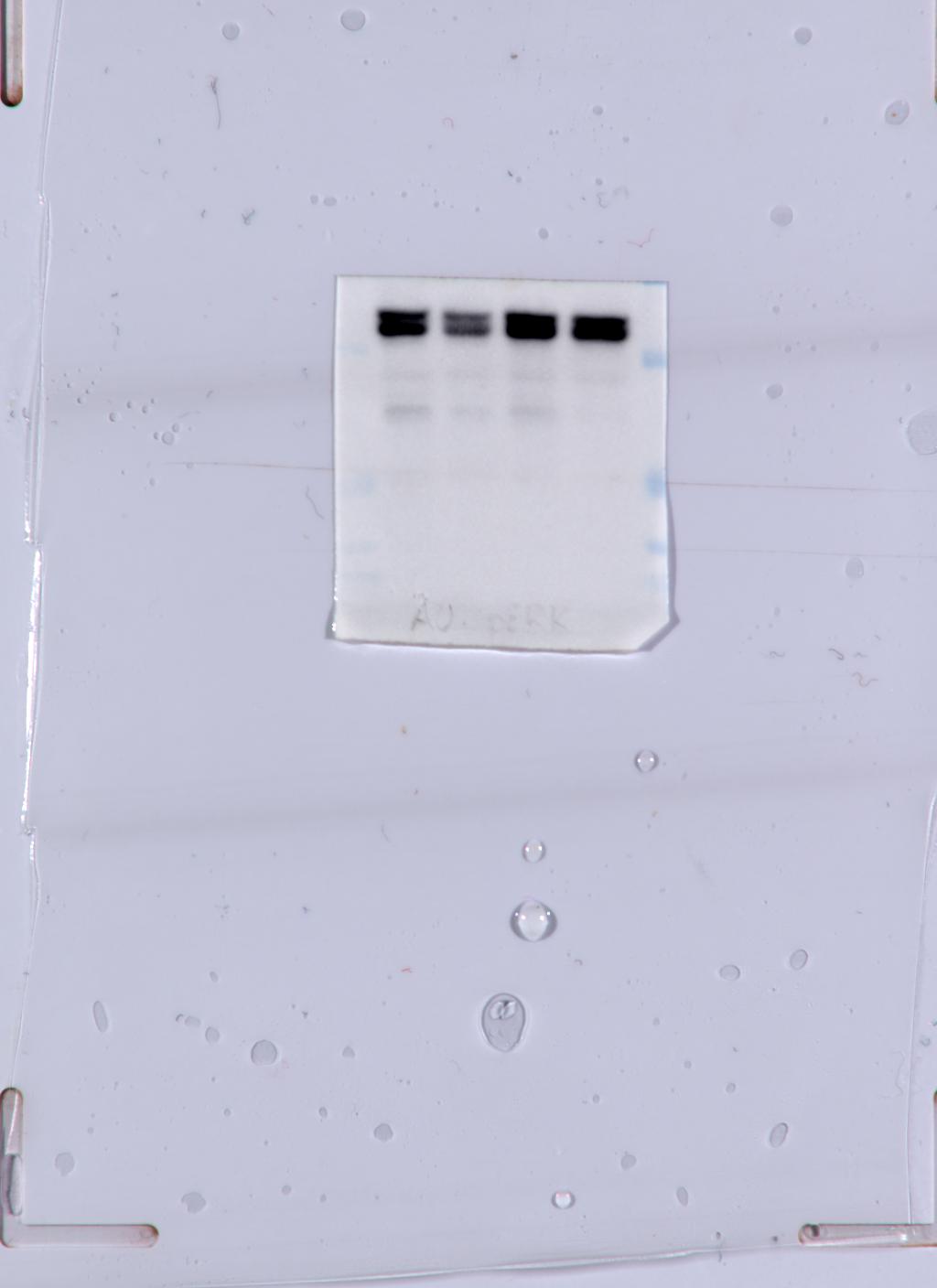

Supplement: Supplementary file 1 [file cancers-13-02778-s001.zip › Figure.S6/FigureS1/AU-565/pERK.jpg]

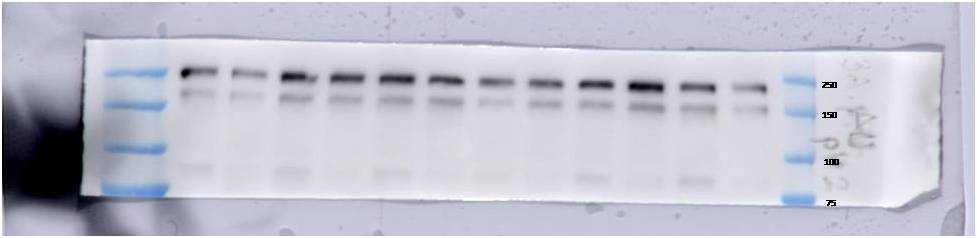

Supplement: Supplementary file 1 [file cancers-13-02778-s001.zip › Figure.S6/FigureS1/AU-565/pHER2 MW.jpg]

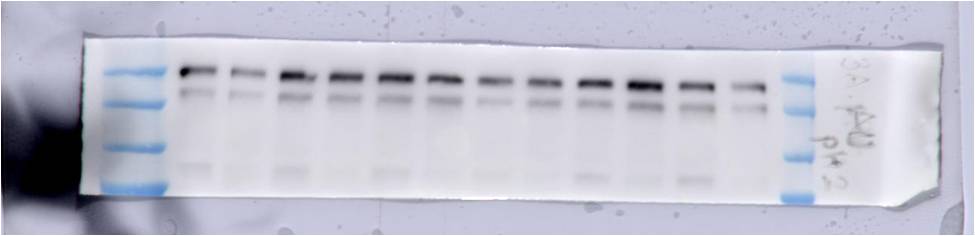

Supplement: Supplementary file 1 [file cancers-13-02778-s001.zip › Figure.S6/FigureS1/AU-565/pHER2.jpg]

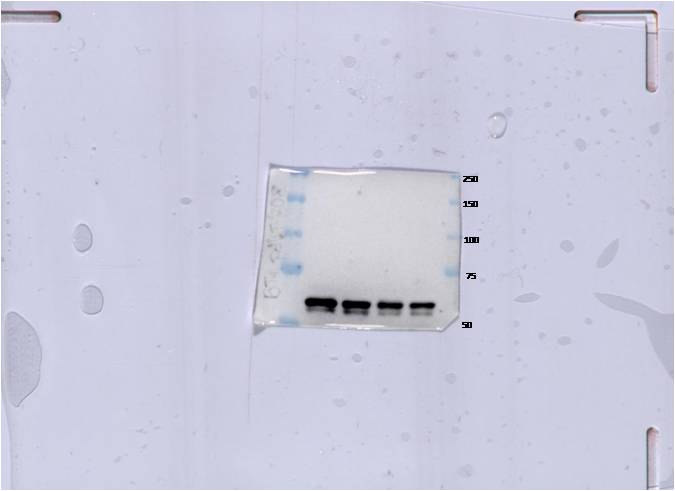

Supplement: Supplementary file 1 [file cancers-13-02778-s001.zip › Figure.S6/FigureS1/BT-474/AKT MW.jpg]

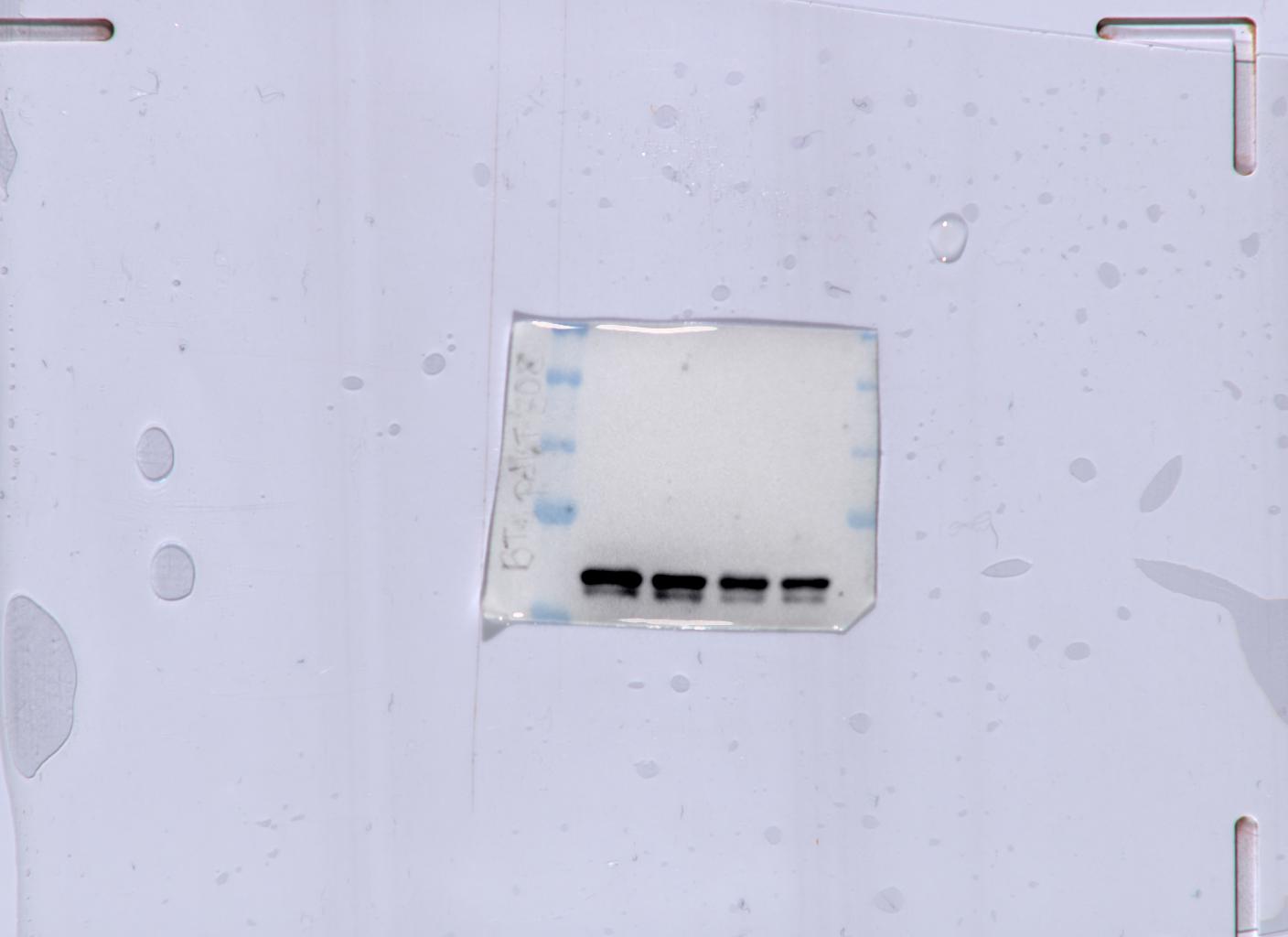

Supplement: Supplementary file 1 [file cancers-13-02778-s001.zip › Figure.S6/FigureS1/BT-474/AKT.jpg]

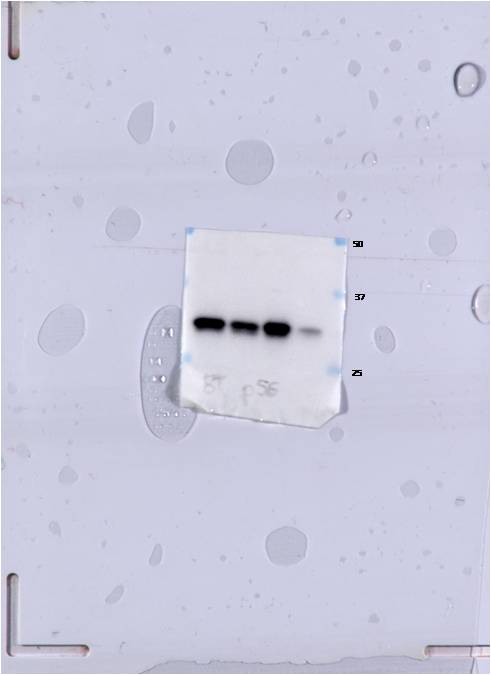

Supplement: Supplementary file 1 [file cancers-13-02778-s001.zip › Figure.S6/FigureS1/BT-474/BT474.pS6 MW.jpg]

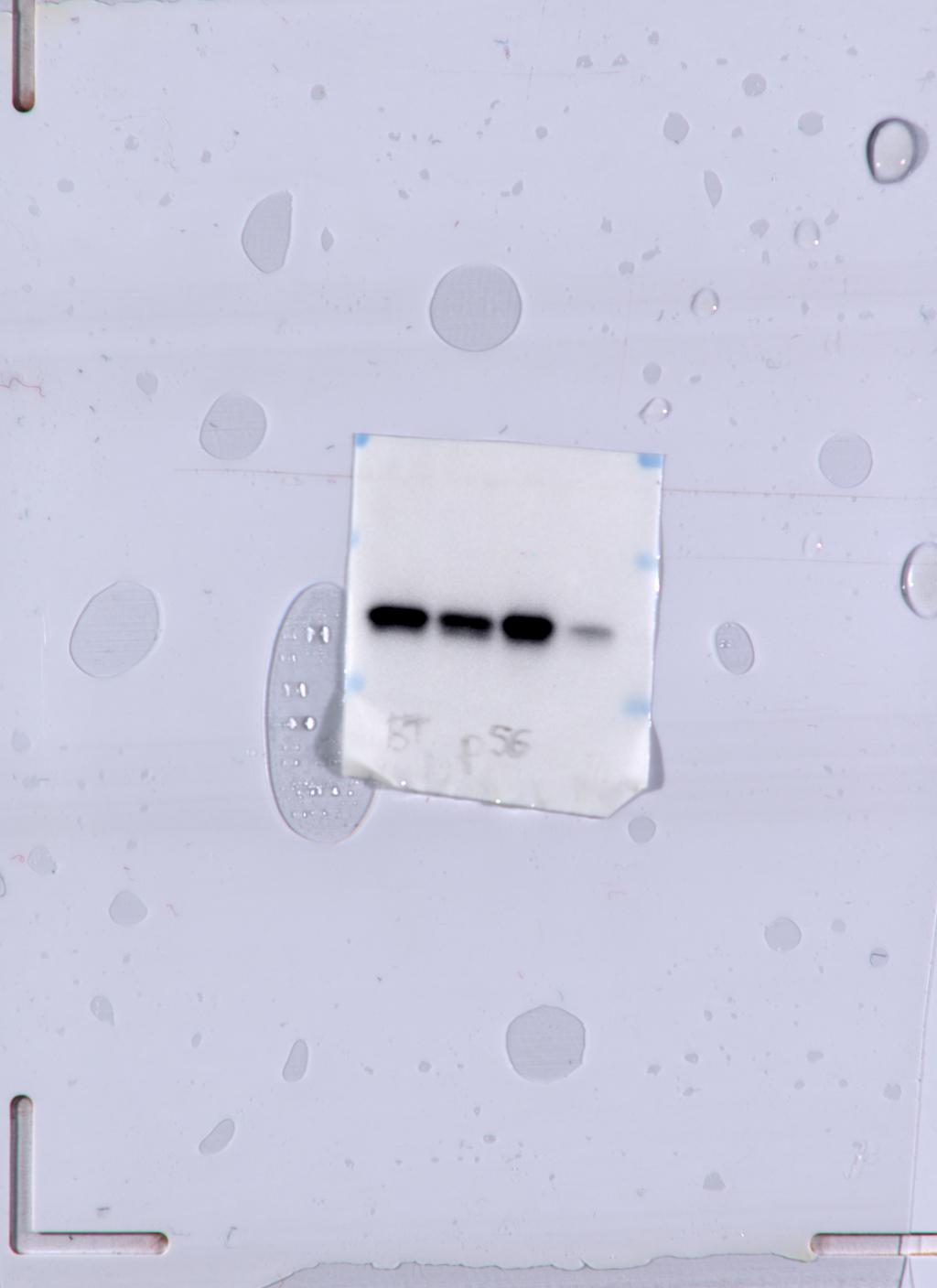

Supplement: Supplementary file 1 [file cancers-13-02778-s001.zip › Figure.S6/FigureS1/BT-474/BT474.pS6.jpg]

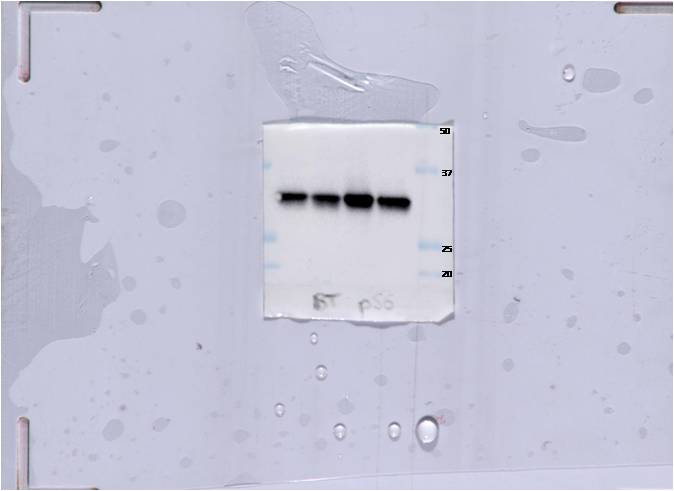

Supplement: Supplementary file 1 [file cancers-13-02778-s001.zip › Figure.S6/FigureS1/BT-474/BT474.rT3.pS6 MW.jpg]

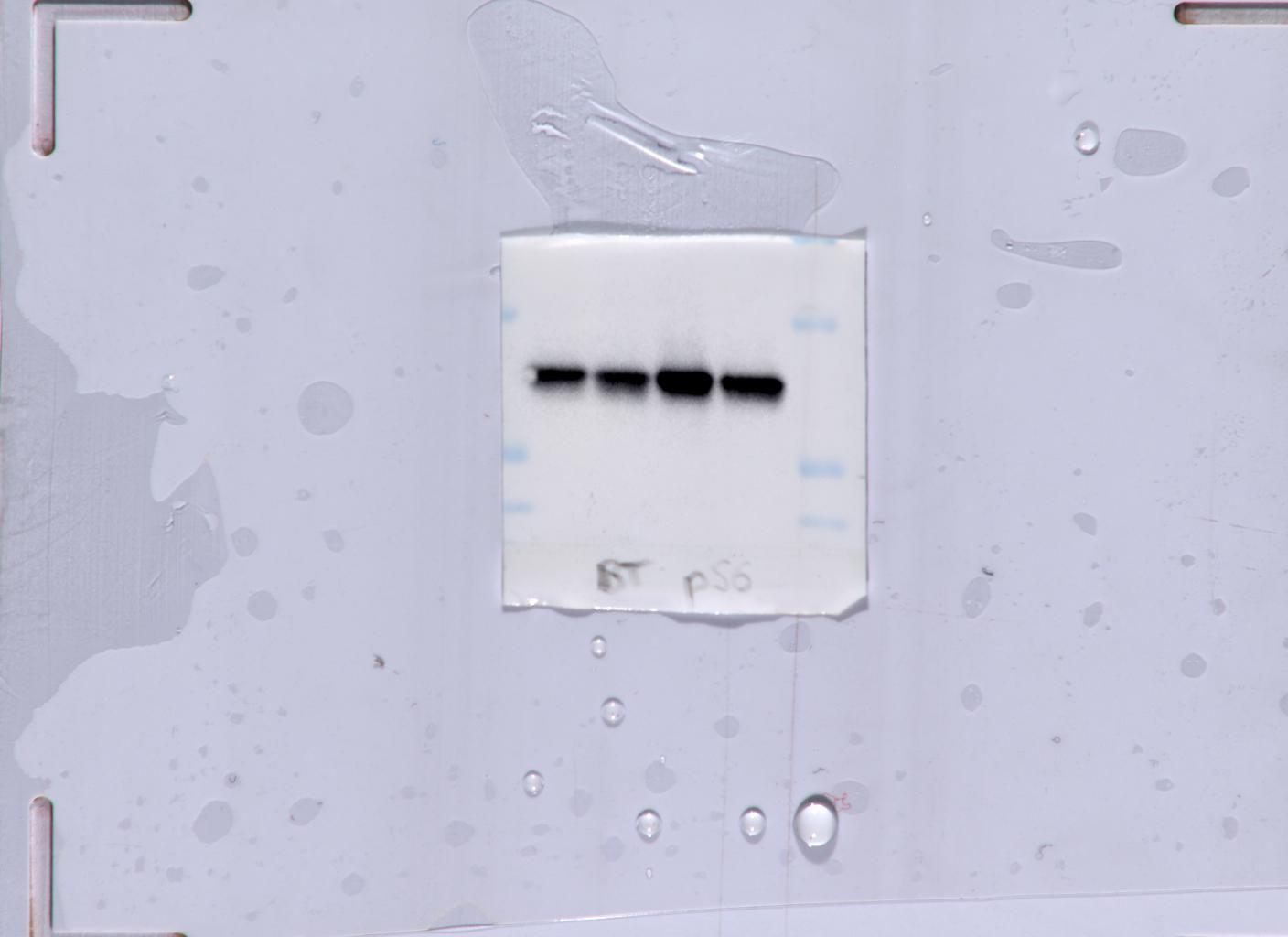

Supplement: Supplementary file 1 [file cancers-13-02778-s001.zip › Figure.S6/FigureS1/BT-474/BT474.rT3.pS6.jpg]

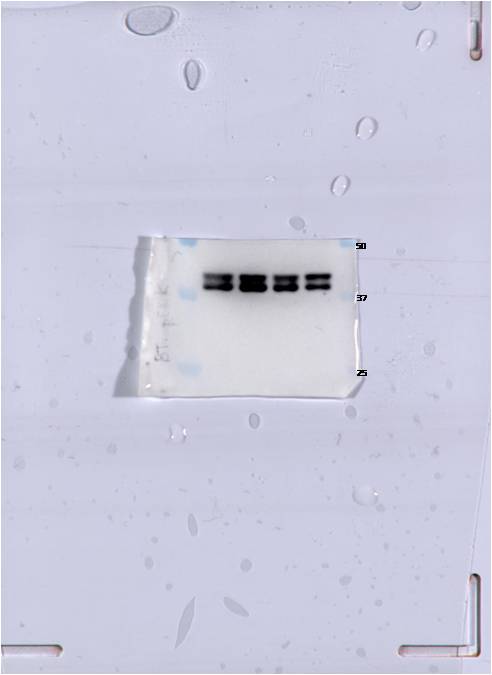

Supplement: Supplementary file 1 [file cancers-13-02778-s001.zip › Figure.S6/FigureS1/BT-474/ERK MW.jpg]

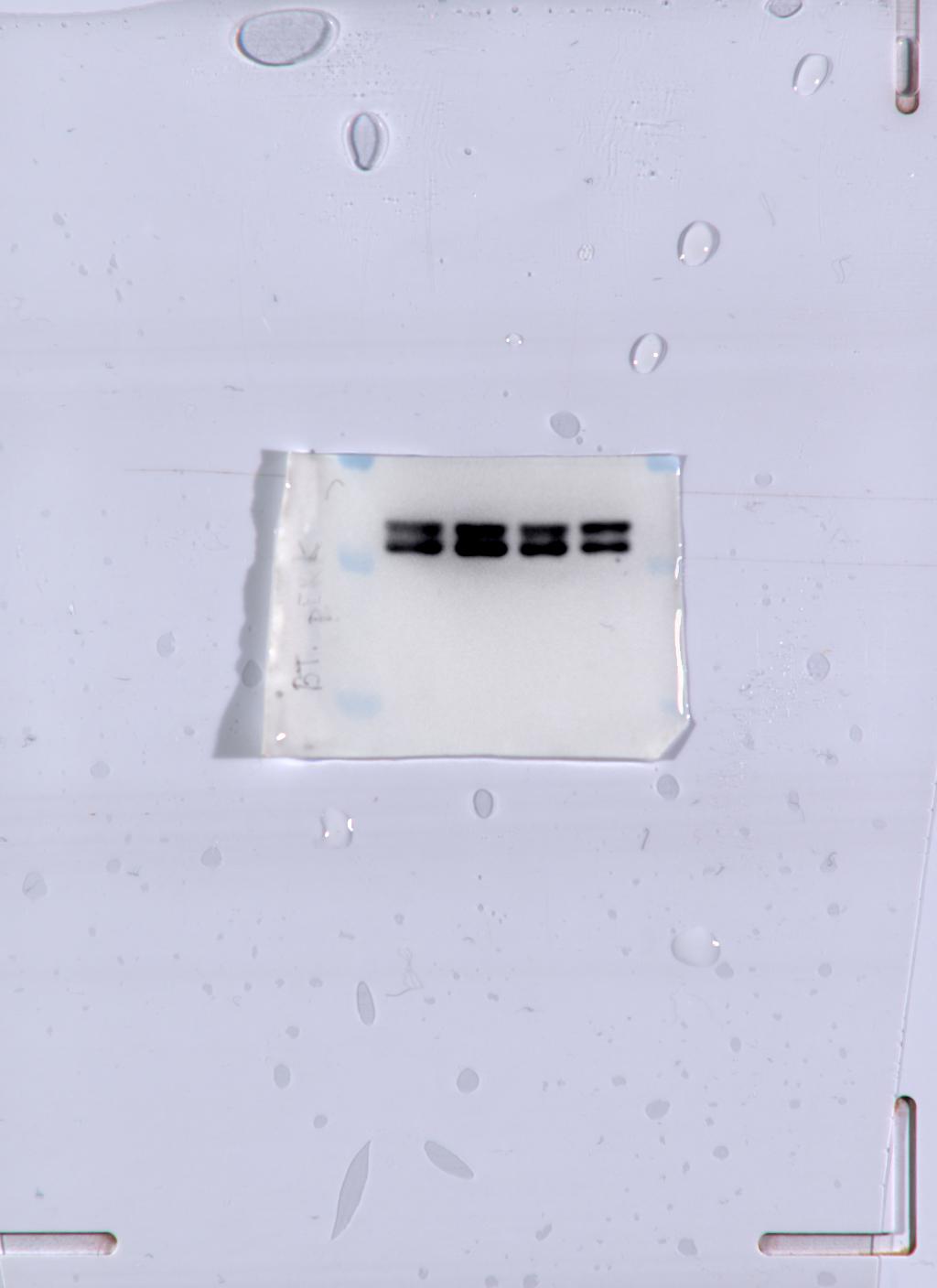

Supplement: Supplementary file 1 [file cancers-13-02778-s001.zip › Figure.S6/FigureS1/BT-474/ERK.jpg]

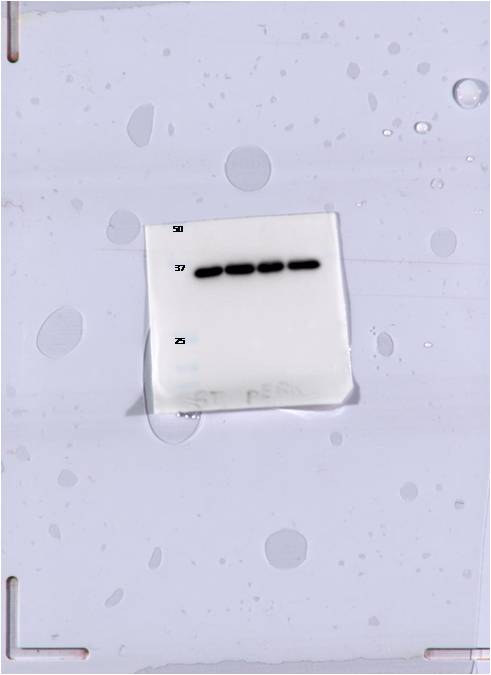

Supplement: Supplementary file 1 [file cancers-13-02778-s001.zip › Figure.S6/FigureS1/BT-474/GAPDH MW.jpg]

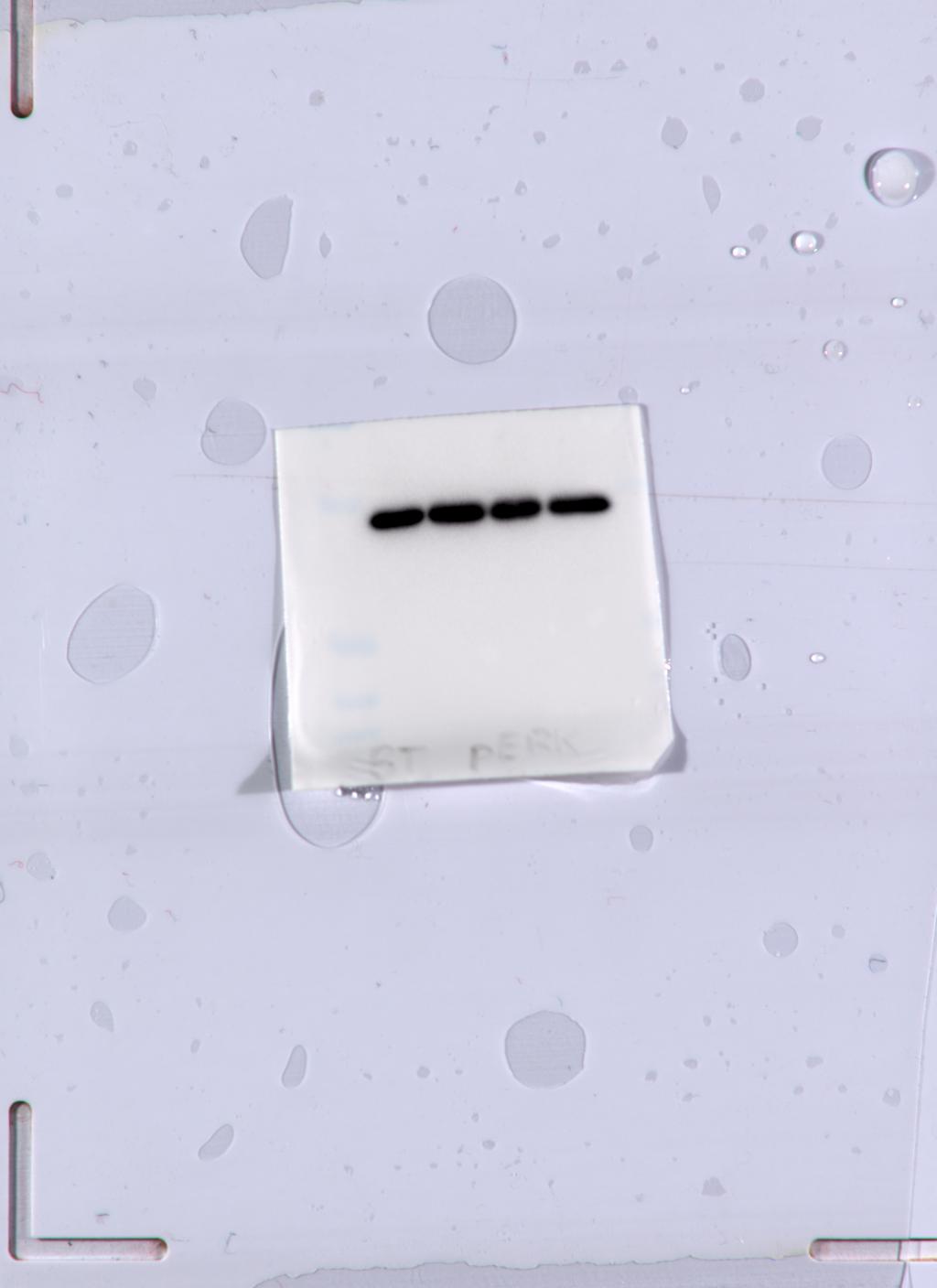

Supplement: Supplementary file 1 [file cancers-13-02778-s001.zip › Figure.S6/FigureS1/BT-474/GAPDH.jpg]

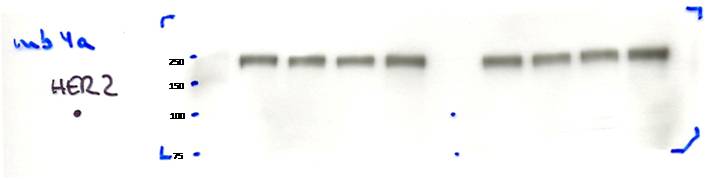

Supplement: Supplementary file 1 [file cancers-13-02778-s001.zip › Figure.S6/FigureS1/BT-474/HER2 MW.jpg]

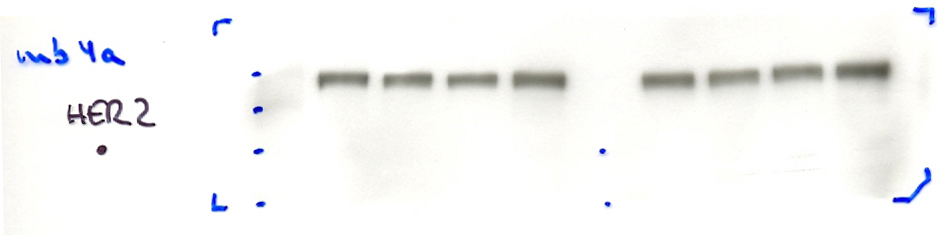

Supplement: Supplementary file 1 [file cancers-13-02778-s001.zip › Figure.S6/FigureS1/BT-474/HER2.jpg]

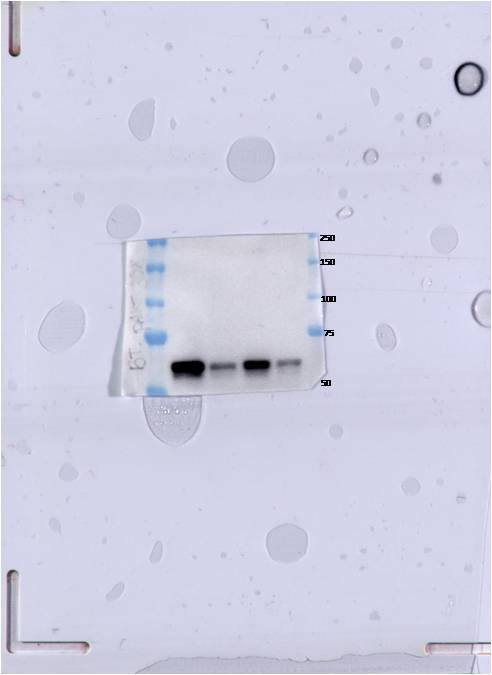

Supplement: Supplementary file 1 [file cancers-13-02778-s001.zip › Figure.S6/FigureS1/BT-474/pAKT 308 MW.jpg]

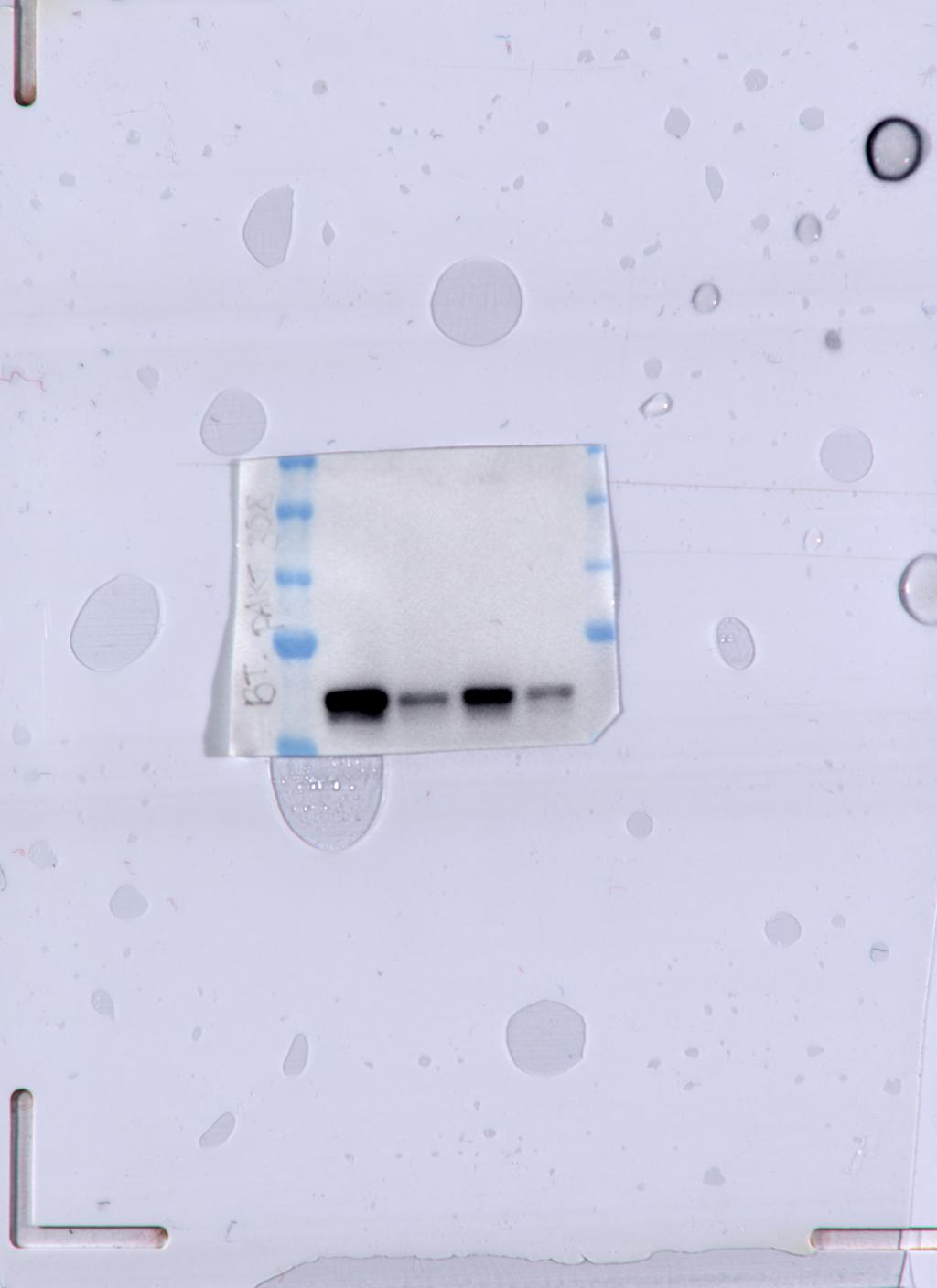

Supplement: Supplementary file 1 [file cancers-13-02778-s001.zip › Figure.S6/FigureS1/BT-474/pAKT 308.jpg]

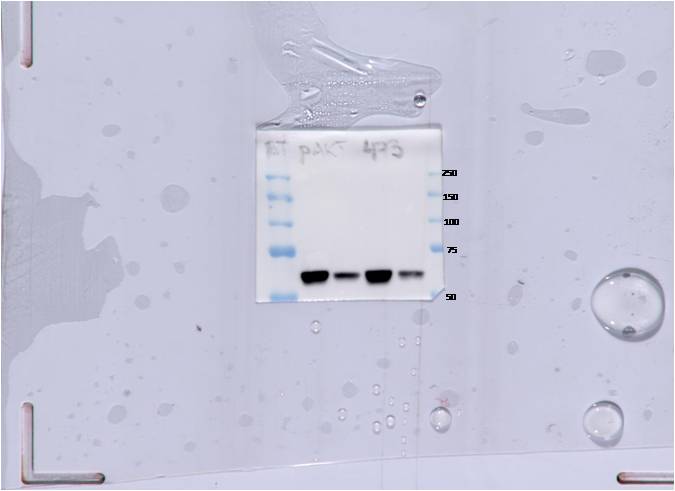

Supplement: Supplementary file 1 [file cancers-13-02778-s001.zip › Figure.S6/FigureS1/BT-474/pAKT 473 MW.jpg]

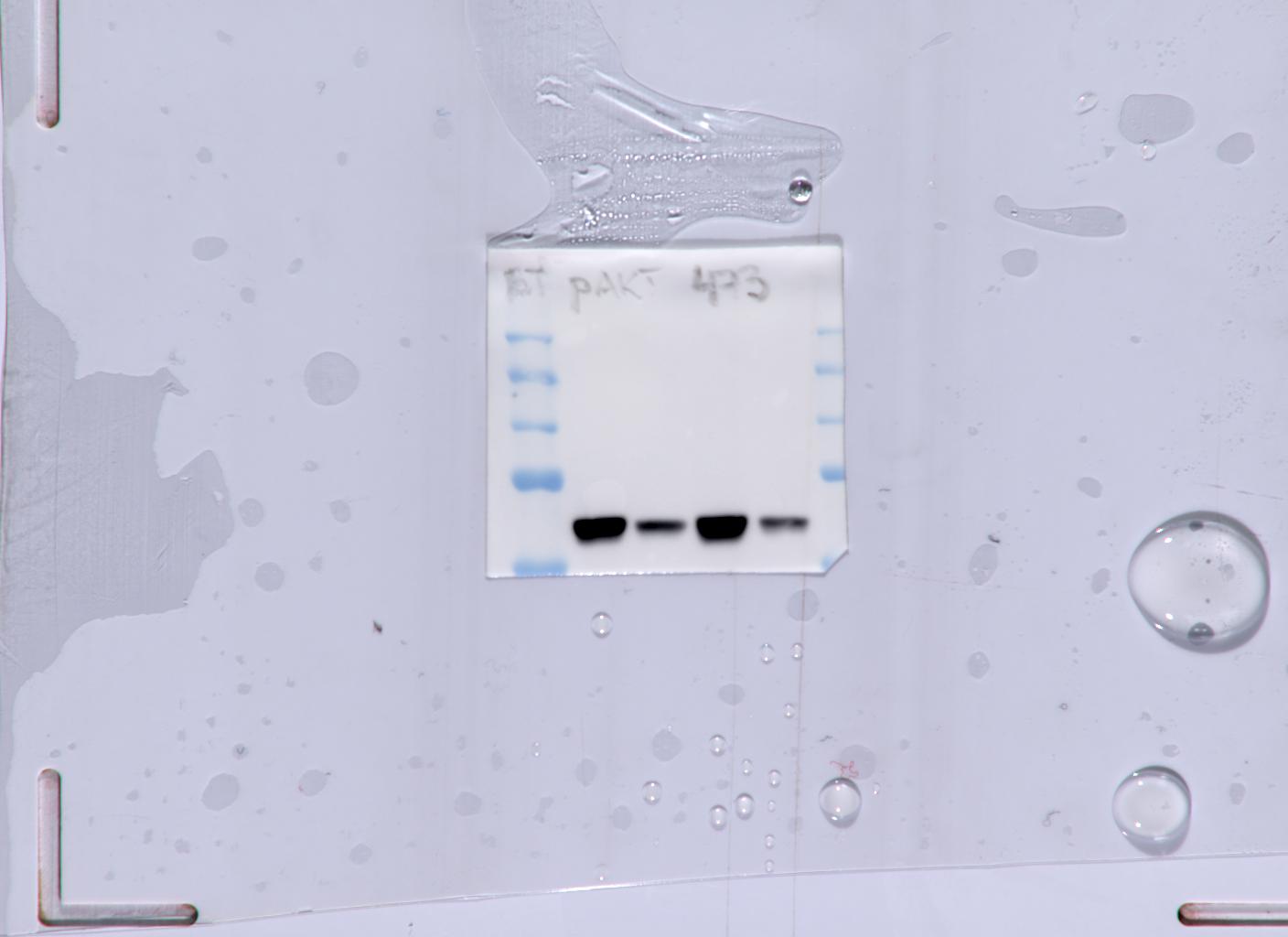

Supplement: Supplementary file 1 [file cancers-13-02778-s001.zip › Figure.S6/FigureS1/BT-474/pAKT 473.jpg]

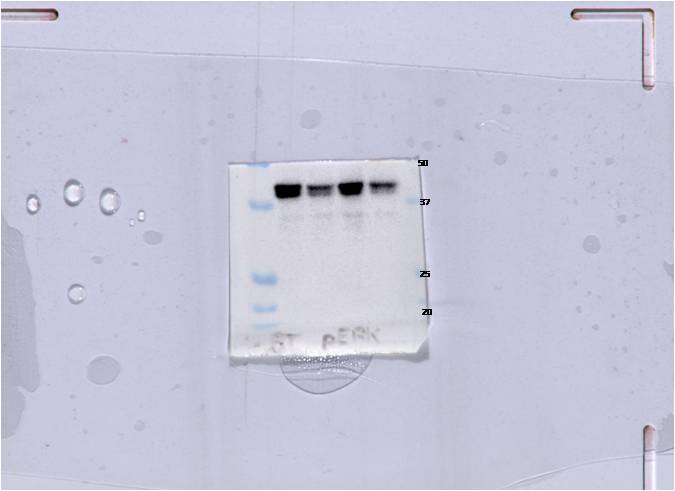

Supplement: Supplementary file 1 [file cancers-13-02778-s001.zip › Figure.S6/FigureS1/BT-474/pERK MW.jpg]

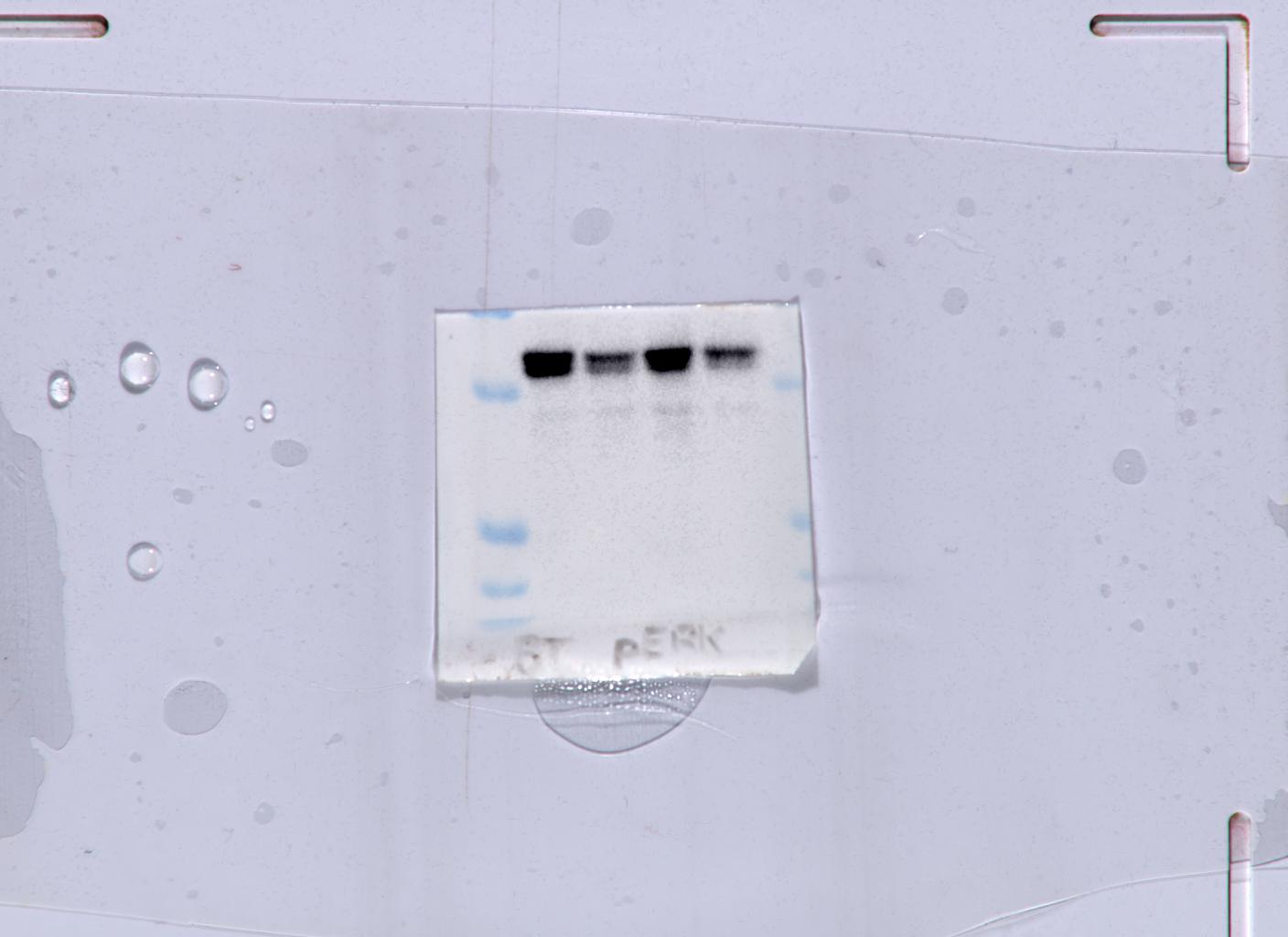

Supplement: Supplementary file 1 [file cancers-13-02778-s001.zip › Figure.S6/FigureS1/BT-474/pERK.jpg]

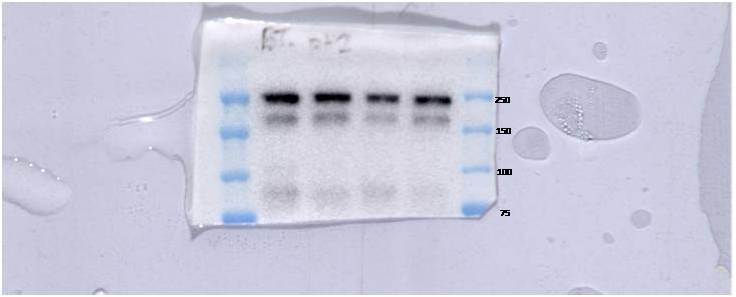

Supplement: Supplementary file 1 [file cancers-13-02778-s001.zip › Figure.S6/FigureS1/BT-474/pHER2 MW.jpg]

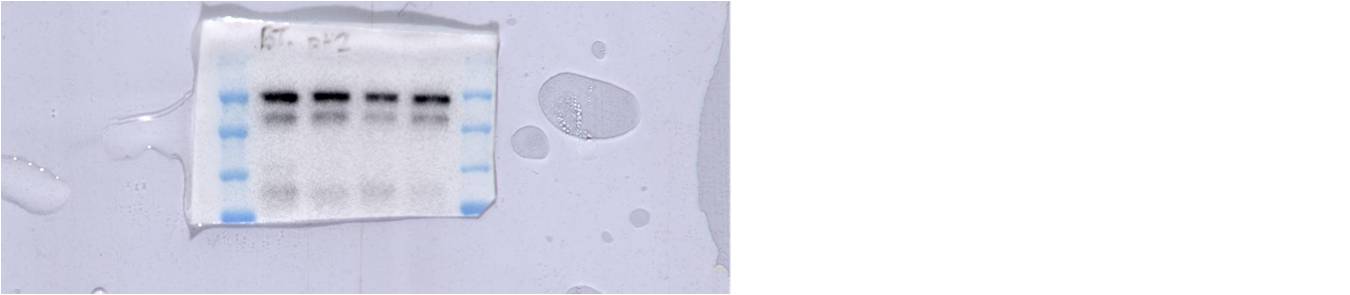

Supplement: Supplementary file 1 [file cancers-13-02778-s001.zip › Figure.S6/FigureS1/BT-474/pHER2.jpg]

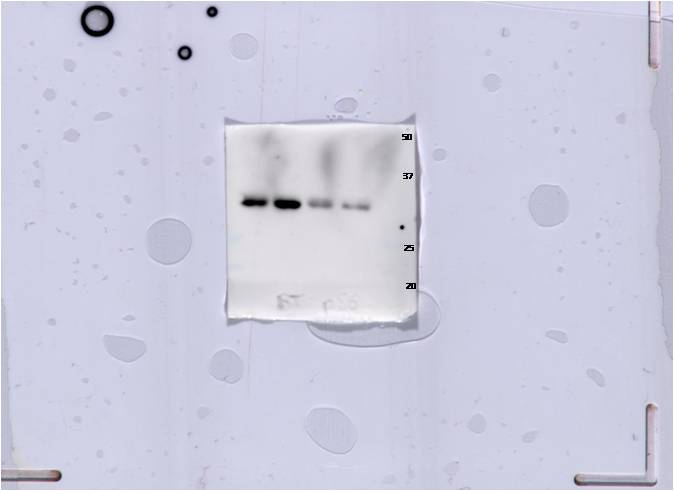

Supplement: Supplementary file 1 [file cancers-13-02778-s001.zip › Figure.S6/FigureS1/BT-474/S6 MW.jpg]

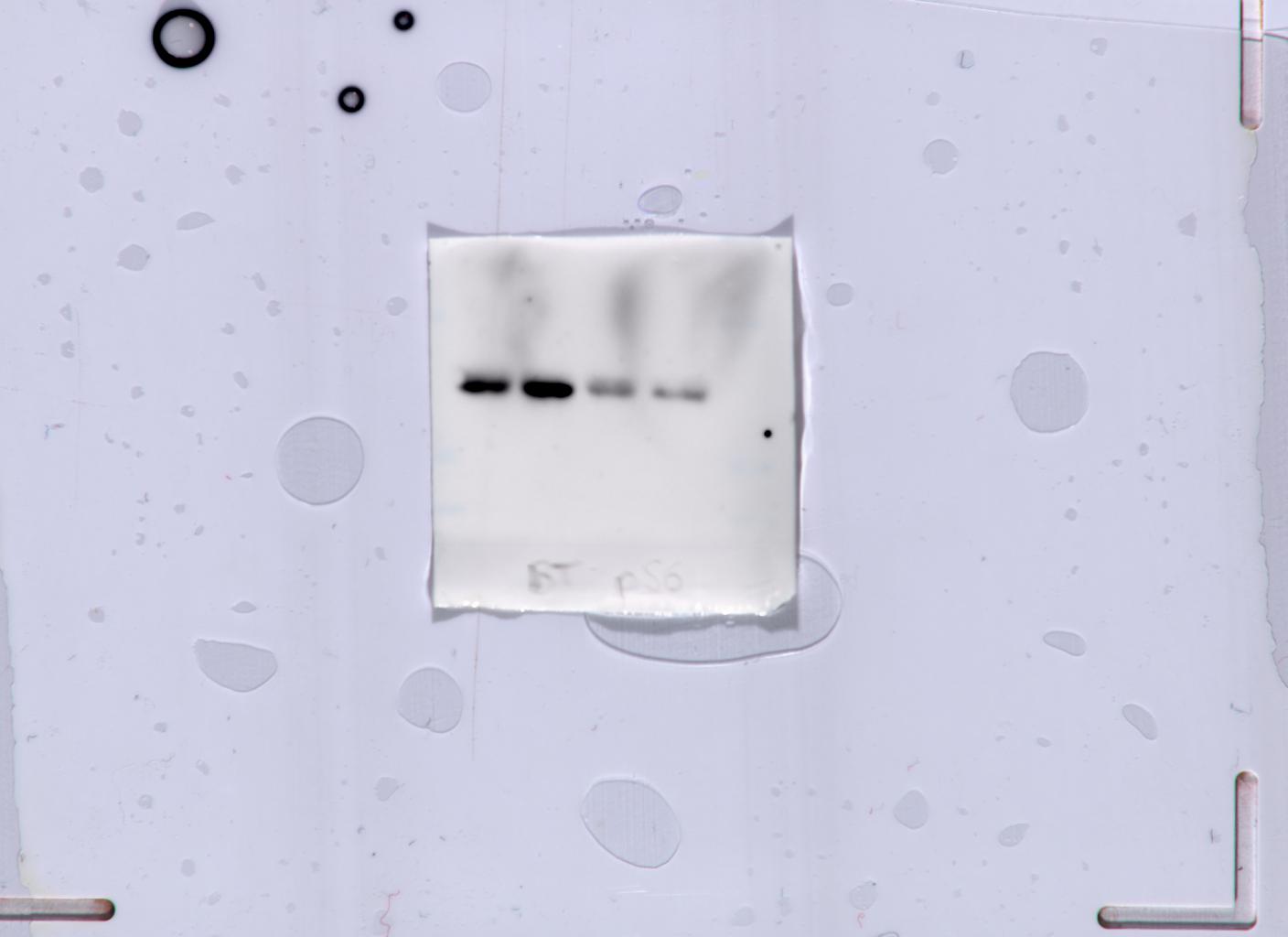

Supplement: Supplementary file 1 [file cancers-13-02778-s001.zip › Figure.S6/FigureS1/BT-474/S6.jpg]

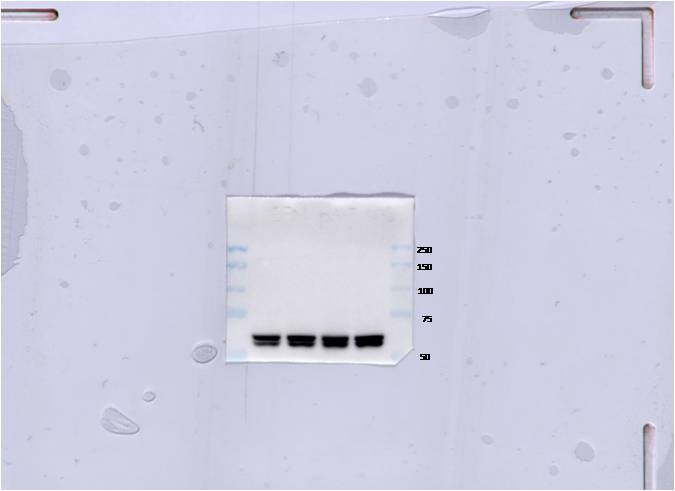

Supplement: Supplementary file 1 [file cancers-13-02778-s001.zip › Figure.S6/FigureS1/EFM-192A/AKT MW.jpg]

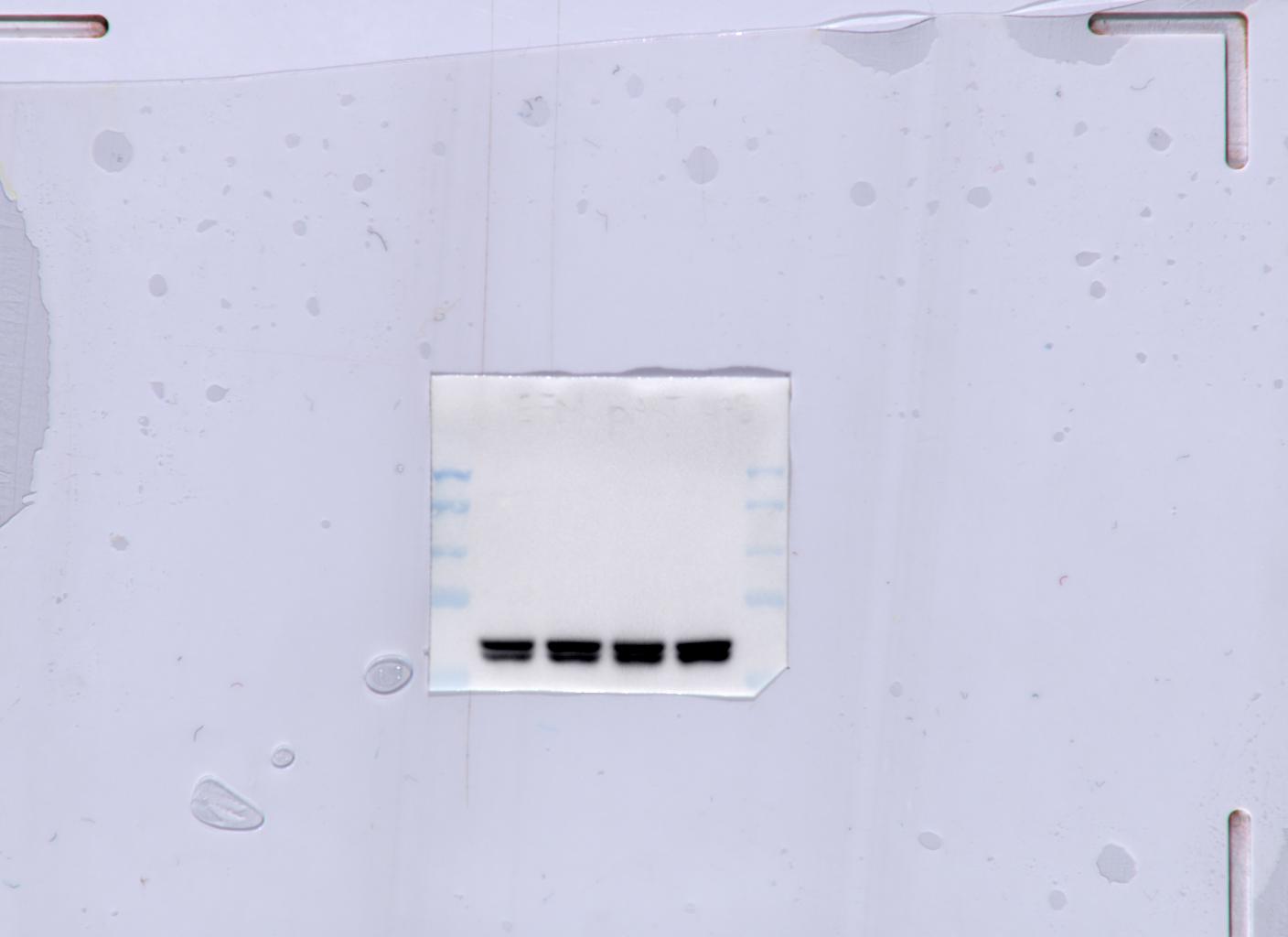

Supplement: Supplementary file 1 [file cancers-13-02778-s001.zip › Figure.S6/FigureS1/EFM-192A/AKT.jpg]

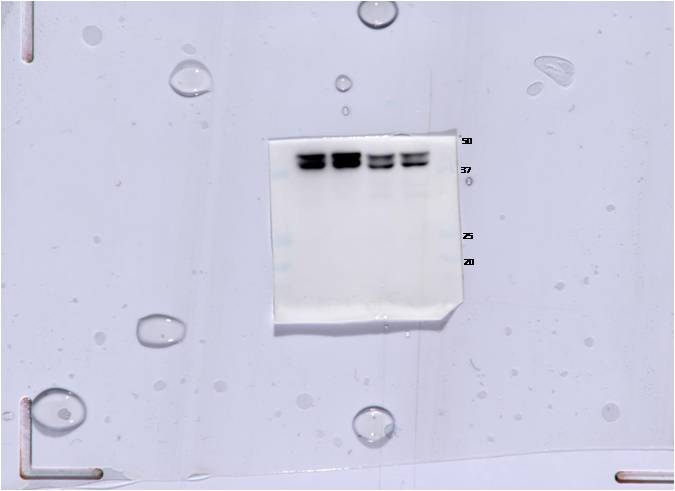

Supplement: Supplementary file 1 [file cancers-13-02778-s001.zip › Figure.S6/FigureS1/EFM-192A/ERK MW.jpg]

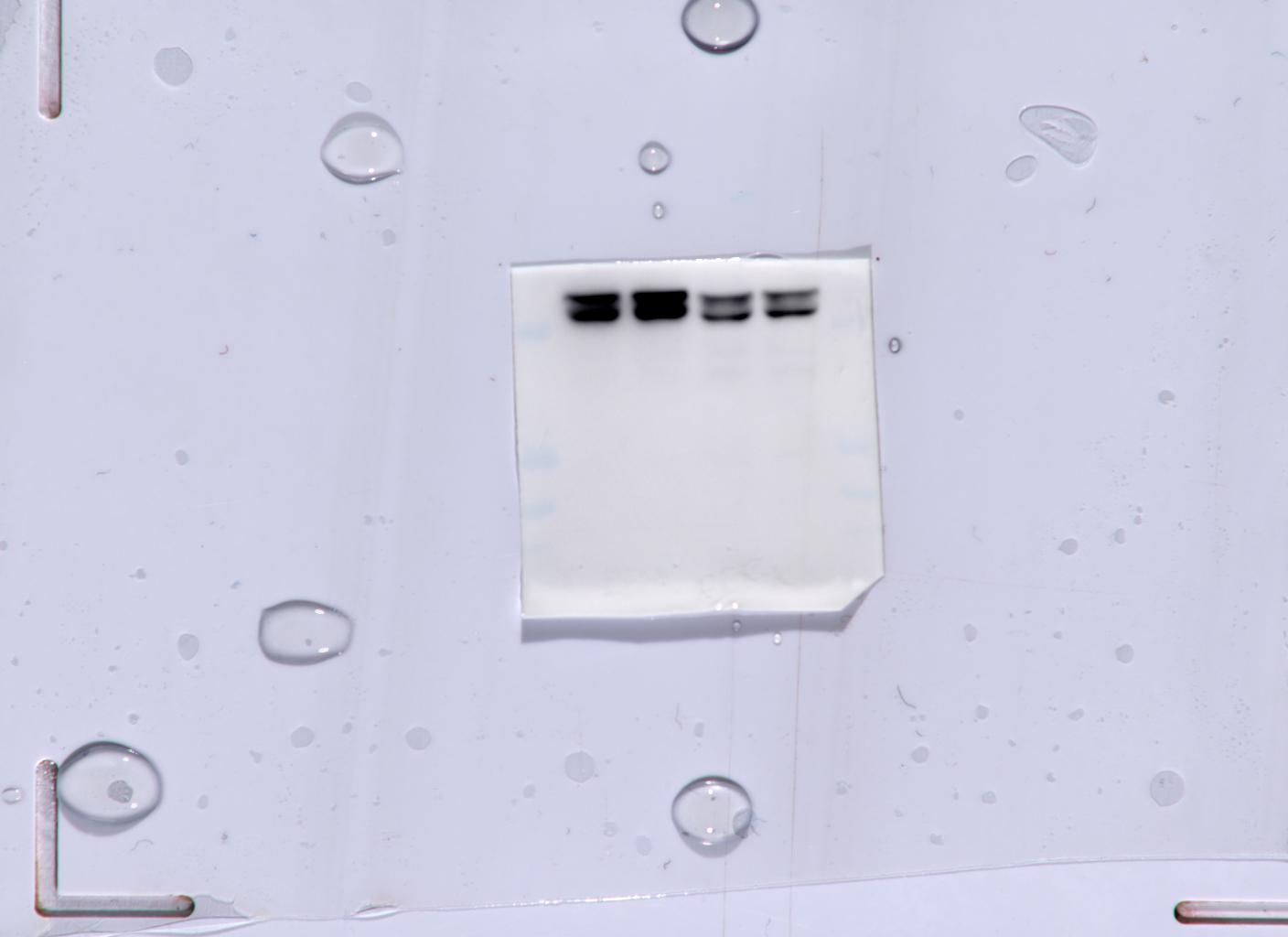

Supplement: Supplementary file 1 [file cancers-13-02778-s001.zip › Figure.S6/FigureS1/EFM-192A/ERK.jpg]

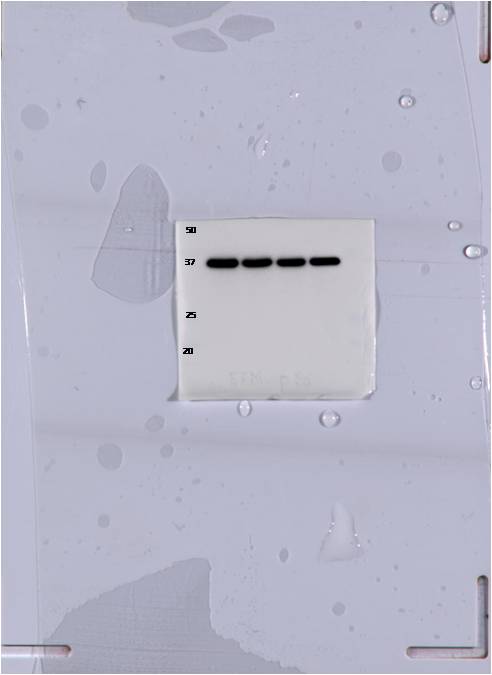

Supplement: Supplementary file 1 [file cancers-13-02778-s001.zip › Figure.S6/FigureS1/EFM-192A/GAPDH MW.jpg]

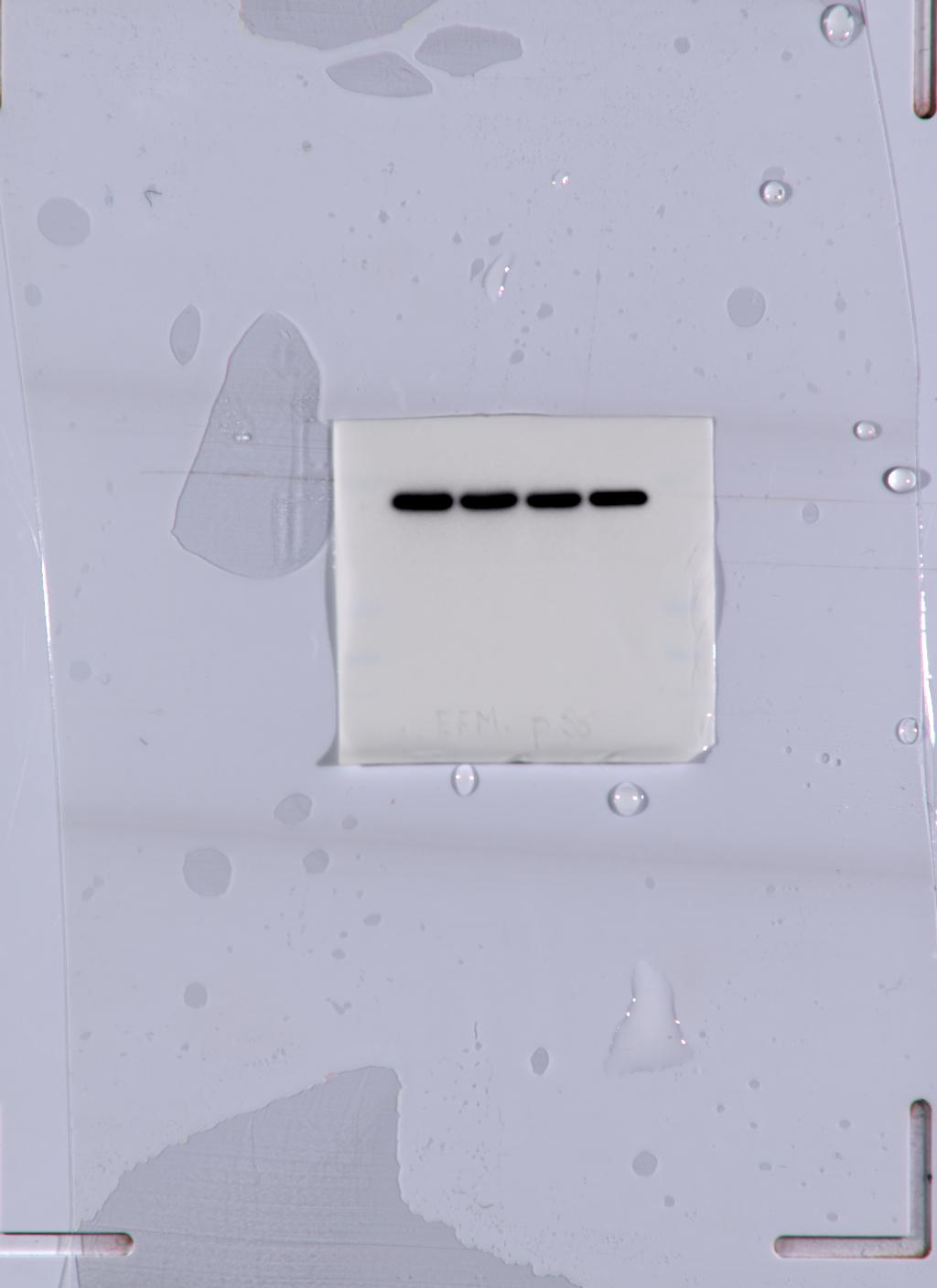

Supplement: Supplementary file 1 [file cancers-13-02778-s001.zip › Figure.S6/FigureS1/EFM-192A/GAPDH.jpg]

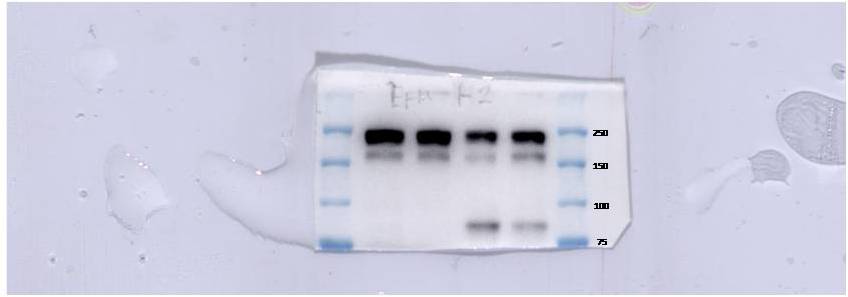

Supplement: Supplementary file 1 [file cancers-13-02778-s001.zip › Figure.S6/FigureS1/EFM-192A/HER2 MW.jpg]

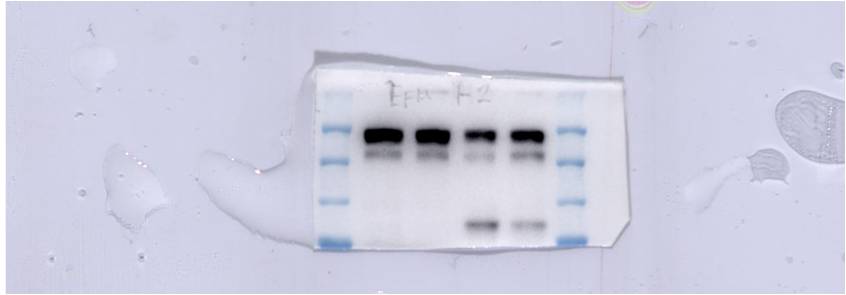

Supplement: Supplementary file 1 [file cancers-13-02778-s001.zip › Figure.S6/FigureS1/EFM-192A/HER2.jpg]

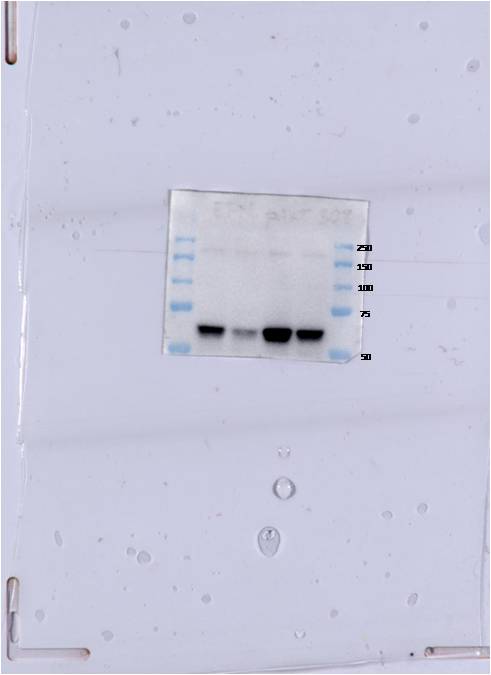

Supplement: Supplementary file 1 [file cancers-13-02778-s001.zip › Figure.S6/FigureS1/EFM-192A/pAKT 308 MW.jpg]

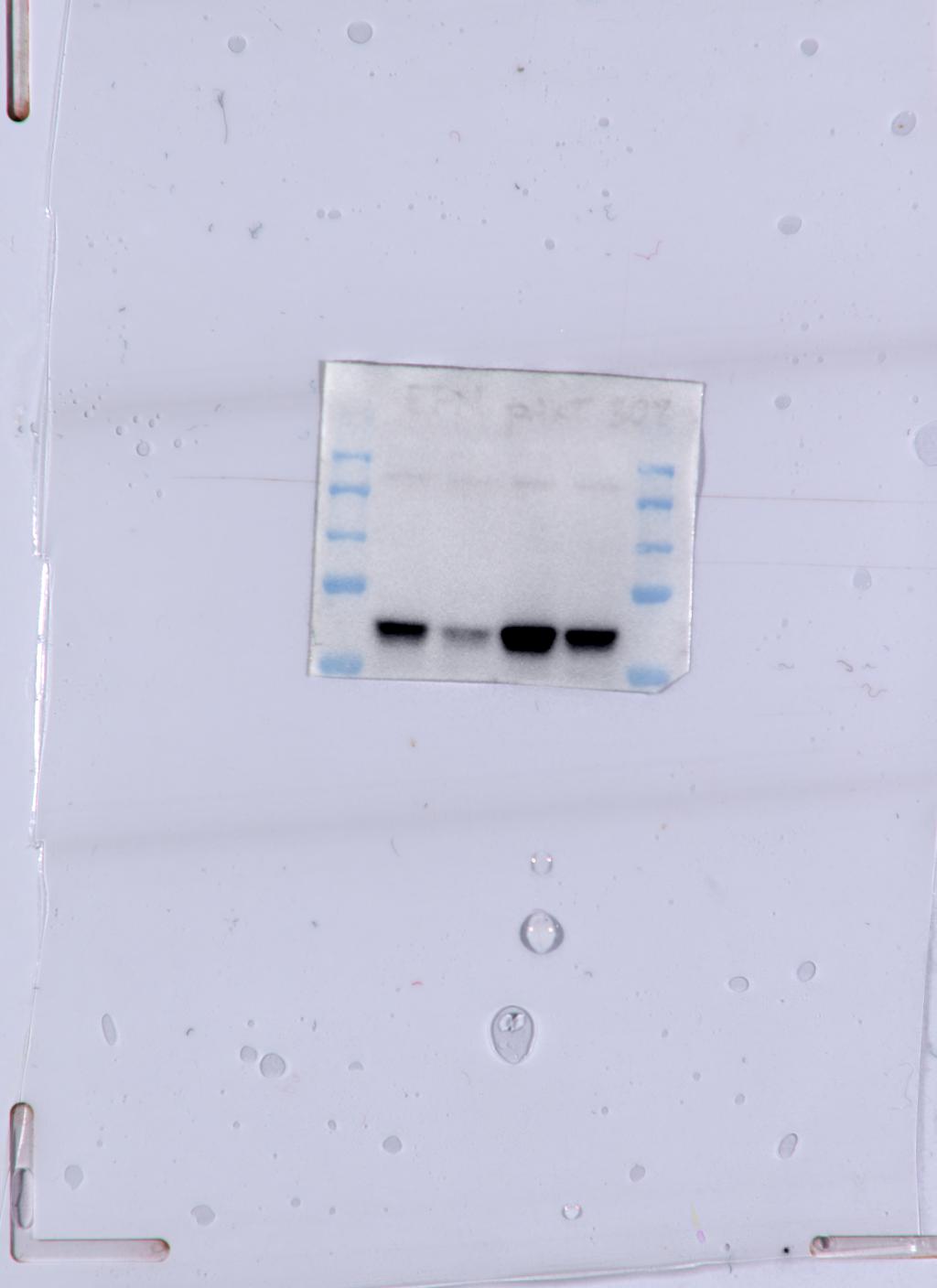

Supplement: Supplementary file 1 [file cancers-13-02778-s001.zip › Figure.S6/FigureS1/EFM-192A/pAKT 308.jpg]

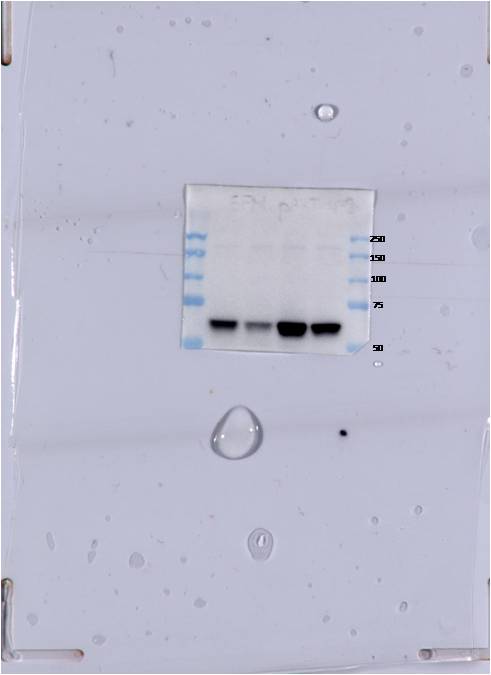

Supplement: Supplementary file 1 [file cancers-13-02778-s001.zip › Figure.S6/FigureS1/EFM-192A/pAKT 473 MW.jpg]

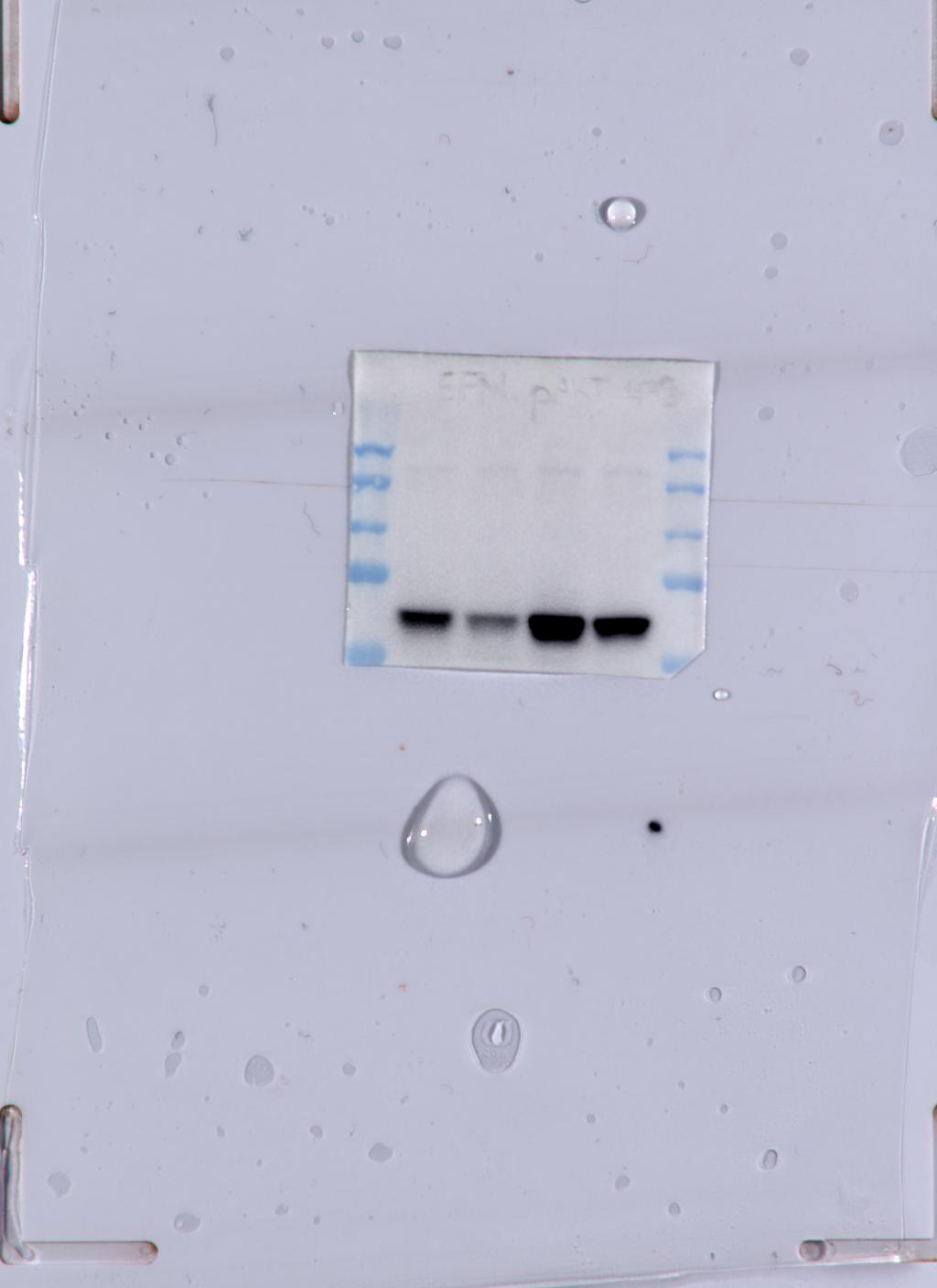

Supplement: Supplementary file 1 [file cancers-13-02778-s001.zip › Figure.S6/FigureS1/EFM-192A/pAKT 473.jpg]

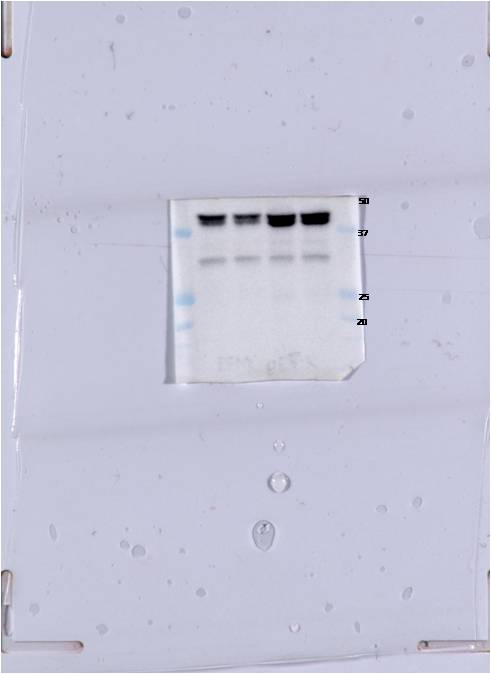

Supplement: Supplementary file 1 [file cancers-13-02778-s001.zip › Figure.S6/FigureS1/EFM-192A/pERK MW.jpg]

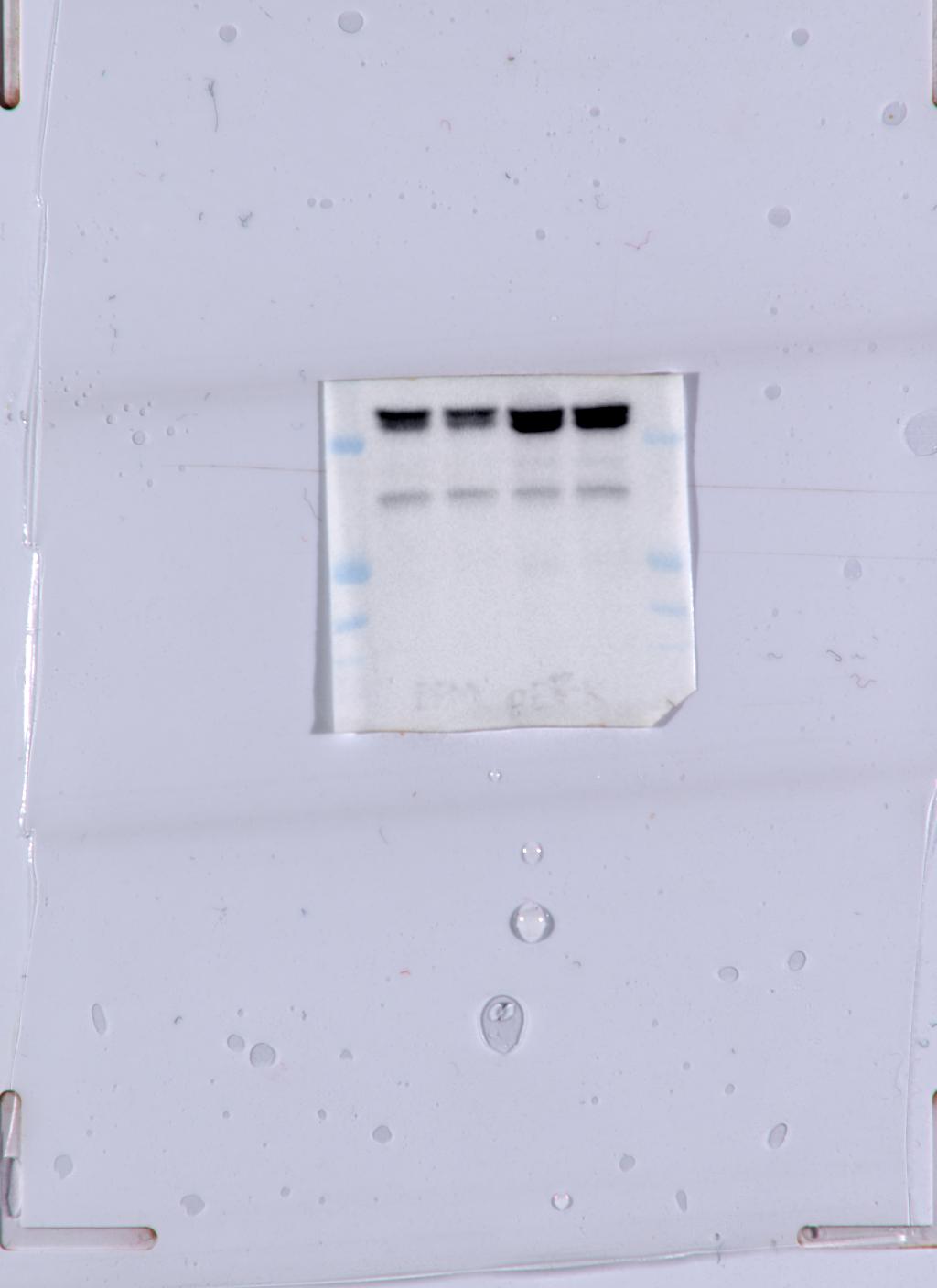

Supplement: Supplementary file 1 [file cancers-13-02778-s001.zip › Figure.S6/FigureS1/EFM-192A/pERK.jpg]

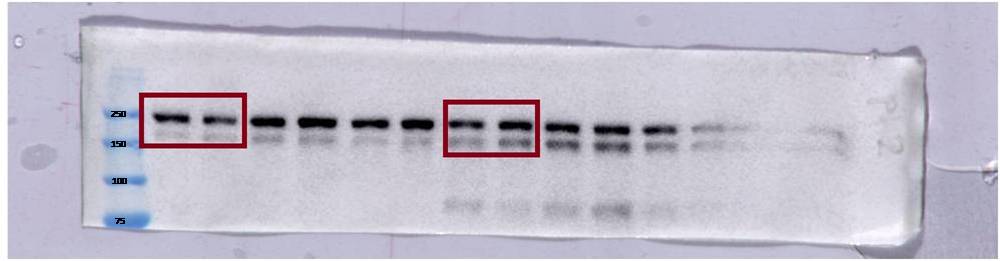

Supplement: Supplementary file 1 [file cancers-13-02778-s001.zip › Figure.S6/FigureS1/EFM-192A/pHER2 MW.jpg]

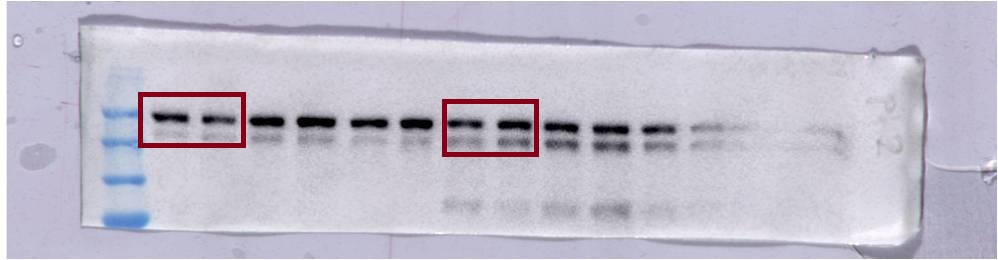

Supplement: Supplementary file 1 [file cancers-13-02778-s001.zip › Figure.S6/FigureS1/EFM-192A/pHER2.jpg]

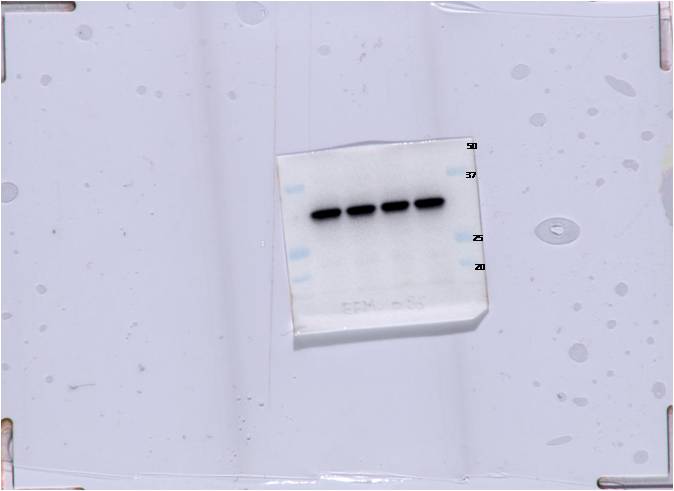

Supplement: Supplementary file 1 [file cancers-13-02778-s001.zip › Figure.S6/FigureS1/EFM-192A/pS6 MW.jpg]

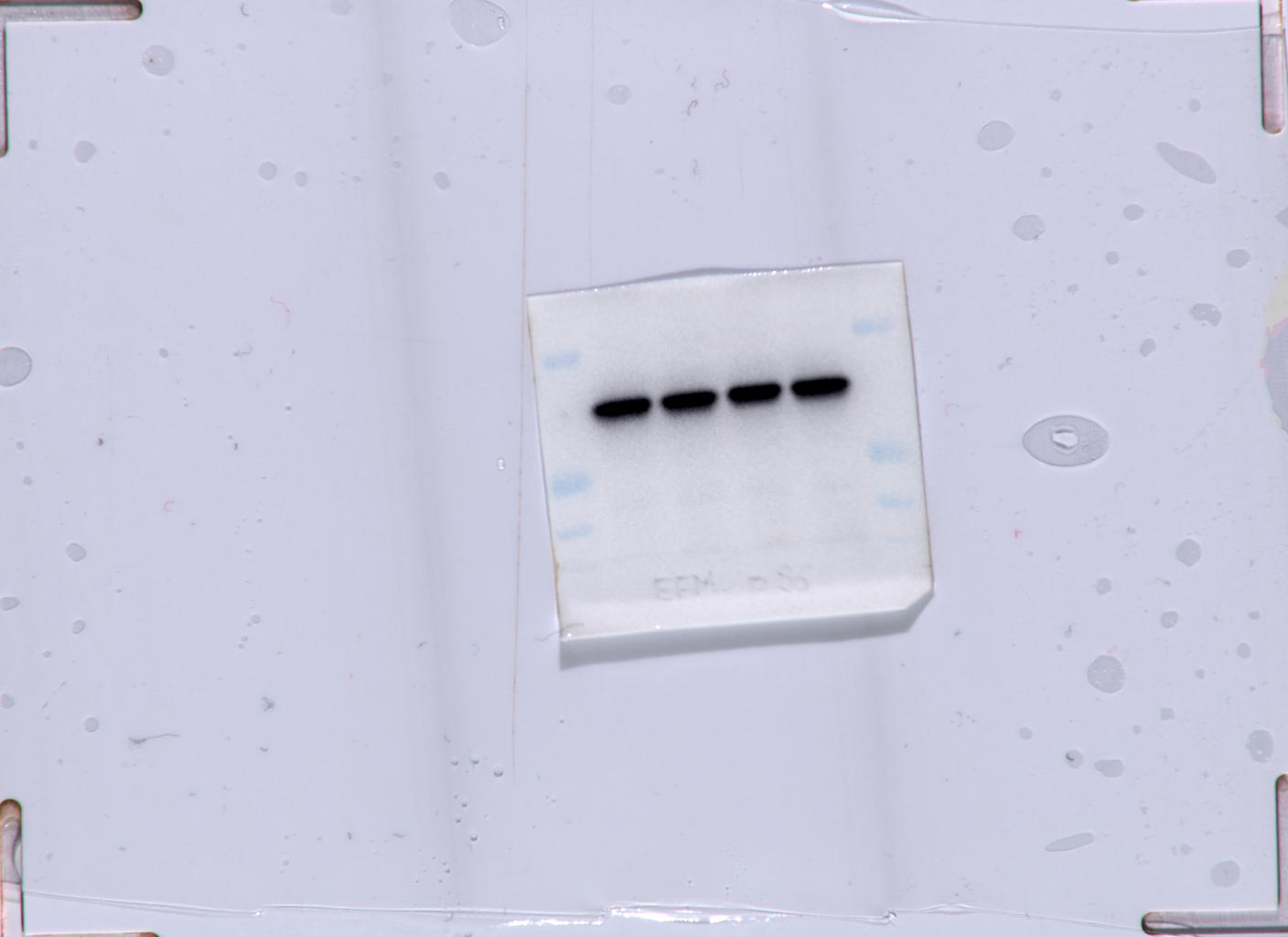

Supplement: Supplementary file 1 [file cancers-13-02778-s001.zip › Figure.S6/FigureS1/EFM-192A/pS6.jpg]

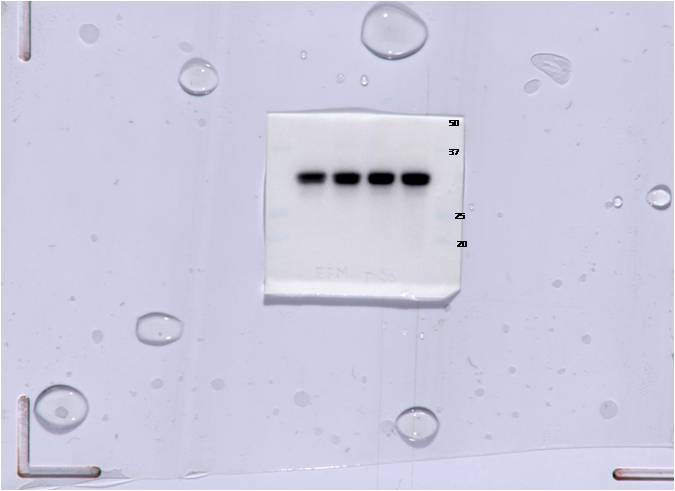

Supplement: Supplementary file 1 [file cancers-13-02778-s001.zip › Figure.S6/FigureS1/EFM-192A/S6 MW.jpg]

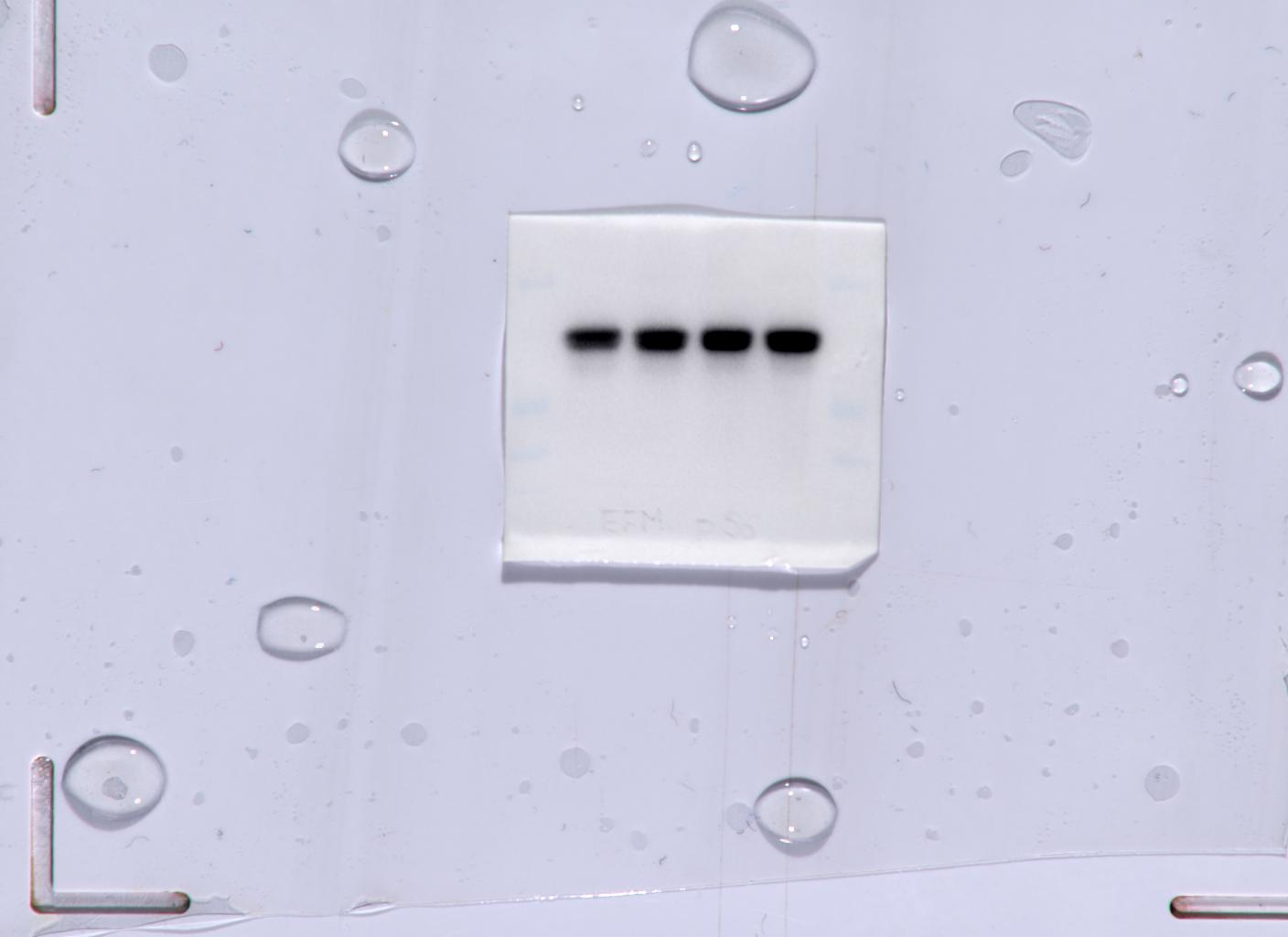

Supplement: Supplementary file 1 [file cancers-13-02778-s001.zip › Figure.S6/FigureS1/EFM-192A/S6.jpg]

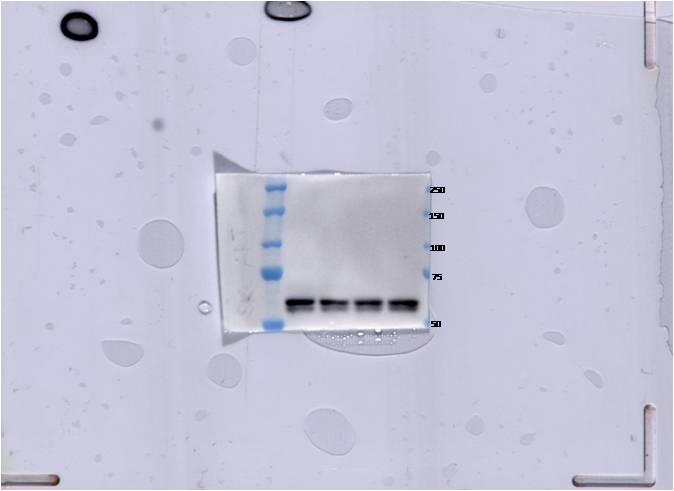

Supplement: Supplementary file 1 [file cancers-13-02778-s001.zip › Figure.S6/FigureS1/SK-BR-3/AKT MW.jpg]

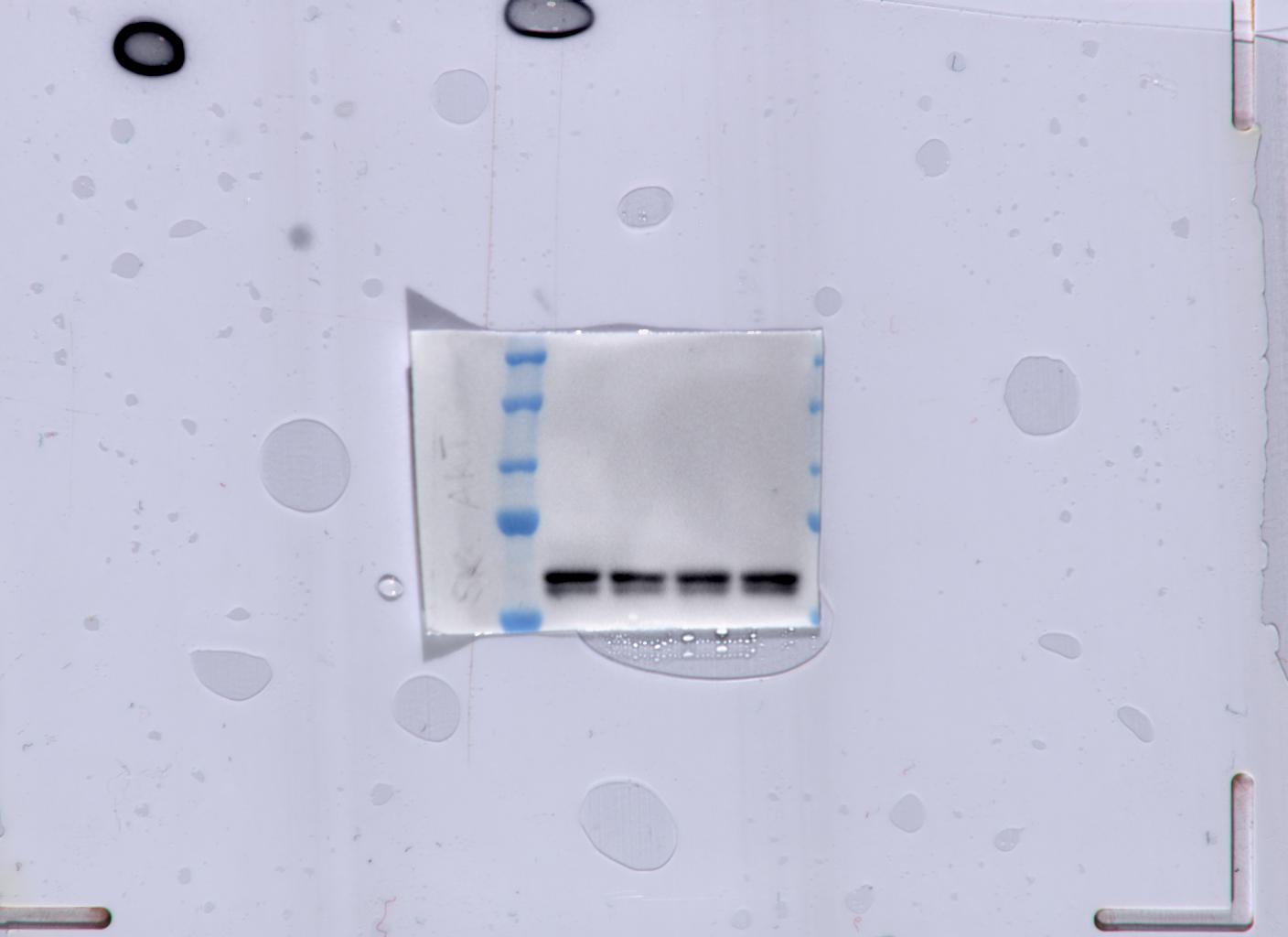

Supplement: Supplementary file 1 [file cancers-13-02778-s001.zip › Figure.S6/FigureS1/SK-BR-3/AKT.jpg]

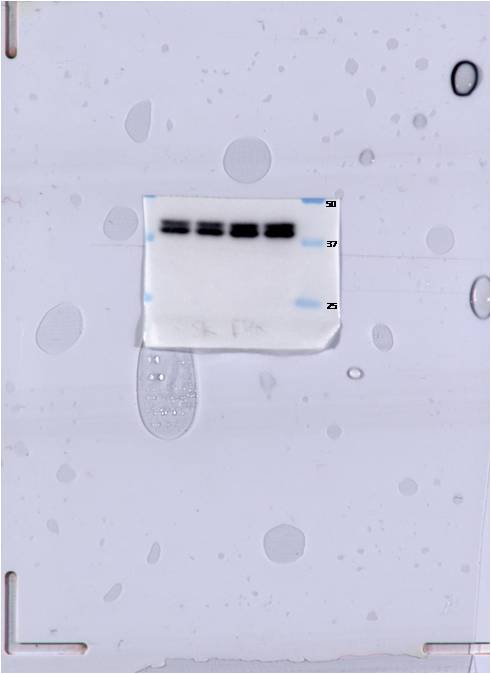

Supplement: Supplementary file 1 [file cancers-13-02778-s001.zip › Figure.S6/FigureS1/SK-BR-3/ERK MW.jpg]

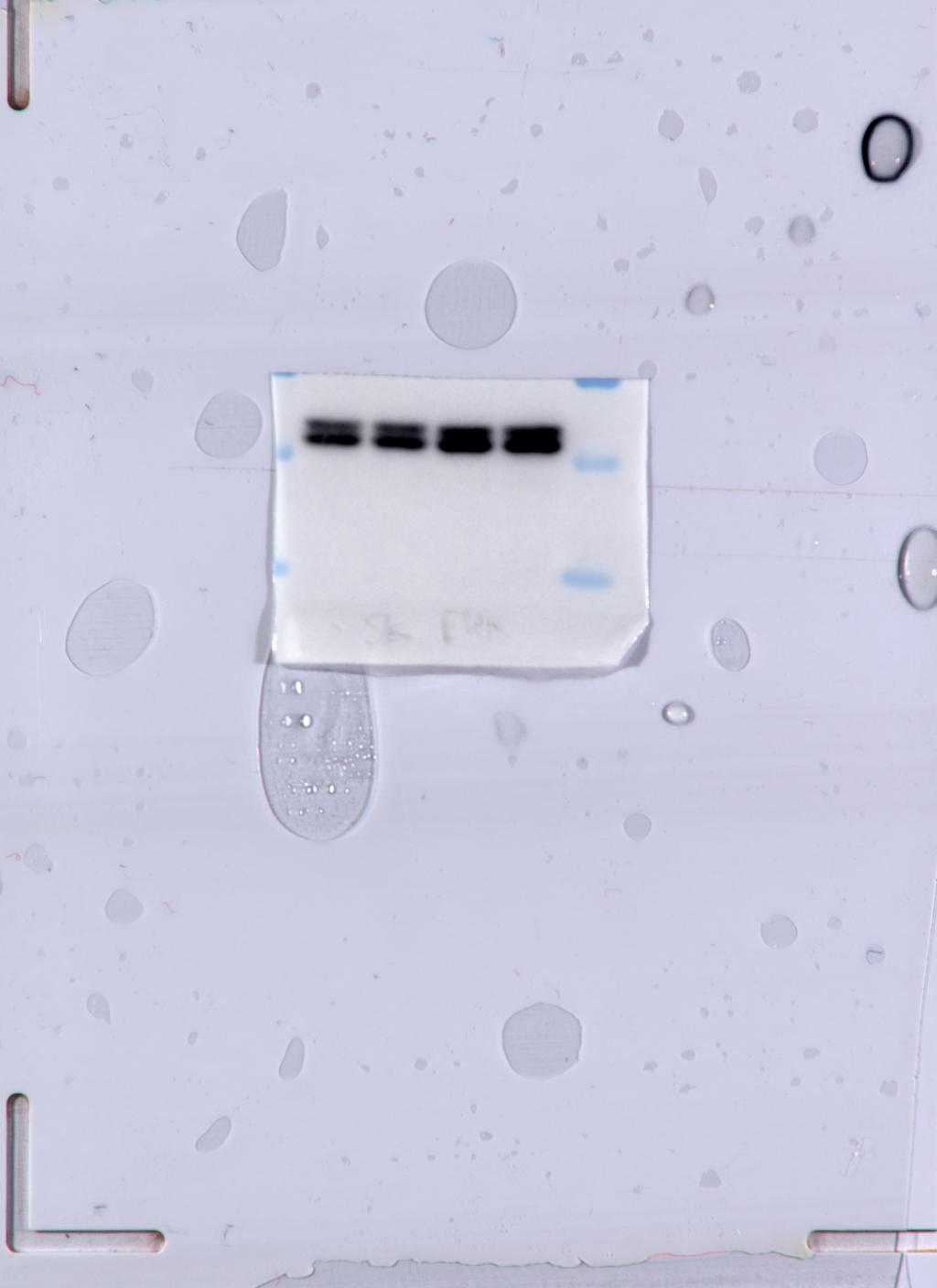

Supplement: Supplementary file 1 [file cancers-13-02778-s001.zip › Figure.S6/FigureS1/SK-BR-3/ERK.jpg]

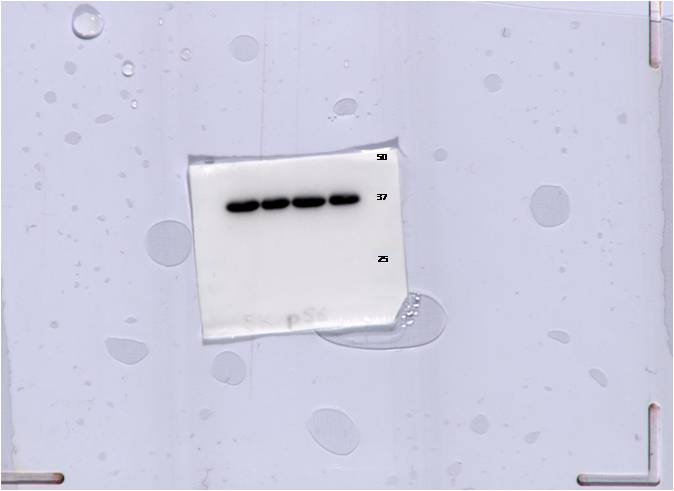

Supplement: Supplementary file 1 [file cancers-13-02778-s001.zip › Figure.S6/FigureS1/SK-BR-3/GAPDH MW.jpg]

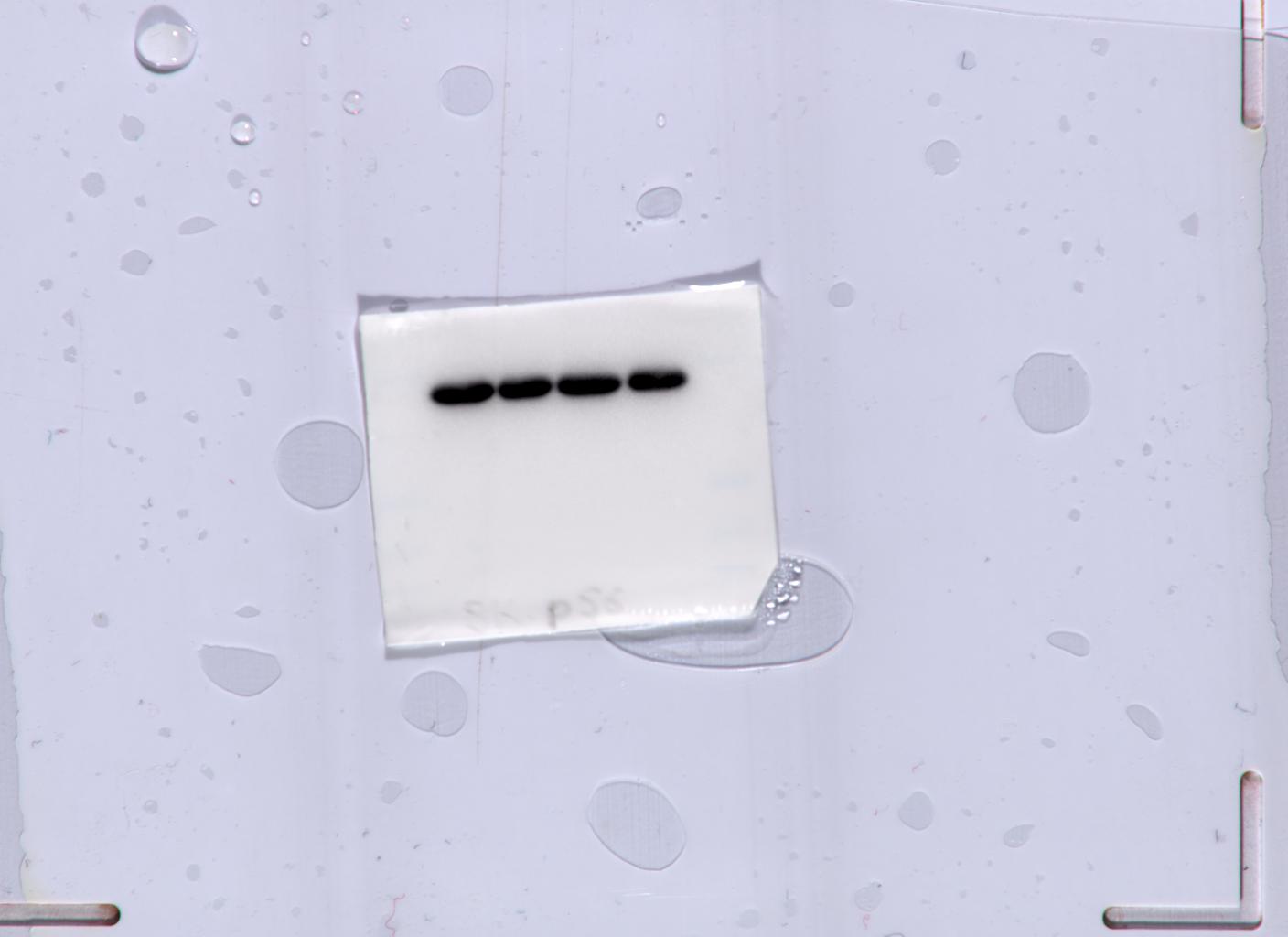

Supplement: Supplementary file 1 [file cancers-13-02778-s001.zip › Figure.S6/FigureS1/SK-BR-3/GAPDH.jpg]

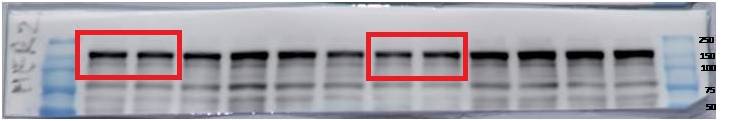

Supplement: Supplementary file 1 [file cancers-13-02778-s001.zip › Figure.S6/FigureS1/SK-BR-3/HER2 MW.jpg]

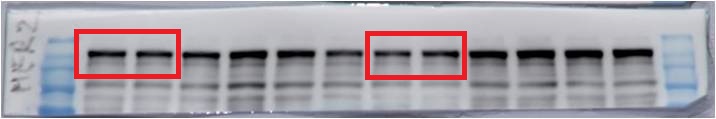

Supplement: Supplementary file 1 [file cancers-13-02778-s001.zip › Figure.S6/FigureS1/SK-BR-3/HER2.jpg]

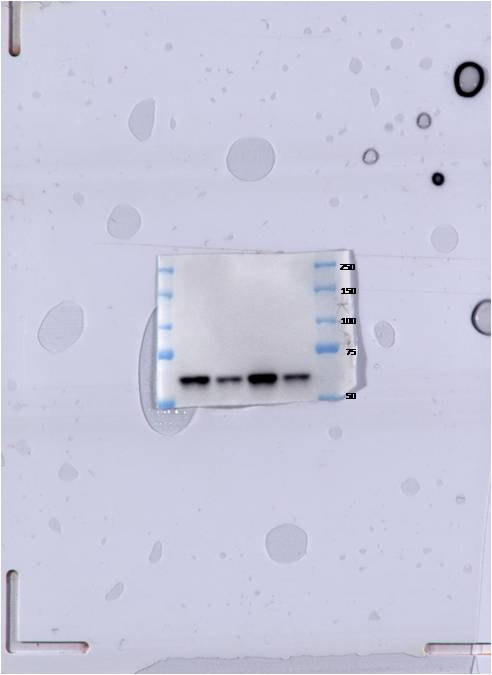

Supplement: Supplementary file 1 [file cancers-13-02778-s001.zip › Figure.S6/FigureS1/SK-BR-3/pAKT 308 MW.jpg]

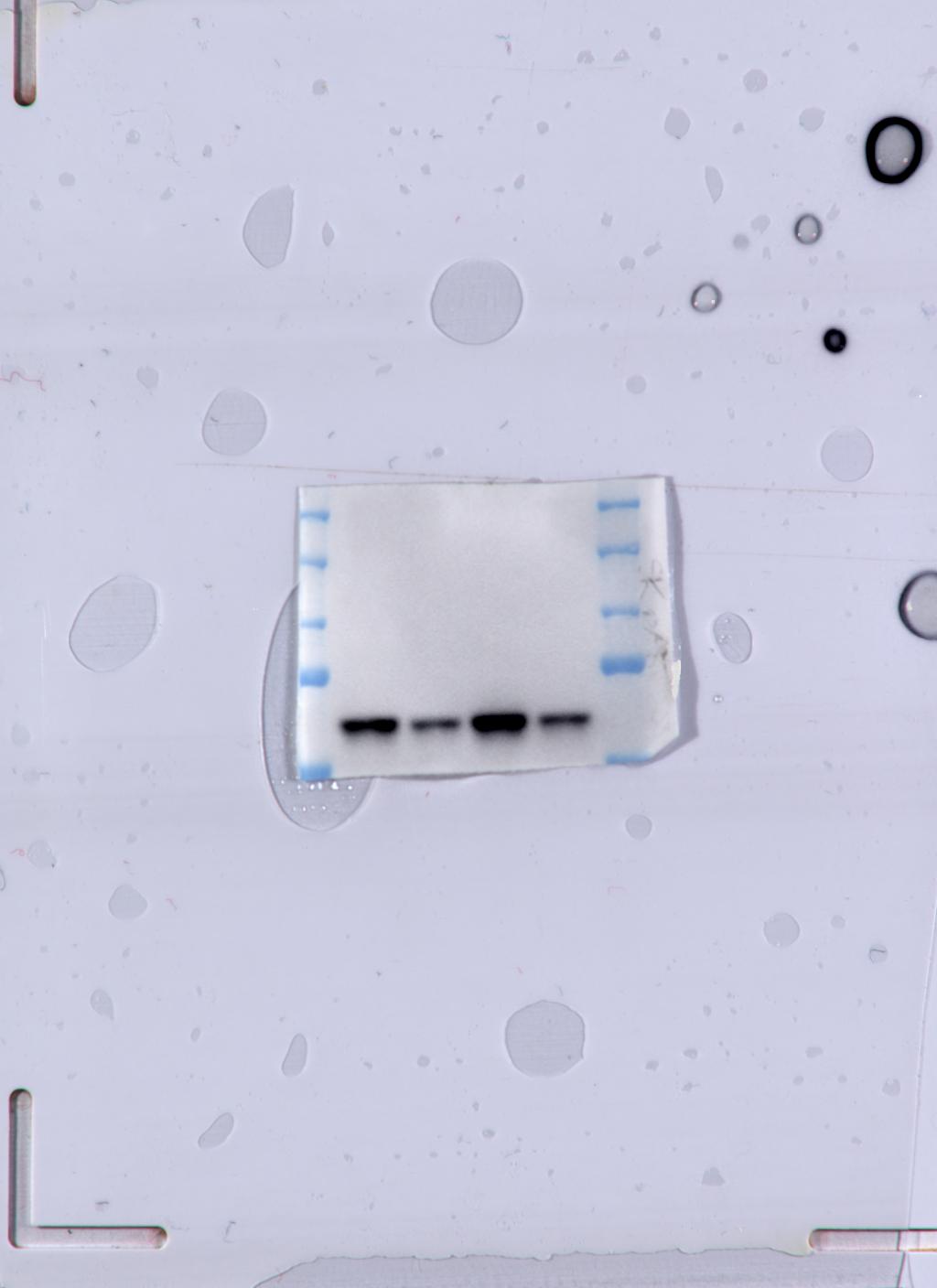

Supplement: Supplementary file 1 [file cancers-13-02778-s001.zip › Figure.S6/FigureS1/SK-BR-3/pAKT 308.jpg]

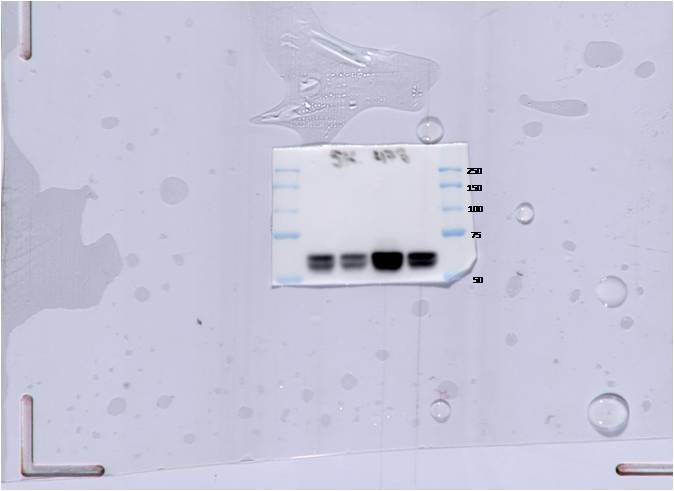

Supplement: Supplementary file 1 [file cancers-13-02778-s001.zip › Figure.S6/FigureS1/SK-BR-3/pAKT 473 MW.jpg]

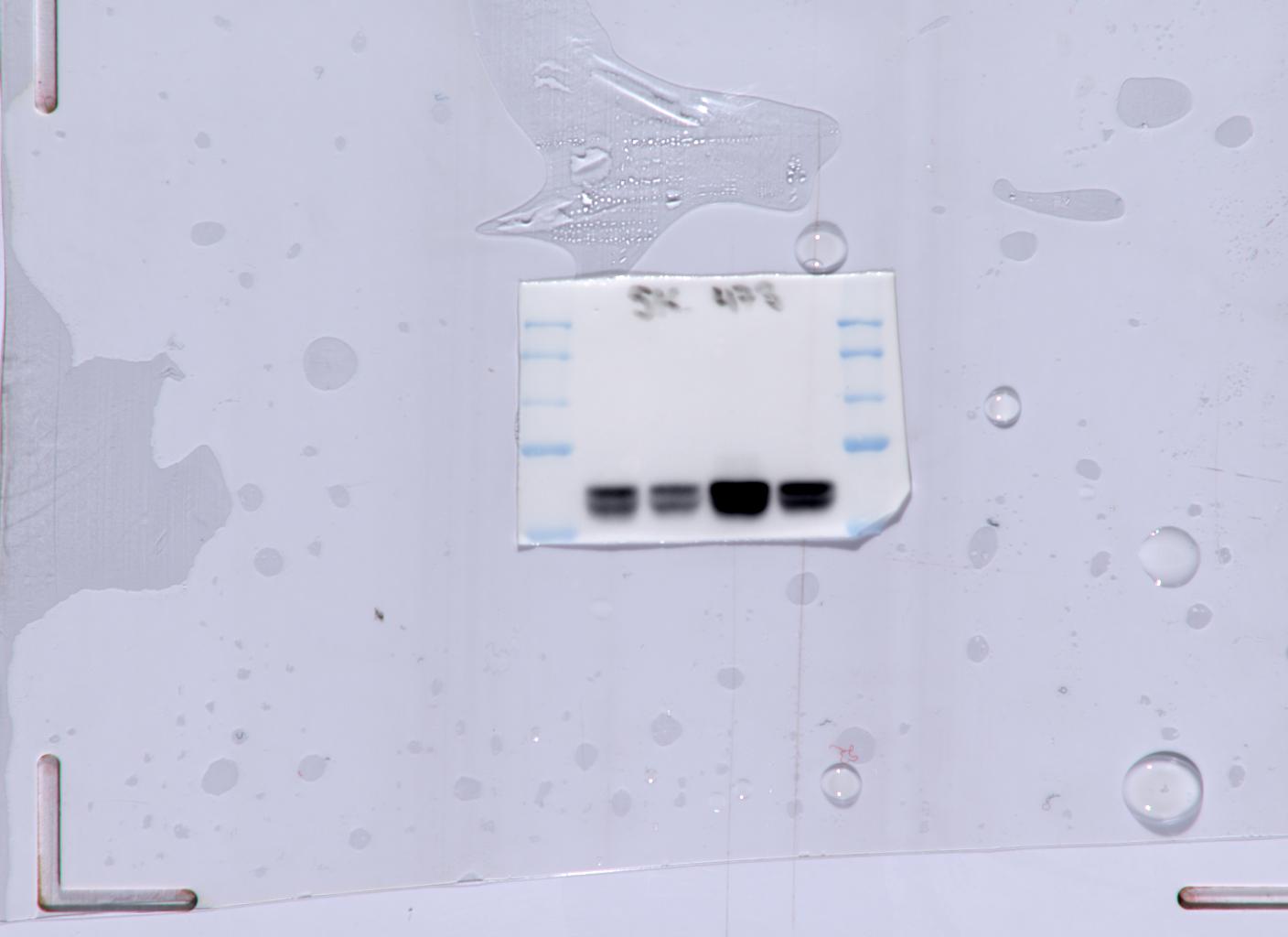

Supplement: Supplementary file 1 [file cancers-13-02778-s001.zip › Figure.S6/FigureS1/SK-BR-3/pAKT 473.jpg]

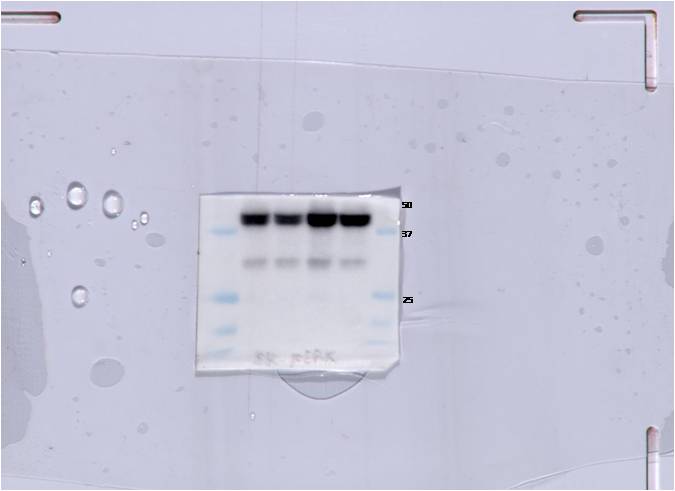

Supplement: Supplementary file 1 [file cancers-13-02778-s001.zip › Figure.S6/FigureS1/SK-BR-3/pERK MW.jpg]

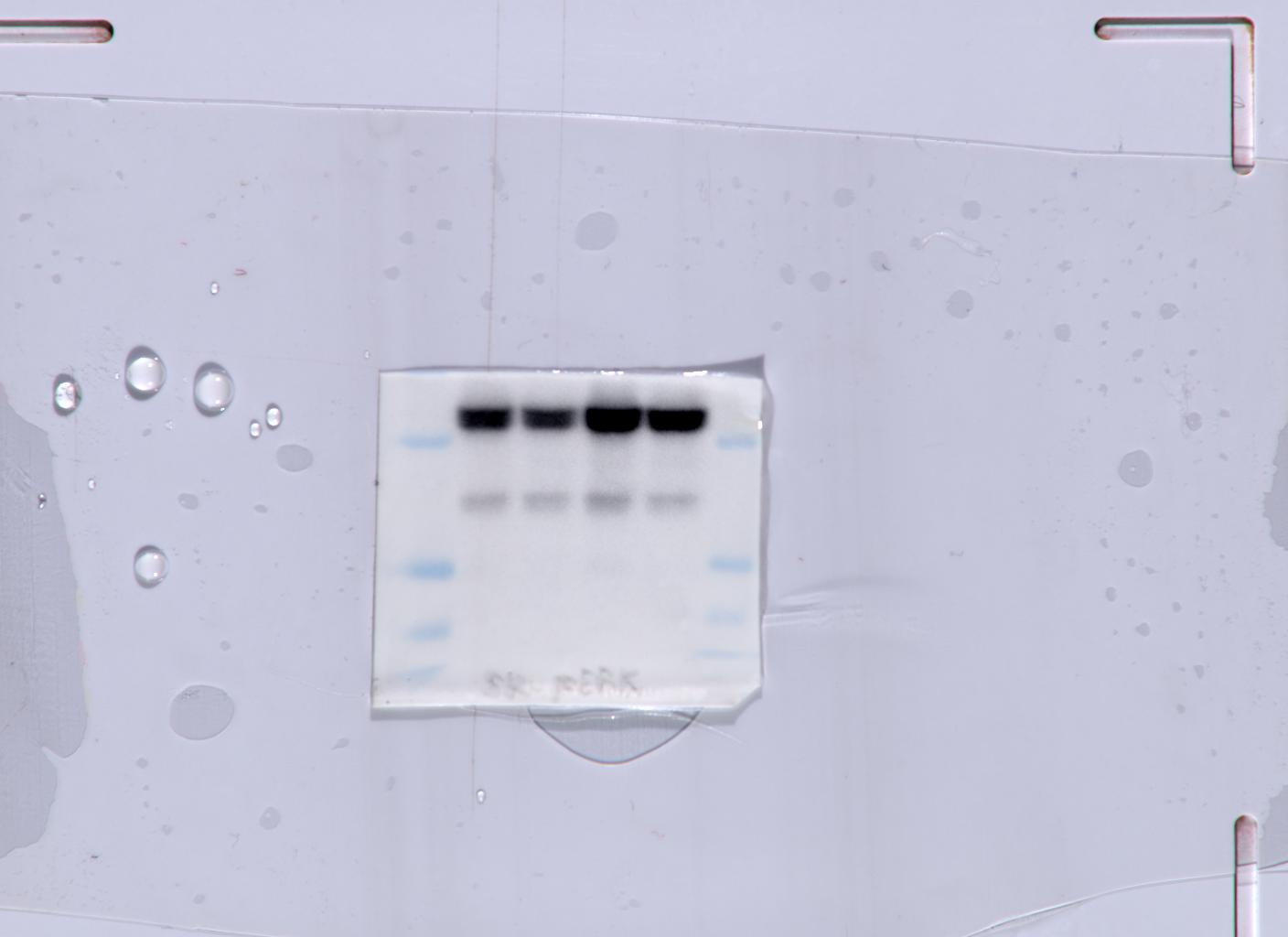

Supplement: Supplementary file 1 [file cancers-13-02778-s001.zip › Figure.S6/FigureS1/SK-BR-3/pERK.jpg]

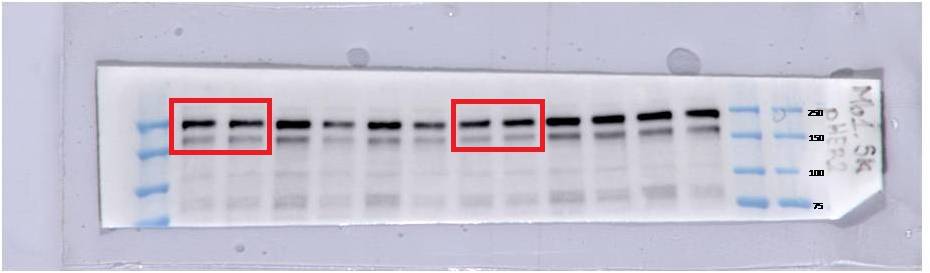

Supplement: Supplementary file 1 [file cancers-13-02778-s001.zip › Figure.S6/FigureS1/SK-BR-3/pHER2 MW.jpg]

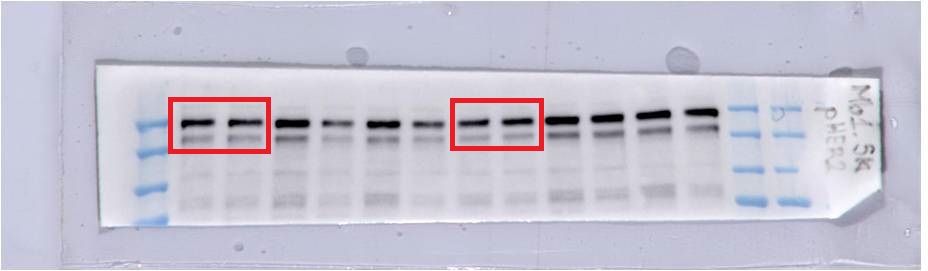

Supplement: Supplementary file 1 [file cancers-13-02778-s001.zip › Figure.S6/FigureS1/SK-BR-3/pHER2.jpg]

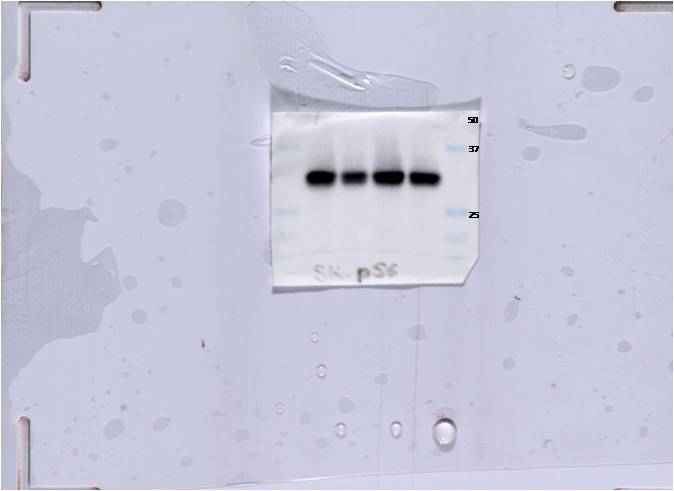

Supplement: Supplementary file 1 [file cancers-13-02778-s001.zip › Figure.S6/FigureS1/SK-BR-3/pS6 MW.jpg]

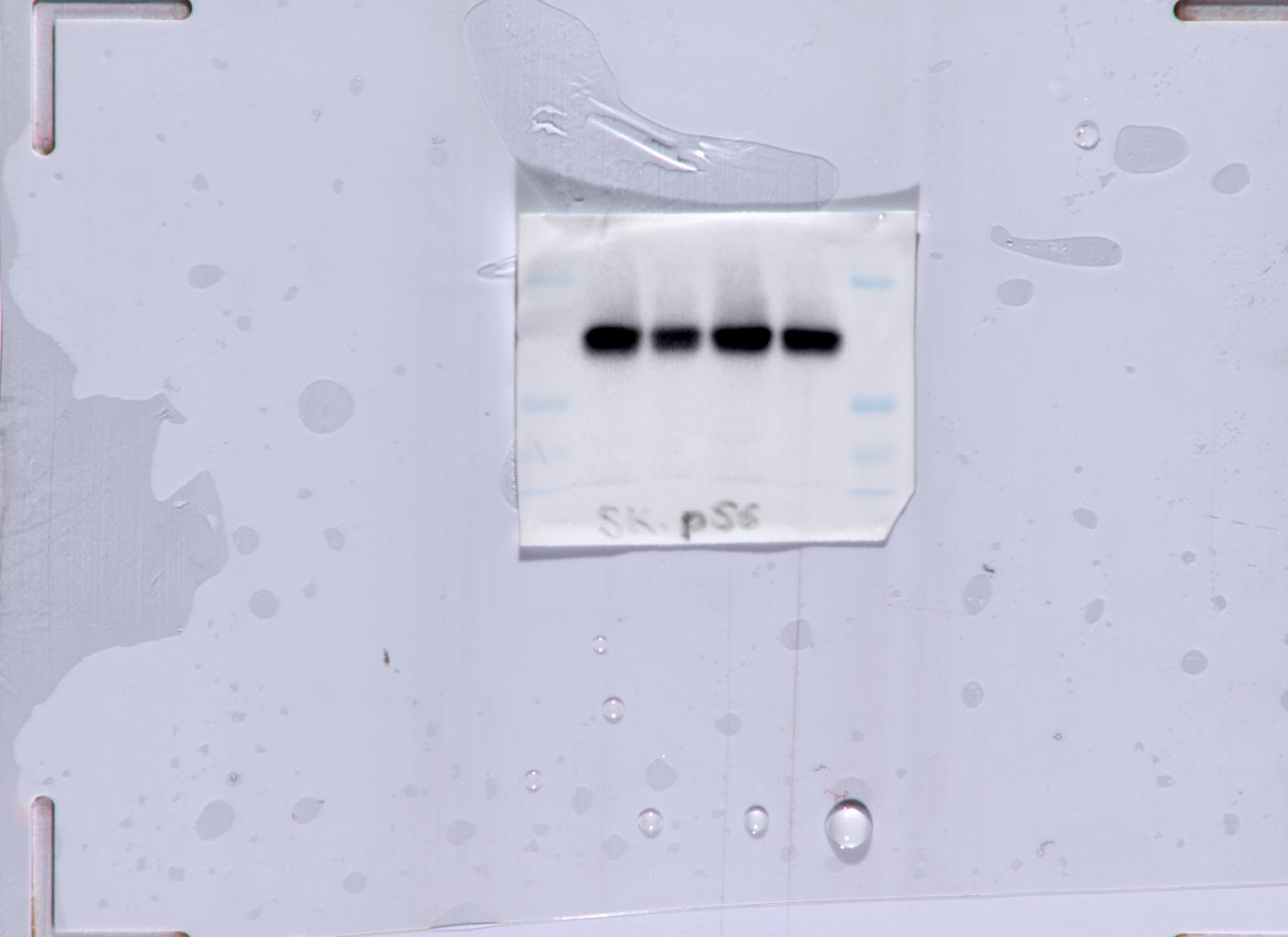

Supplement: Supplementary file 1 [file cancers-13-02778-s001.zip › Figure.S6/FigureS1/SK-BR-3/pS6.jpg]

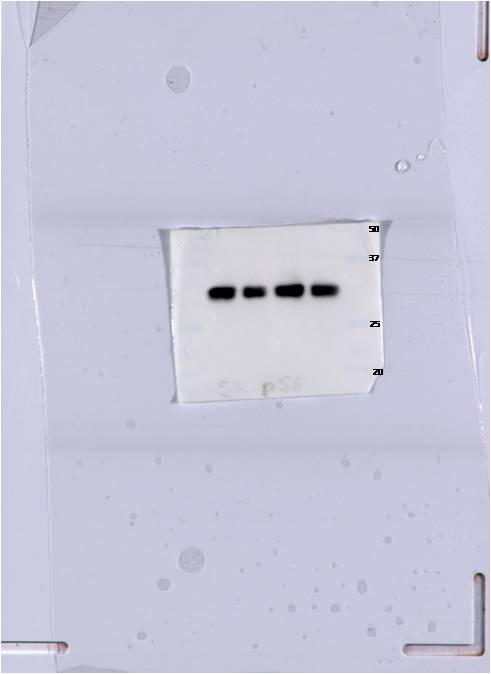

Supplement: Supplementary file 1 [file cancers-13-02778-s001.zip › Figure.S6/FigureS1/SK-BR-3/S6 MW.jpg]

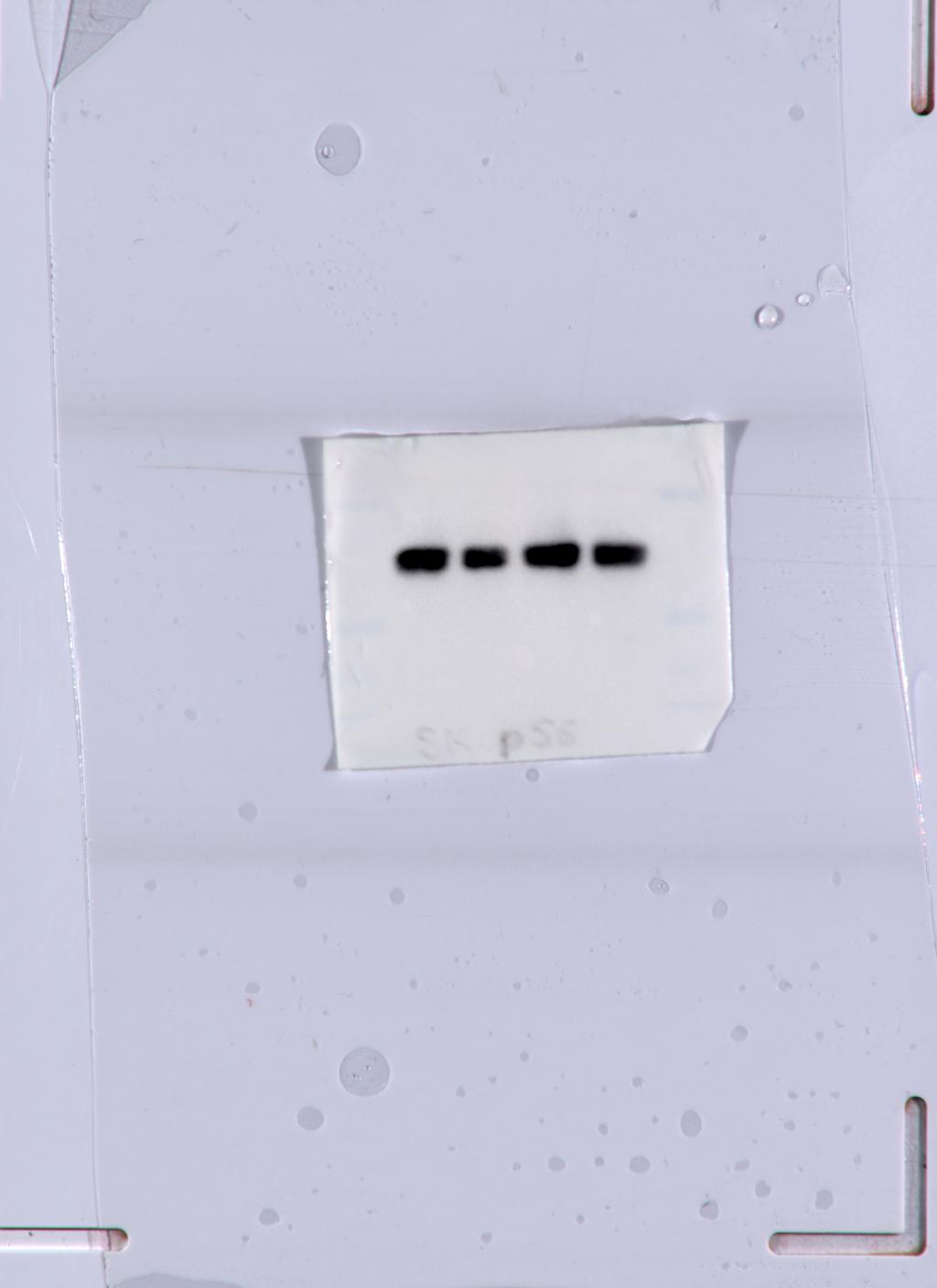

Supplement: Supplementary file 1 [file cancers-13-02778-s001.zip › Figure.S6/FigureS1/SK-BR-3/S6.jpg]

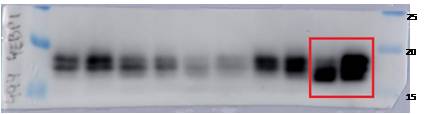

Supplement: Supplementary file 1 [file cancers-13-02778-s001.zip › Figure.S6/FigureS2/4EBP1 MW.jpg]
