# Supplementary figures and images for: The Novel Oral mTORC1/2 Inhibitor TAK-228 Reverses Trastuzumab Resistance in HER2-Positive Breast Cancer Models (part 1 of 2)
Source: Cancers (Basel). 2021 Jun 3;13(11):2778. doi: 10.3390/cancers13112778 (PMC8199905; doi:10.3390/cancers13112778)

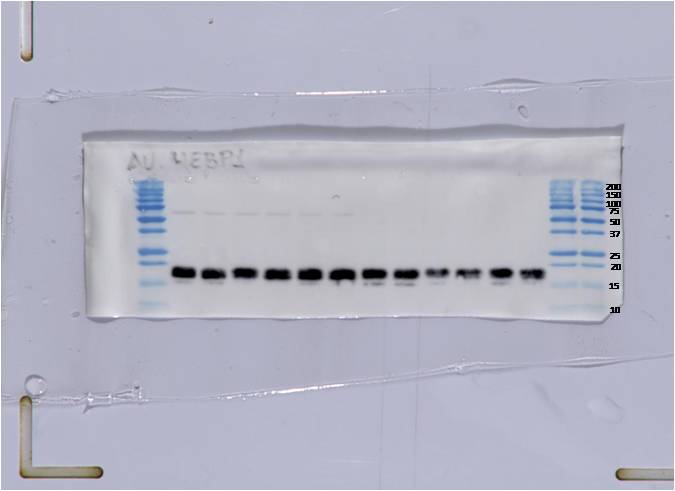

Supplement: Supplementary file 1 [file cancers-13-02778-s001.zip › Figure.S6/Figure5/AU565-AURT2.rT2/4EBP1 MW.jpg]

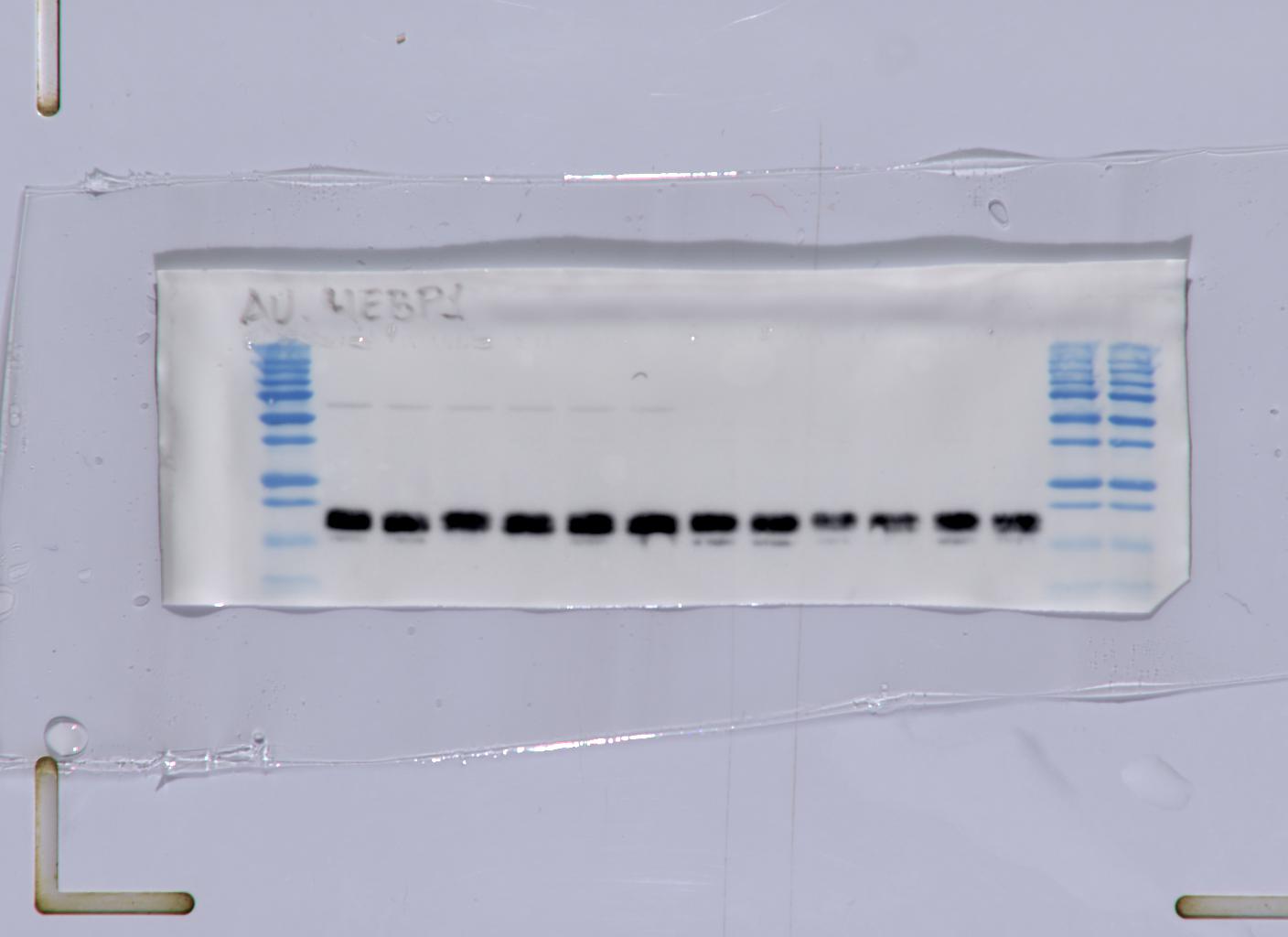

Supplement: Supplementary file 1 [file cancers-13-02778-s001.zip › Figure.S6/Figure5/AU565-AURT2.rT2/4EBP1.jpg]

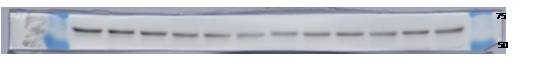

Supplement: Supplementary file 1 [file cancers-13-02778-s001.zip › Figure.S6/Figure5/AU565-AURT2.rT2/AKT MW.jpg]

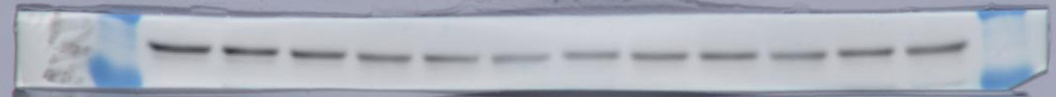

Supplement: Supplementary file 1 [file cancers-13-02778-s001.zip › Figure.S6/Figure5/AU565-AURT2.rT2/AKT.jpg]

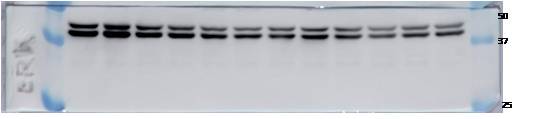

Supplement: Supplementary file 1 [file cancers-13-02778-s001.zip › Figure.S6/Figure5/AU565-AURT2.rT2/ERK MW.jpg]

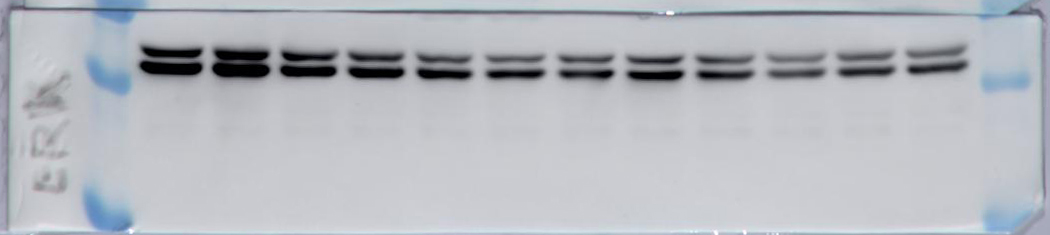

Supplement: Supplementary file 1 [file cancers-13-02778-s001.zip › Figure.S6/Figure5/AU565-AURT2.rT2/ERK.jpg]

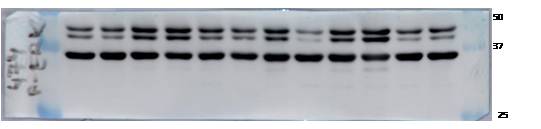

Supplement: Supplementary file 1 [file cancers-13-02778-s001.zip › Figure.S6/Figure5/AU565-AURT2.rT2/GAPDH MW.jpg]

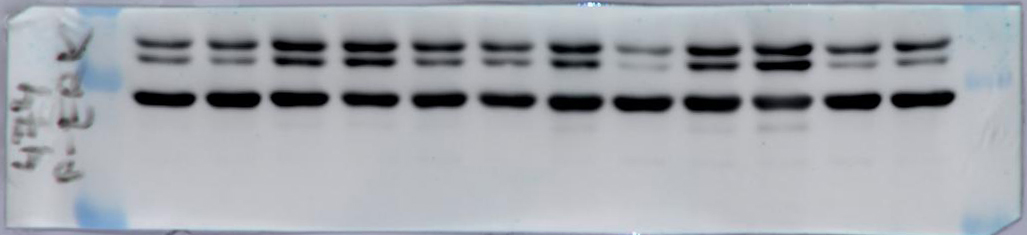

Supplement: Supplementary file 1 [file cancers-13-02778-s001.zip › Figure.S6/Figure5/AU565-AURT2.rT2/GAPDH.jpg]

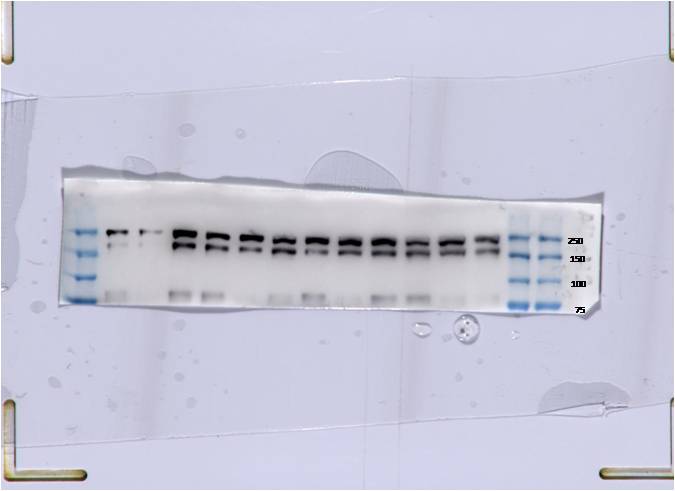

Supplement: Supplementary file 1 [file cancers-13-02778-s001.zip › Figure.S6/Figure5/AU565-AURT2.rT2/HER2 MW.jpg]

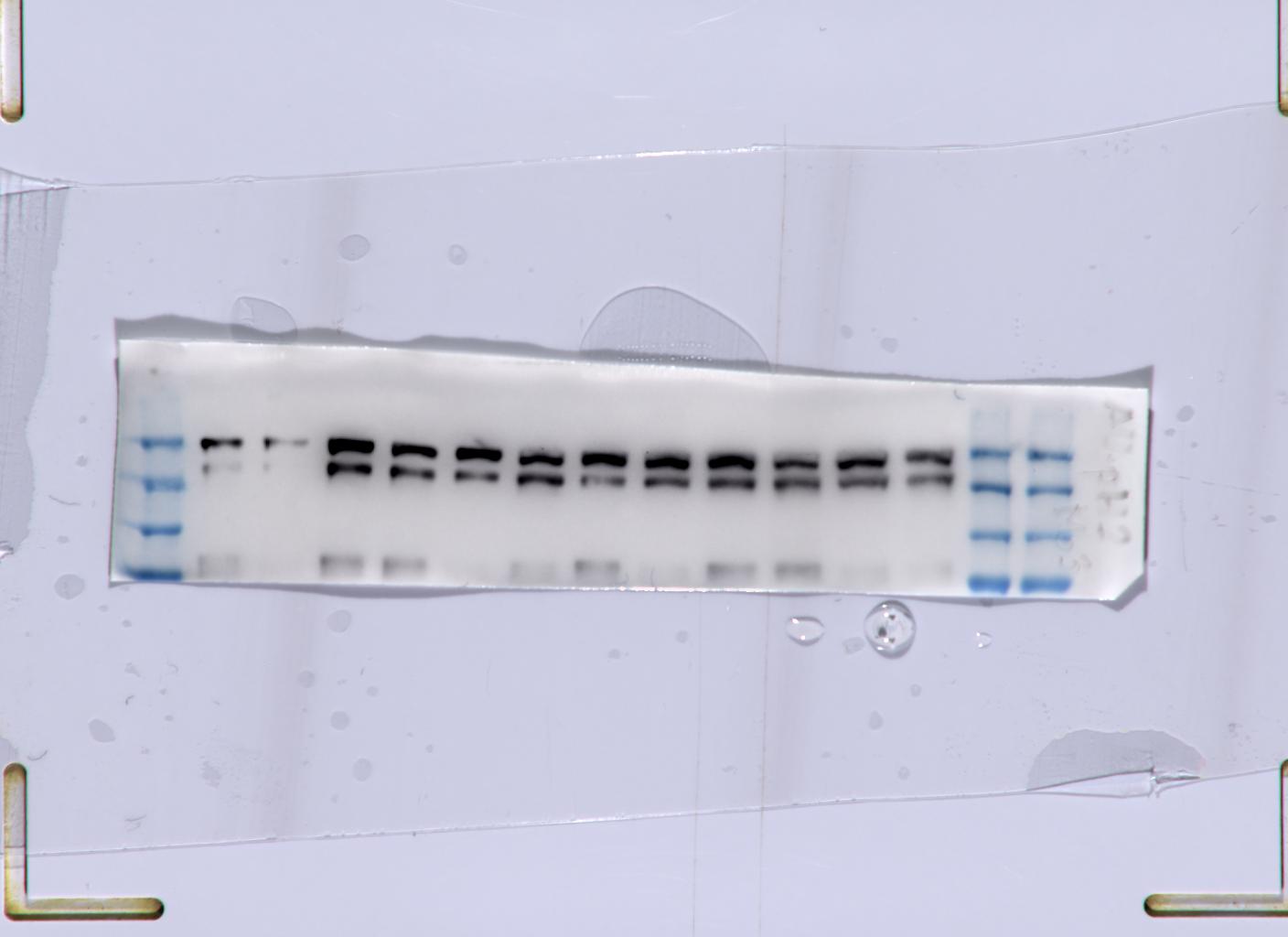

Supplement: Supplementary file 1 [file cancers-13-02778-s001.zip › Figure.S6/Figure5/AU565-AURT2.rT2/HER2.jpg]

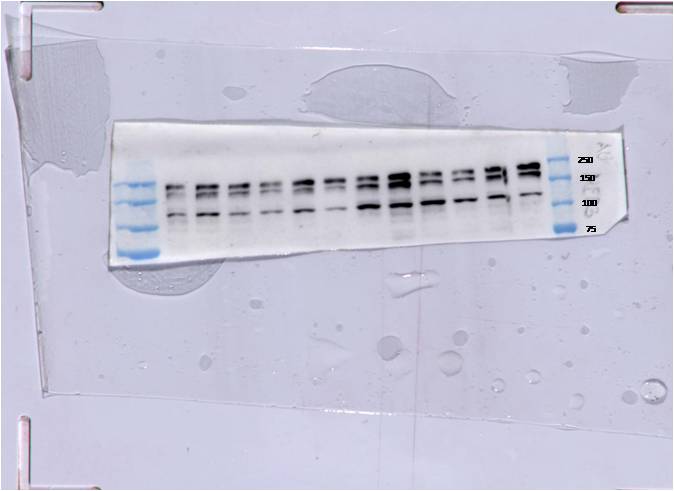

Supplement: Supplementary file 1 [file cancers-13-02778-s001.zip › Figure.S6/Figure5/AU565-AURT2.rT2/HER3 MW.jpg]

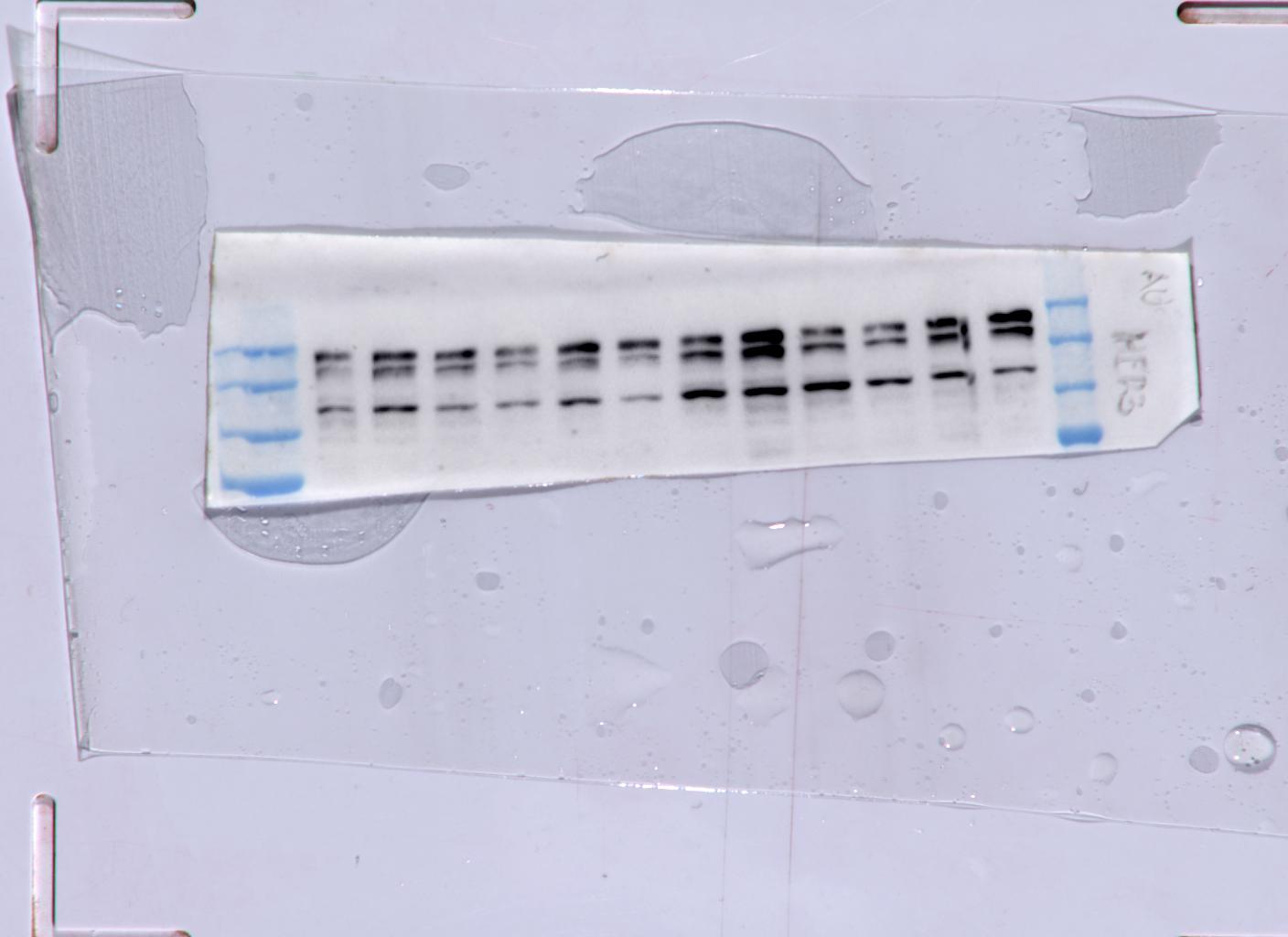

Supplement: Supplementary file 1 [file cancers-13-02778-s001.zip › Figure.S6/Figure5/AU565-AURT2.rT2/HER3.jpg]

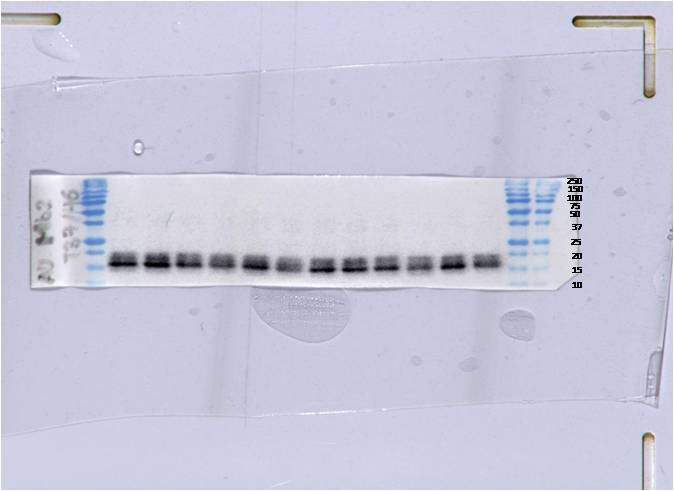

Supplement: Supplementary file 1 [file cancers-13-02778-s001.zip › Figure.S6/Figure5/AU565-AURT2.rT2/p4EBP1 T37 46 MW.jpg]

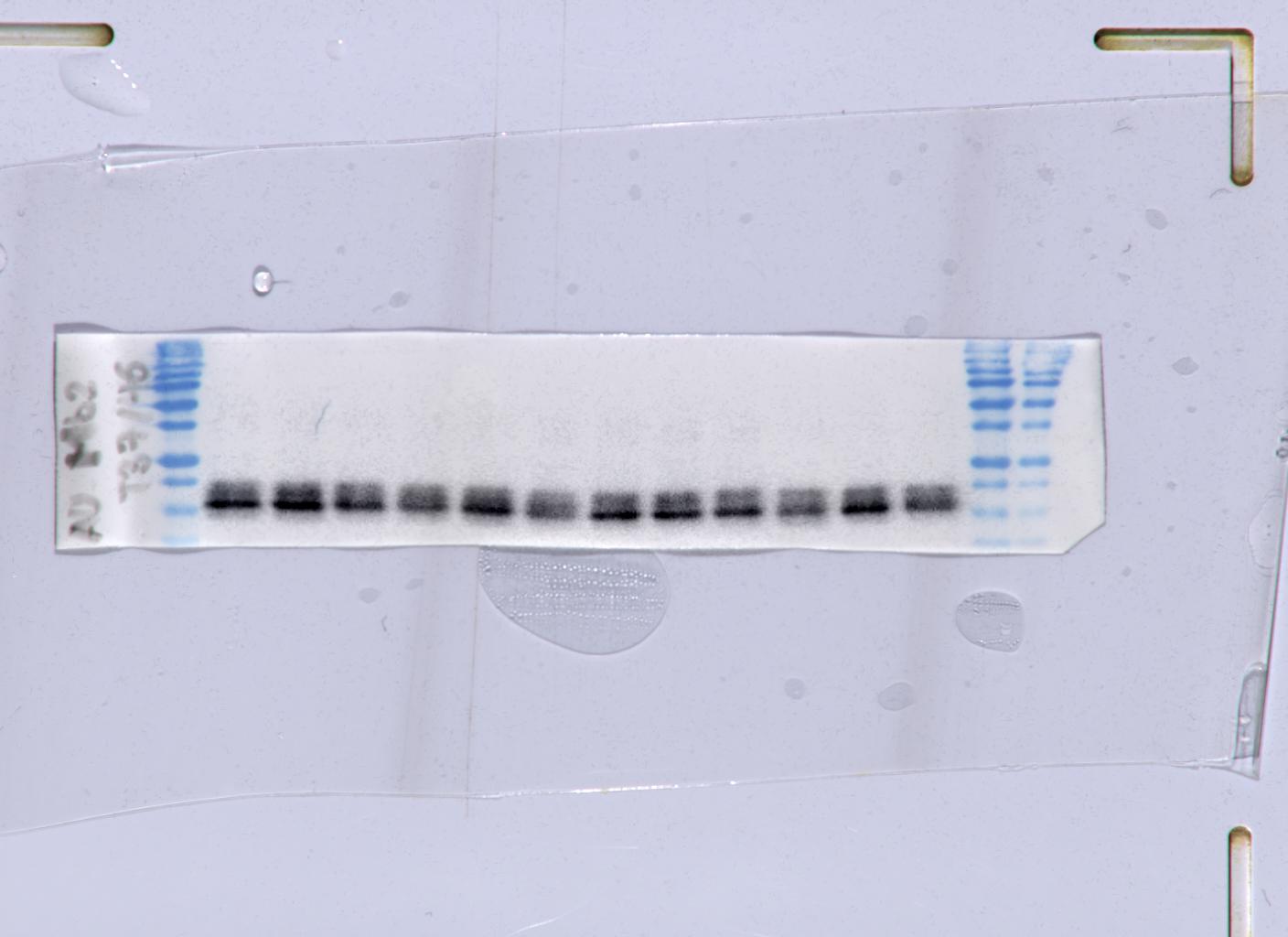

Supplement: Supplementary file 1 [file cancers-13-02778-s001.zip › Figure.S6/Figure5/AU565-AURT2.rT2/p4EBP1 T37 46.jpg]

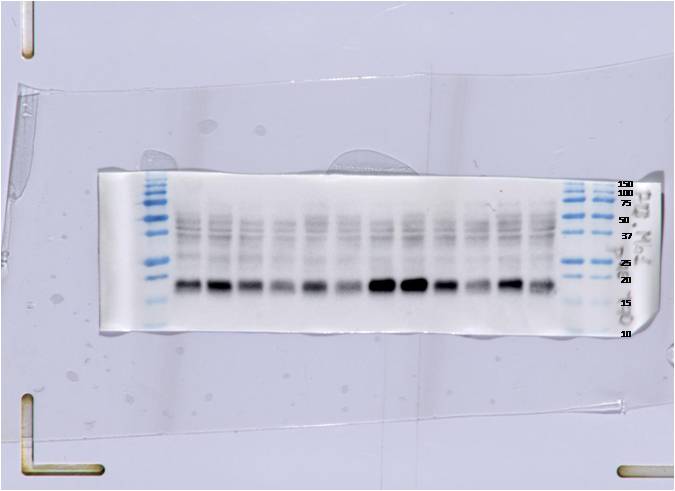

Supplement: Supplementary file 1 [file cancers-13-02778-s001.zip › Figure.S6/Figure5/AU565-AURT2.rT2/p4EBP1 T70 MW.jpg]

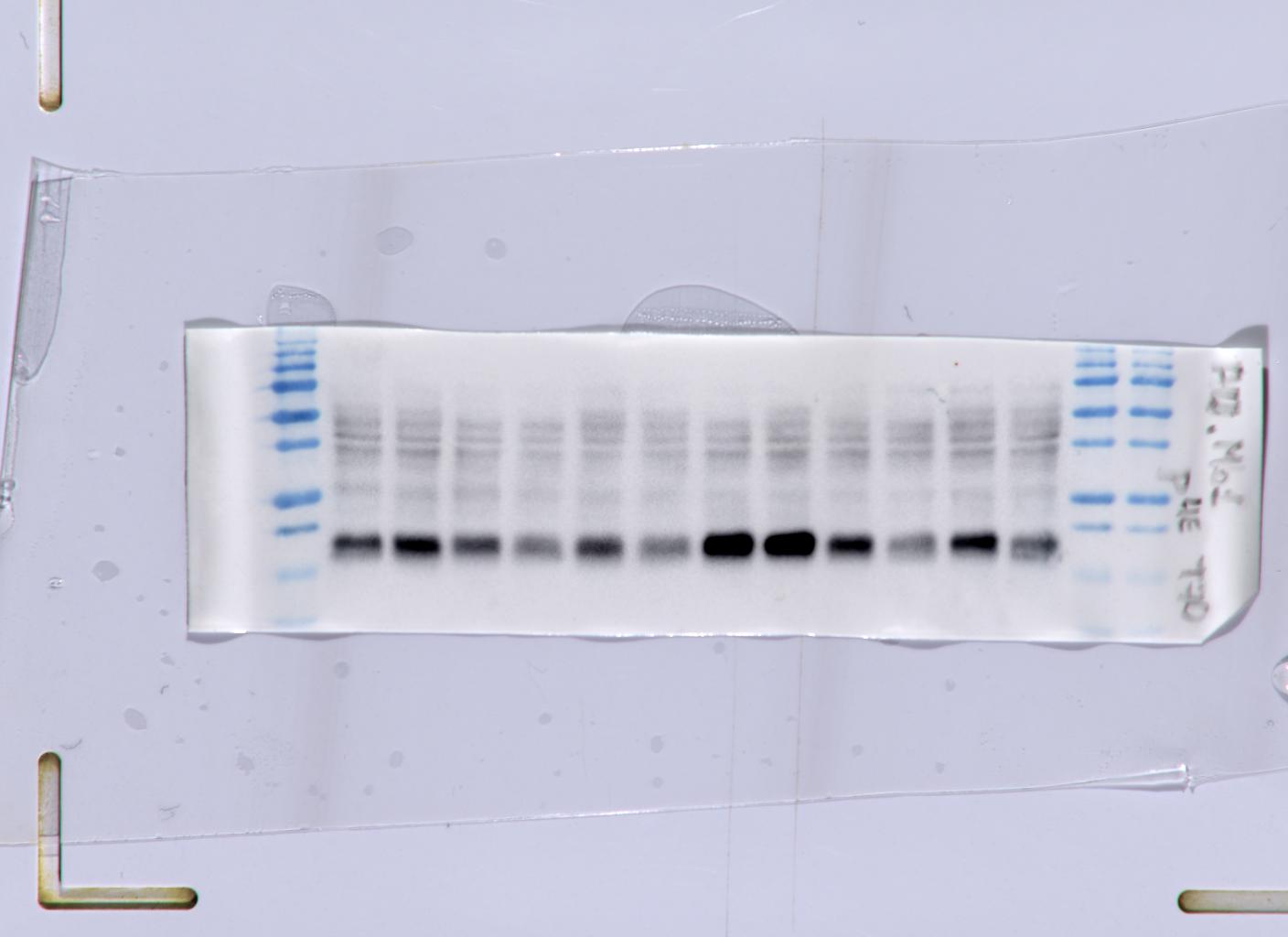

Supplement: Supplementary file 1 [file cancers-13-02778-s001.zip › Figure.S6/Figure5/AU565-AURT2.rT2/p4EBP1 T70.jpg]

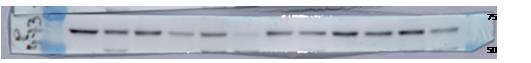

Supplement: Supplementary file 1 [file cancers-13-02778-s001.zip › Figure.S6/Figure5/AU565-AURT2.rT2/pAKT 473 MW.jpg]

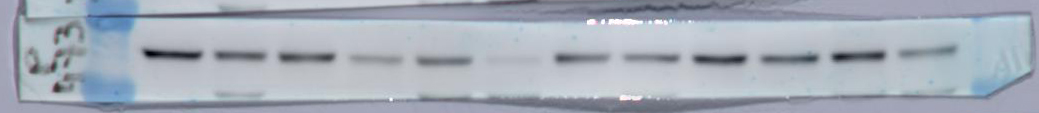

Supplement: Supplementary file 1 [file cancers-13-02778-s001.zip › Figure.S6/Figure5/AU565-AURT2.rT2/pAKT 473.jpg]

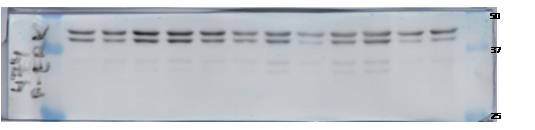

Supplement: Supplementary file 1 [file cancers-13-02778-s001.zip › Figure.S6/Figure5/AU565-AURT2.rT2/pERK MW.jpg]

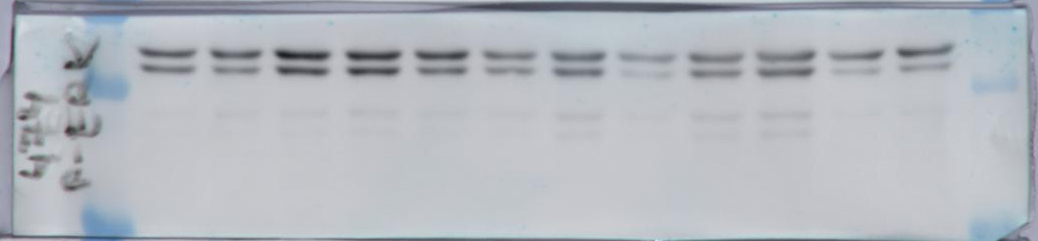

Supplement: Supplementary file 1 [file cancers-13-02778-s001.zip › Figure.S6/Figure5/AU565-AURT2.rT2/pERK.jpg]

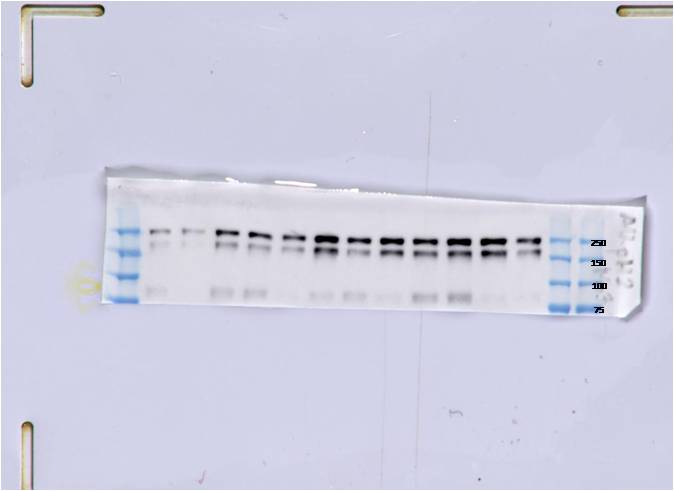

Supplement: Supplementary file 1 [file cancers-13-02778-s001.zip › Figure.S6/Figure5/AU565-AURT2.rT2/pHER2 MW.jpg]

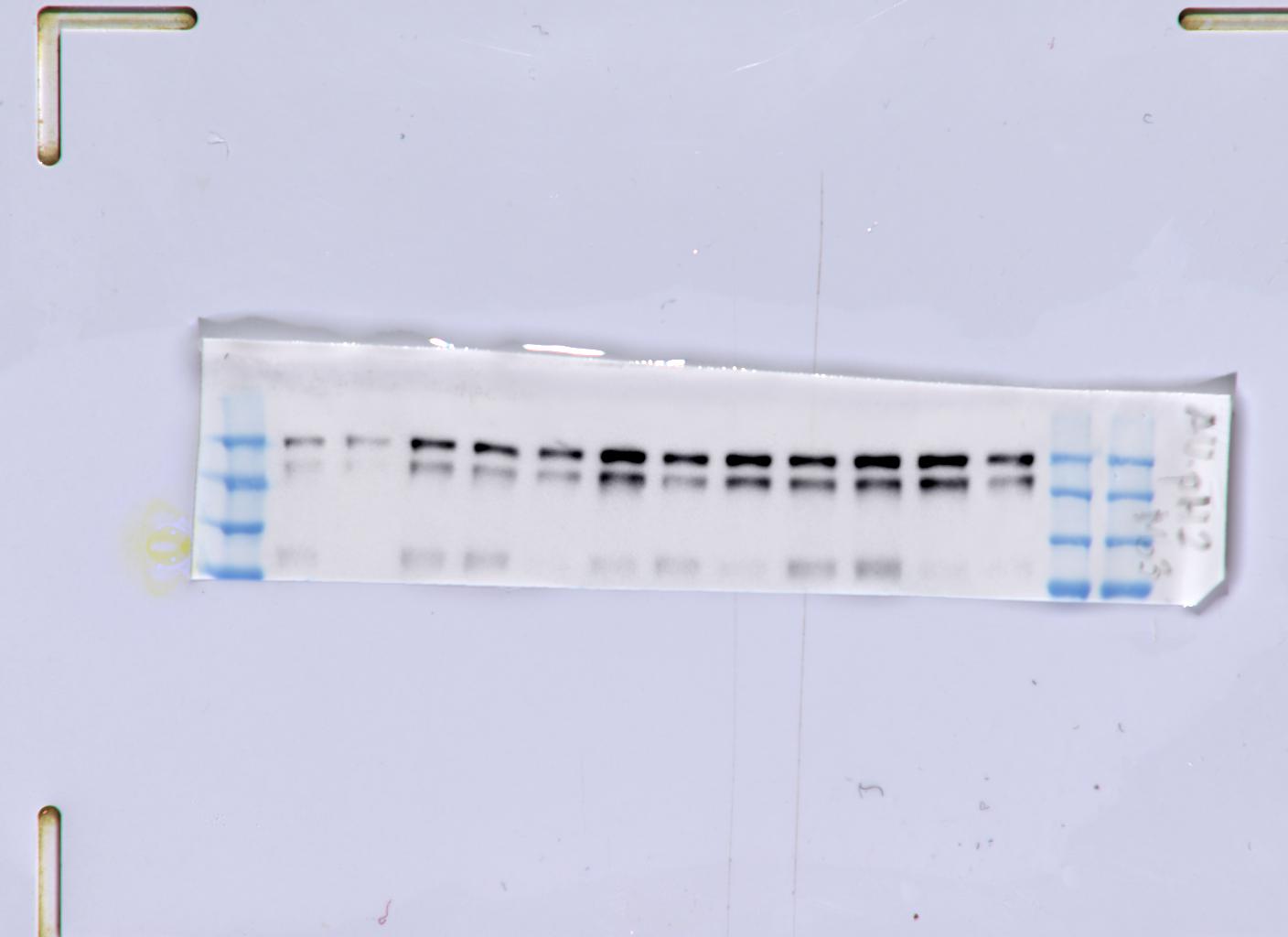

Supplement: Supplementary file 1 [file cancers-13-02778-s001.zip › Figure.S6/Figure5/AU565-AURT2.rT2/pHER2.jpg]

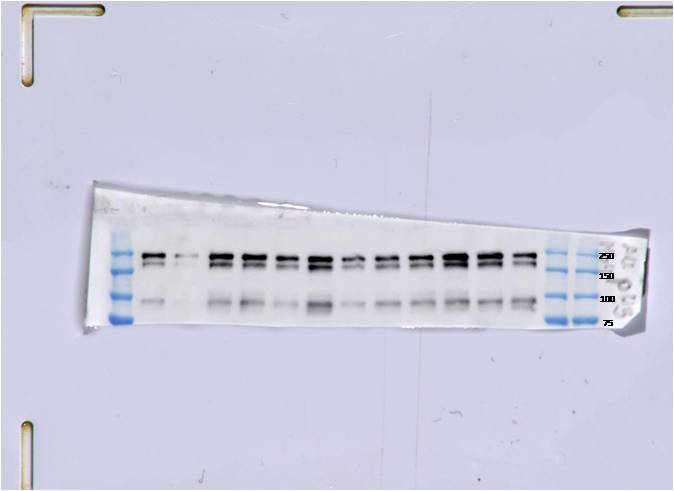

Supplement: Supplementary file 1 [file cancers-13-02778-s001.zip › Figure.S6/Figure5/AU565-AURT2.rT2/pHER3 MW.jpg]

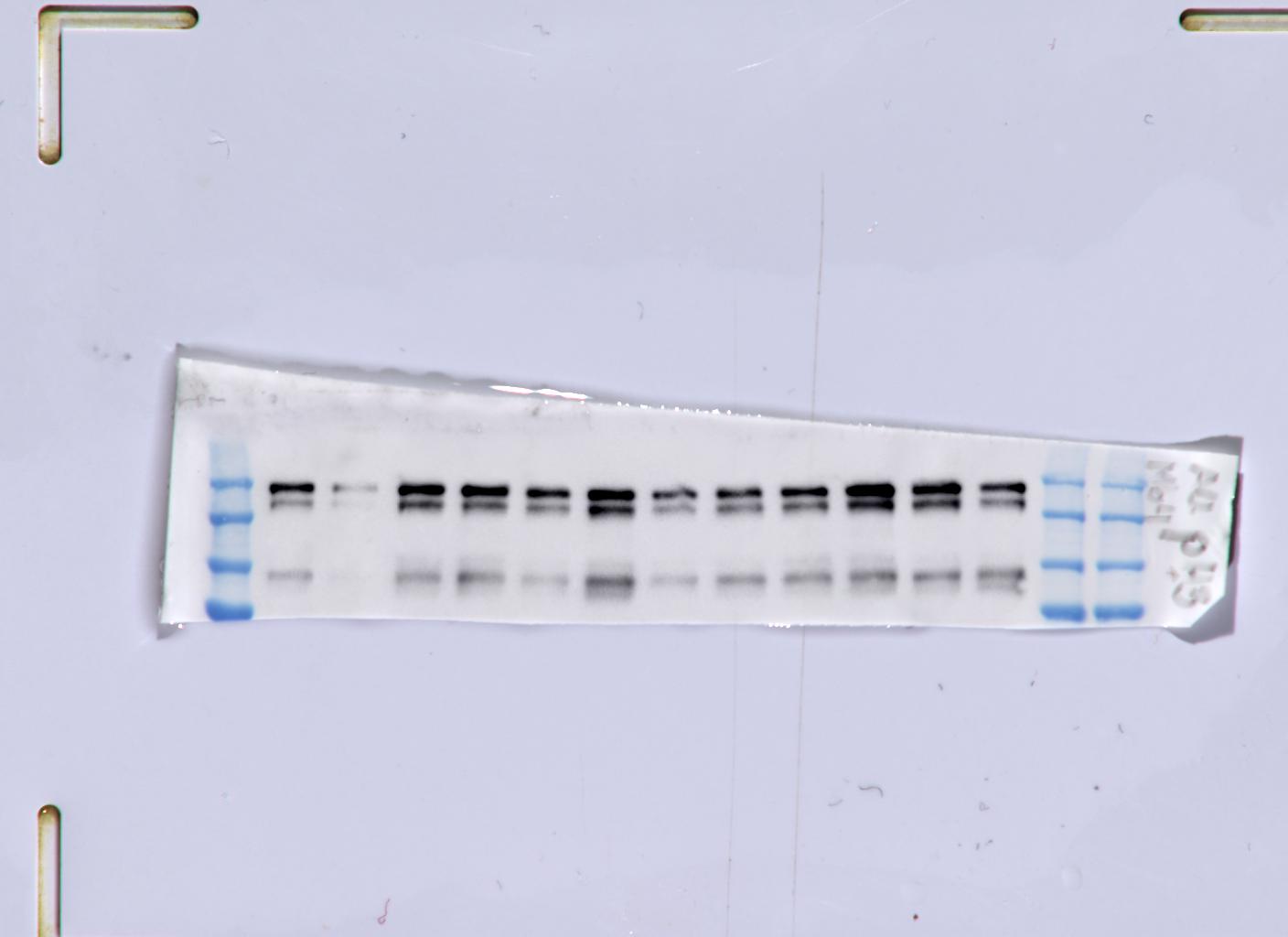

Supplement: Supplementary file 1 [file cancers-13-02778-s001.zip › Figure.S6/Figure5/AU565-AURT2.rT2/pHER3.jpg]

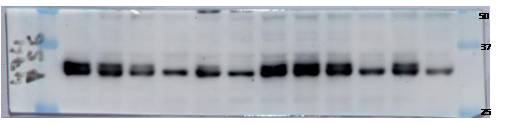

Supplement: Supplementary file 1 [file cancers-13-02778-s001.zip › Figure.S6/Figure5/AU565-AURT2.rT2/pS6 MW.jpg]

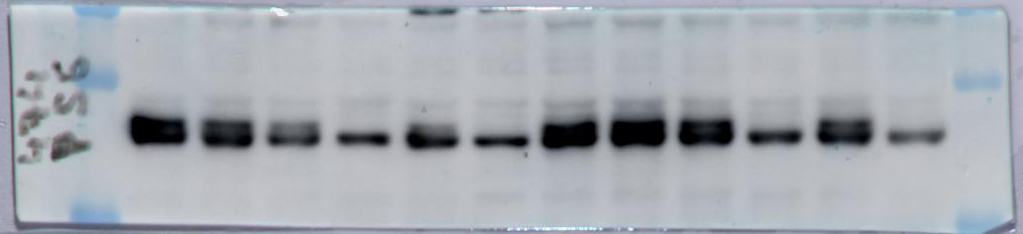

Supplement: Supplementary file 1 [file cancers-13-02778-s001.zip › Figure.S6/Figure5/AU565-AURT2.rT2/pS6.jpg]

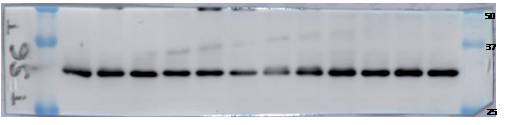

Supplement: Supplementary file 1 [file cancers-13-02778-s001.zip › Figure.S6/Figure5/AU565-AURT2.rT2/S6 MW.jpg]

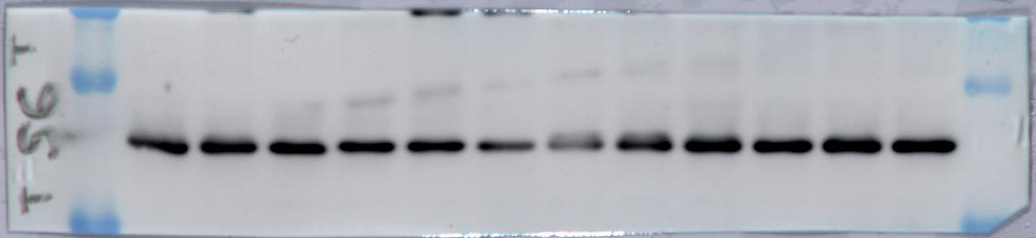

Supplement: Supplementary file 1 [file cancers-13-02778-s001.zip › Figure.S6/Figure5/AU565-AURT2.rT2/S6.jpg]

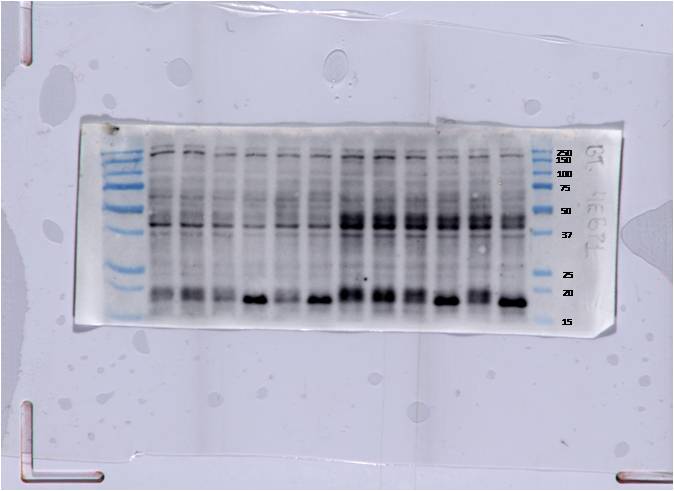

Supplement: Supplementary file 1 [file cancers-13-02778-s001.zip › Figure.S6/Figure5/BT474-BT474.rT3/4EBP1 MW.jpg]

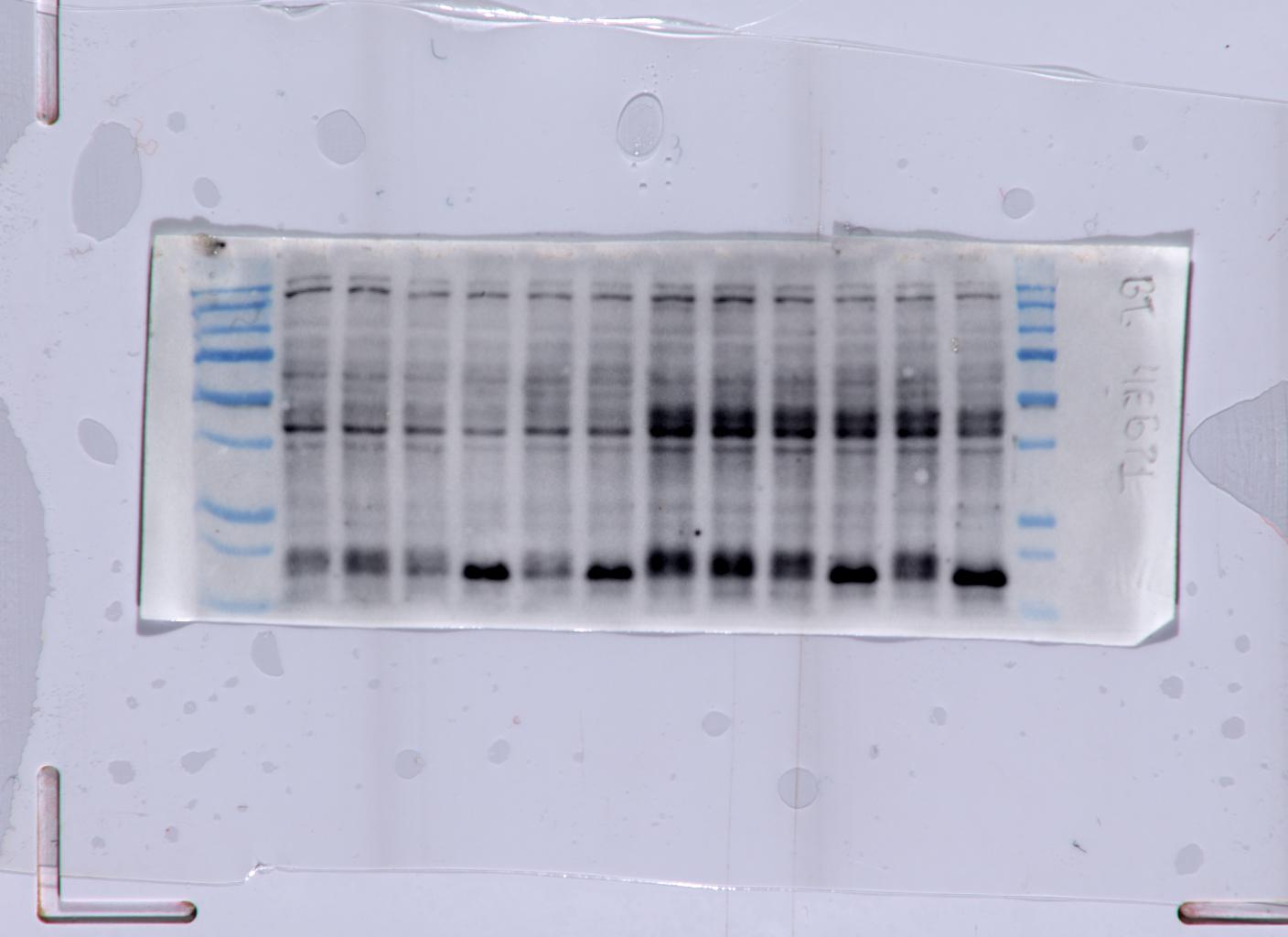

Supplement: Supplementary file 1 [file cancers-13-02778-s001.zip › Figure.S6/Figure5/BT474-BT474.rT3/4EBP1.jpg]

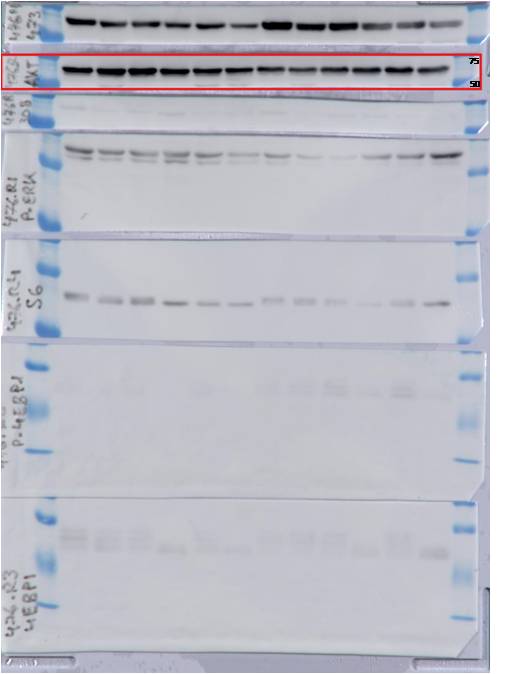

Supplement: Supplementary file 1 [file cancers-13-02778-s001.zip › Figure.S6/Figure5/BT474-BT474.rT3/AKT MW.jpg]

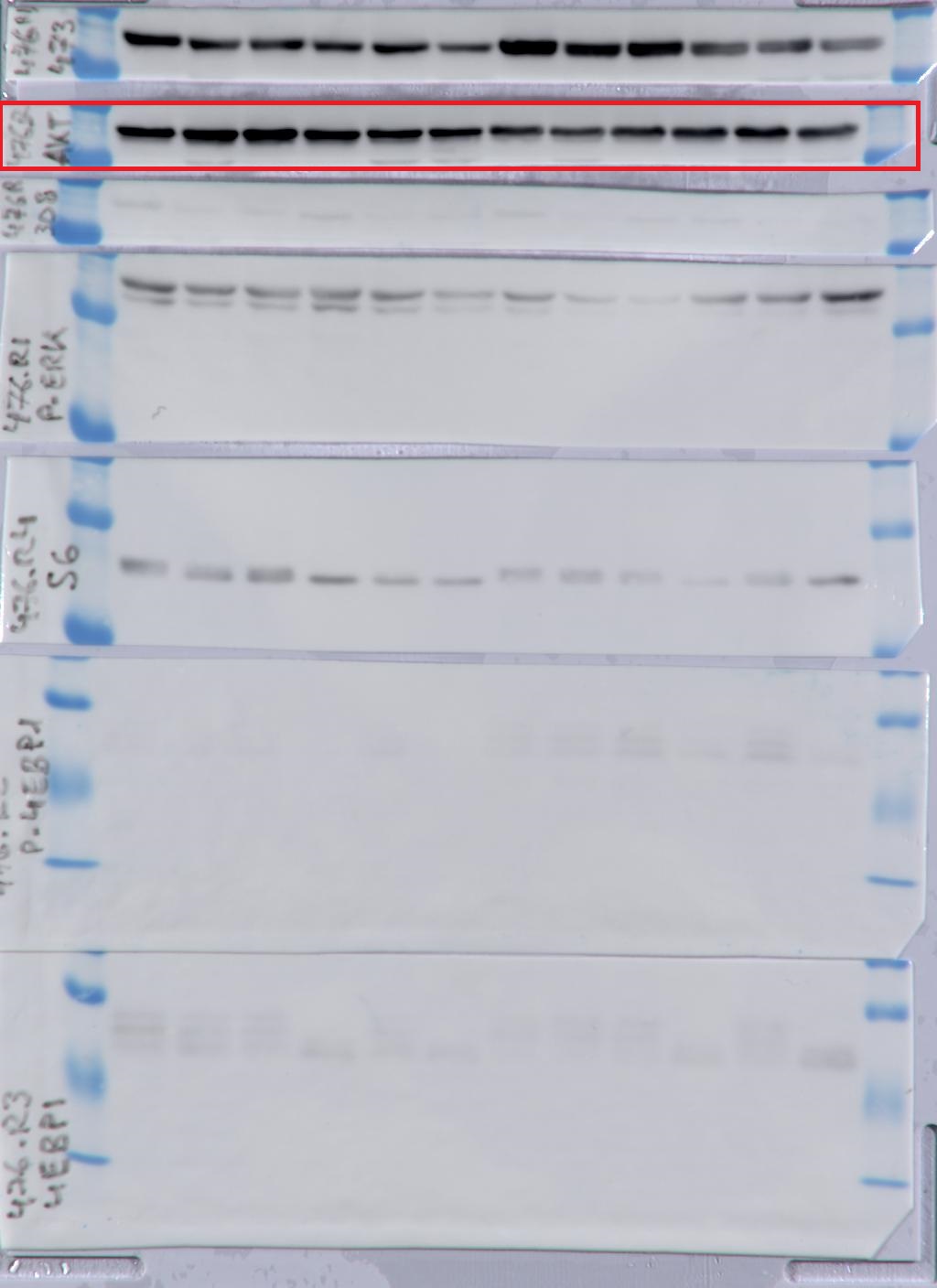

Supplement: Supplementary file 1 [file cancers-13-02778-s001.zip › Figure.S6/Figure5/BT474-BT474.rT3/AKT.jpg]

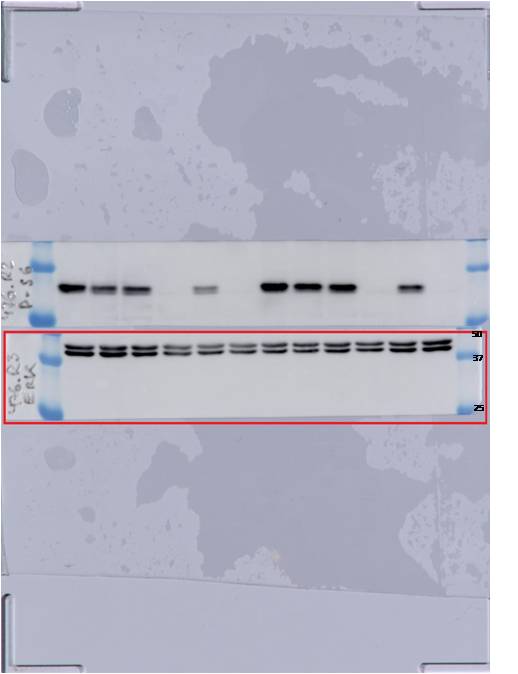

Supplement: Supplementary file 1 [file cancers-13-02778-s001.zip › Figure.S6/Figure5/BT474-BT474.rT3/ERK MW.jpg]

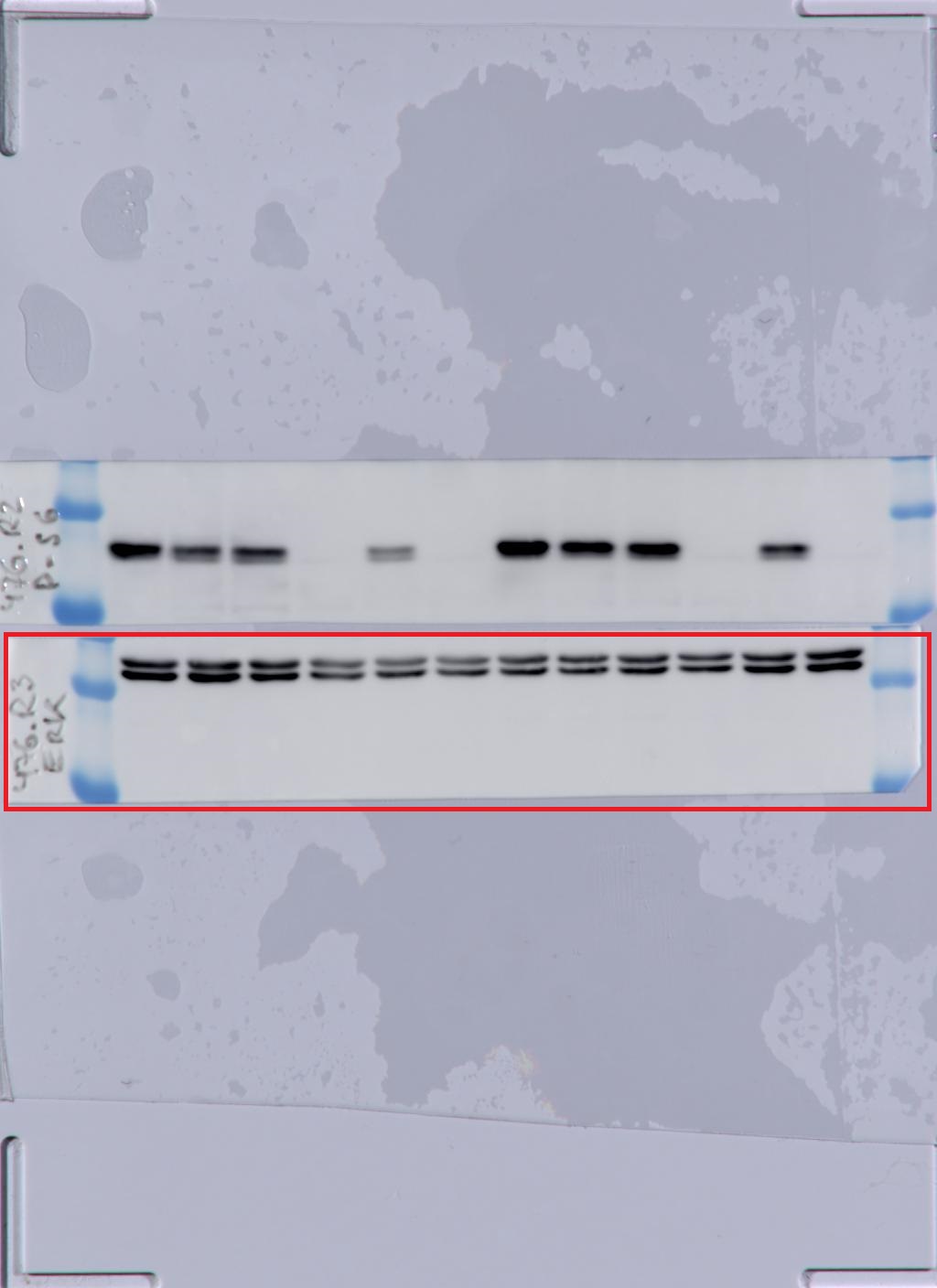

Supplement: Supplementary file 1 [file cancers-13-02778-s001.zip › Figure.S6/Figure5/BT474-BT474.rT3/ERK.jpg]

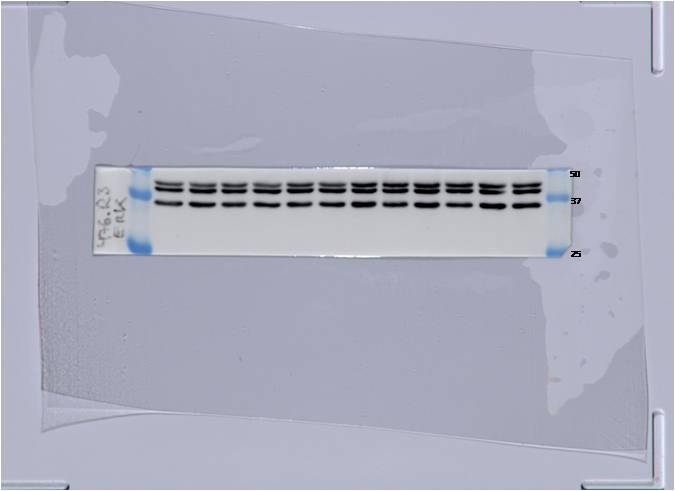

Supplement: Supplementary file 1 [file cancers-13-02778-s001.zip › Figure.S6/Figure5/BT474-BT474.rT3/GAPDH MW.jpg]

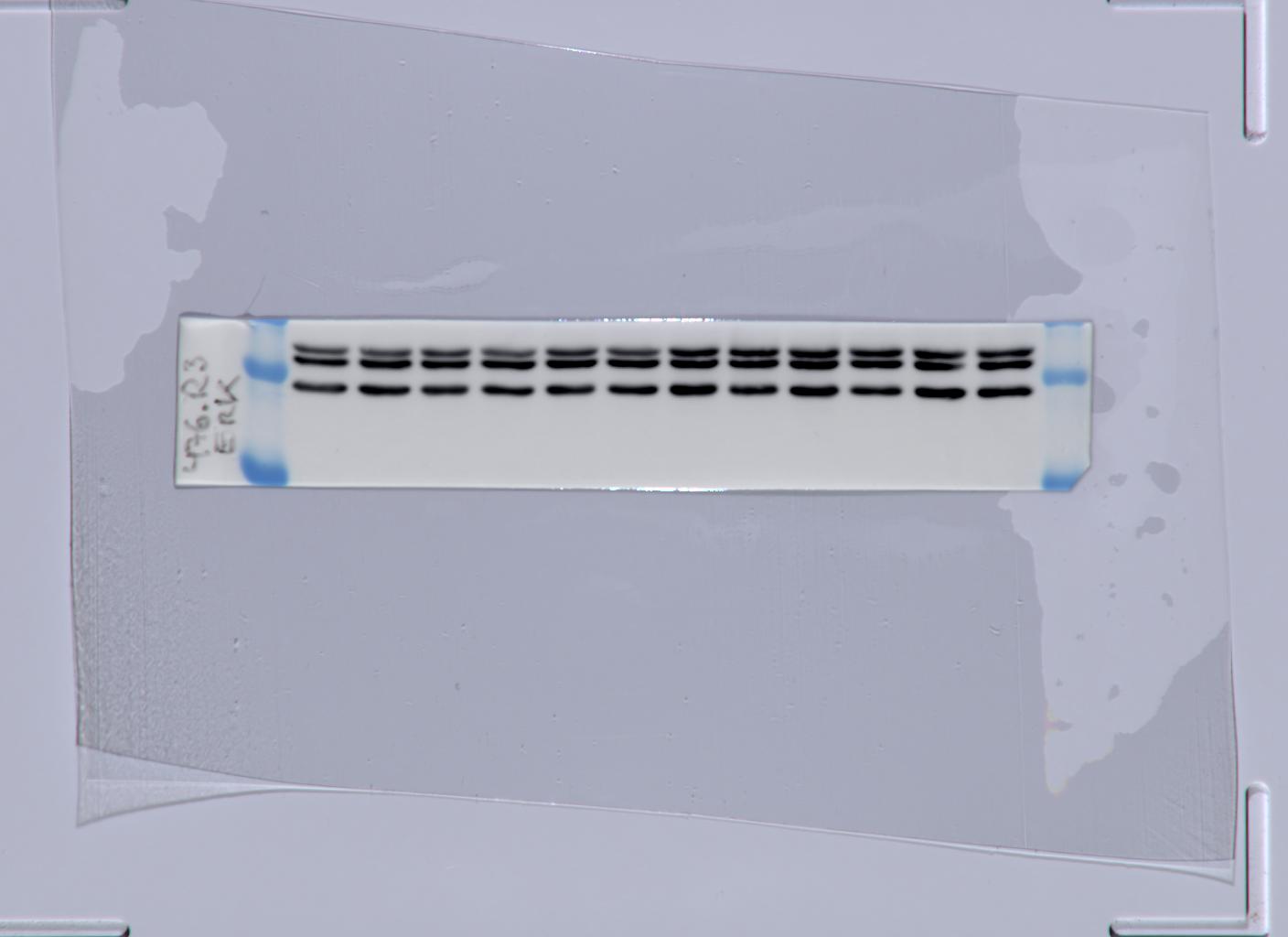

Supplement: Supplementary file 1 [file cancers-13-02778-s001.zip › Figure.S6/Figure5/BT474-BT474.rT3/GAPDH.jpg]

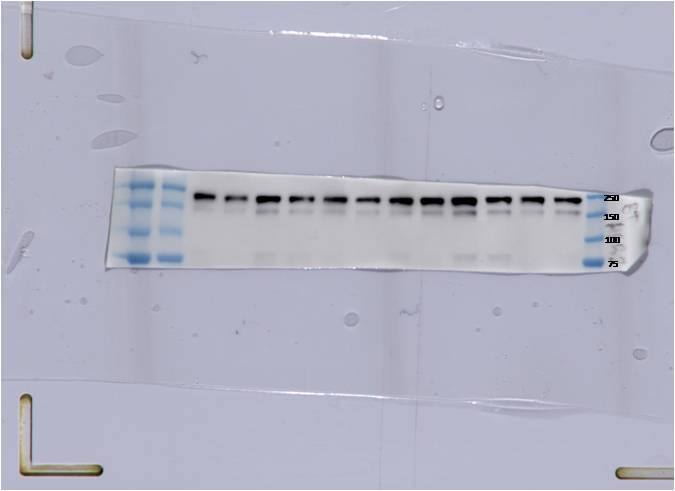

Supplement: Supplementary file 1 [file cancers-13-02778-s001.zip › Figure.S6/Figure5/BT474-BT474.rT3/HER2 MW.jpg]

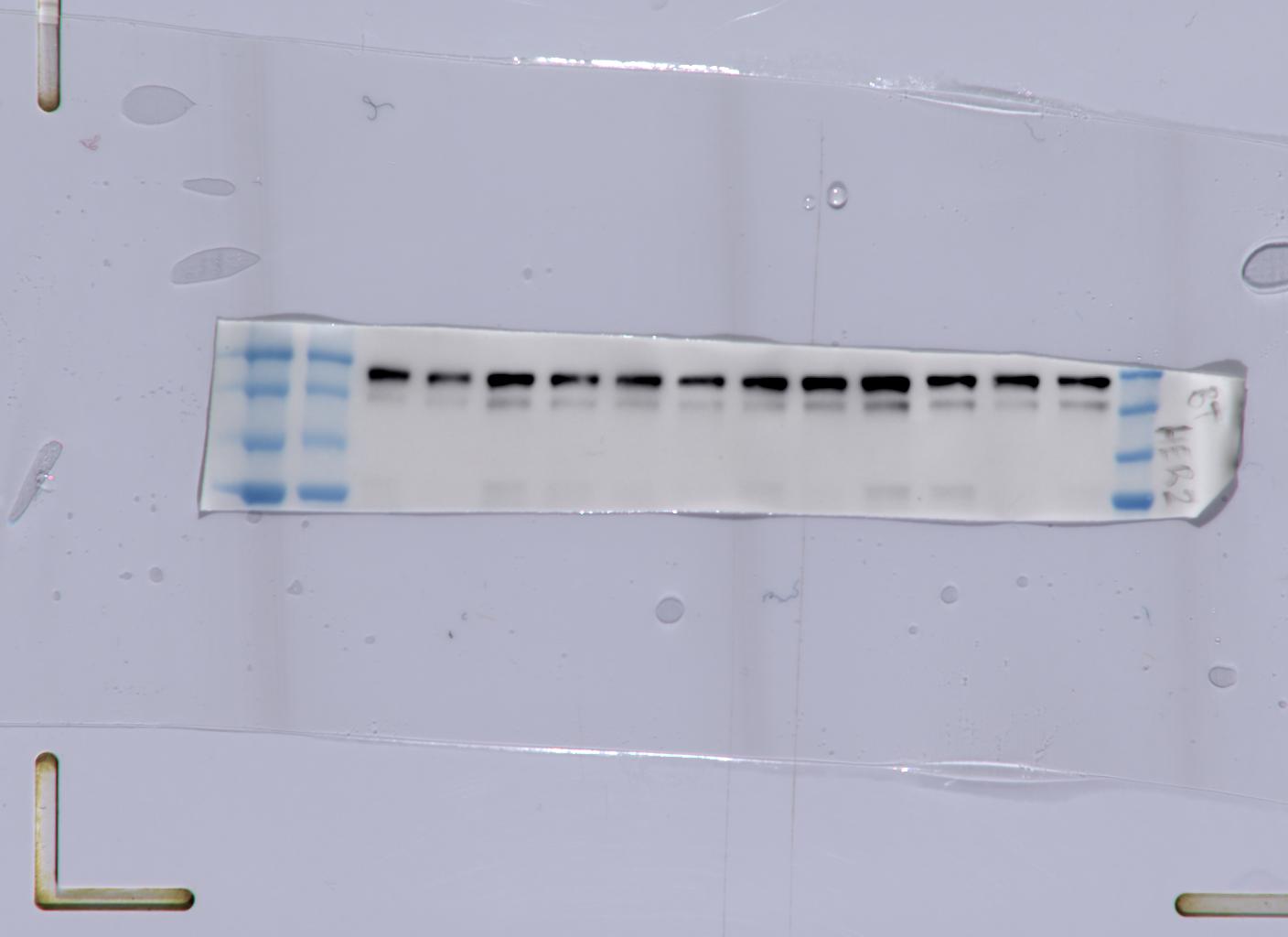

Supplement: Supplementary file 1 [file cancers-13-02778-s001.zip › Figure.S6/Figure5/BT474-BT474.rT3/HER2.jpg]

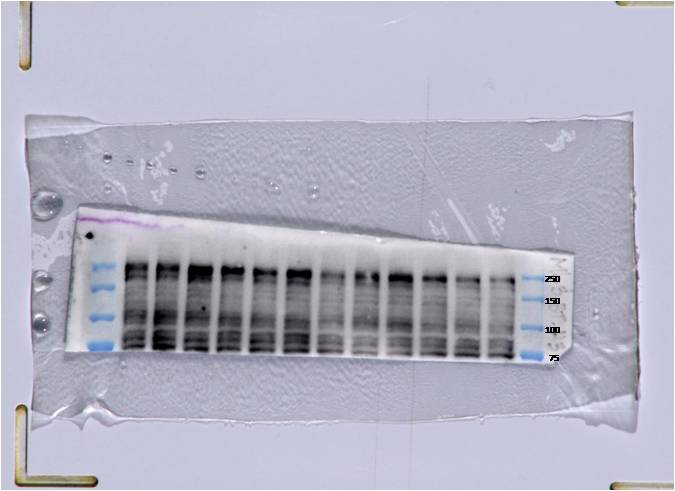

Supplement: Supplementary file 1 [file cancers-13-02778-s001.zip › Figure.S6/Figure5/BT474-BT474.rT3/HER3 MW.jpg]

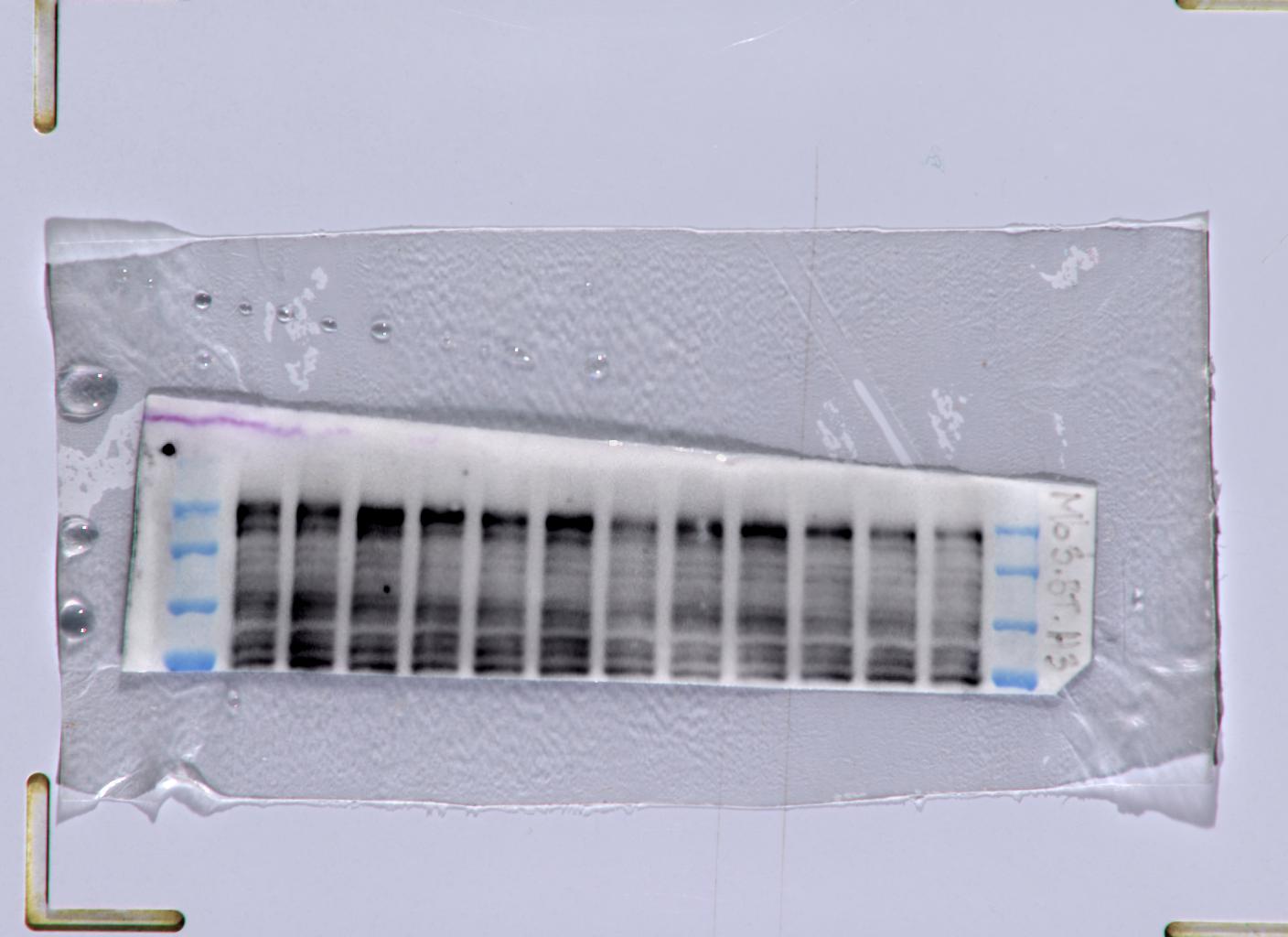

Supplement: Supplementary file 1 [file cancers-13-02778-s001.zip › Figure.S6/Figure5/BT474-BT474.rT3/HER3.jpg]

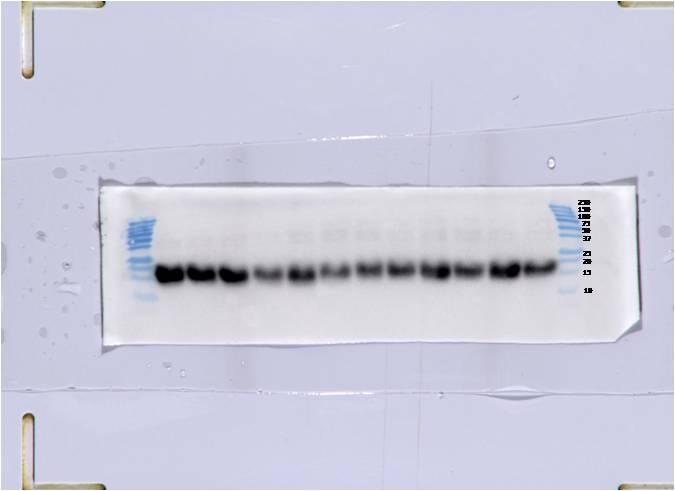

Supplement: Supplementary file 1 [file cancers-13-02778-s001.zip › Figure.S6/Figure5/BT474-BT474.rT3/p4EBP1 T37 46 MW.jpg]

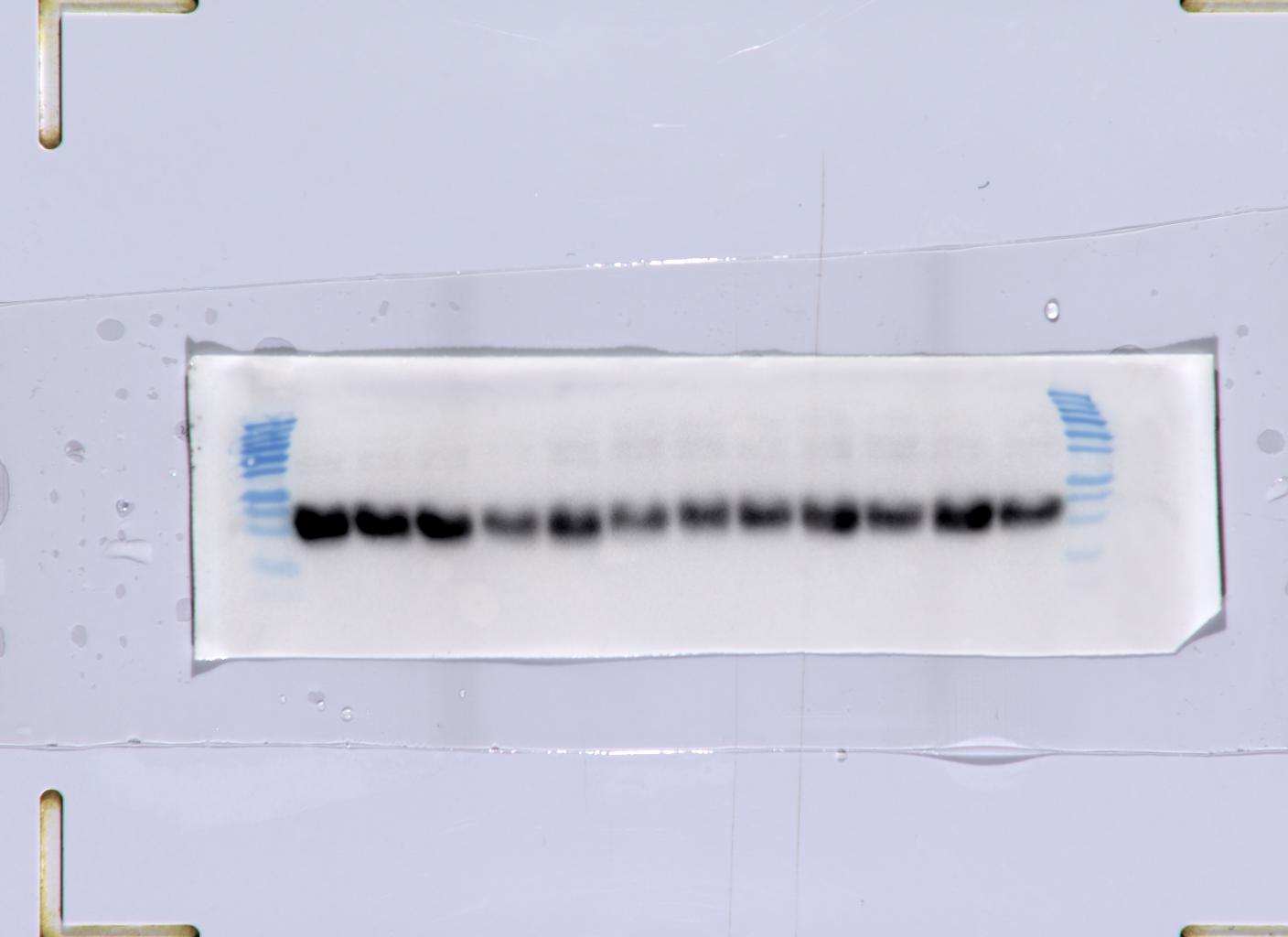

Supplement: Supplementary file 1 [file cancers-13-02778-s001.zip › Figure.S6/Figure5/BT474-BT474.rT3/p4EBP1 T37 46.jpg]

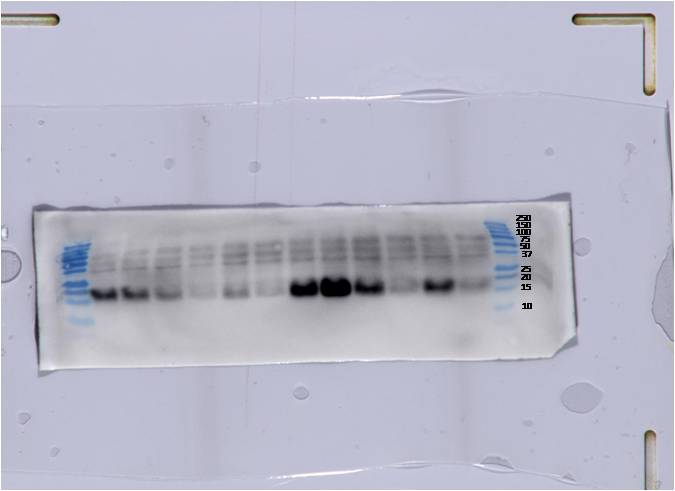

Supplement: Supplementary file 1 [file cancers-13-02778-s001.zip › Figure.S6/Figure5/BT474-BT474.rT3/p4EBP1 T70 MW.jpg]

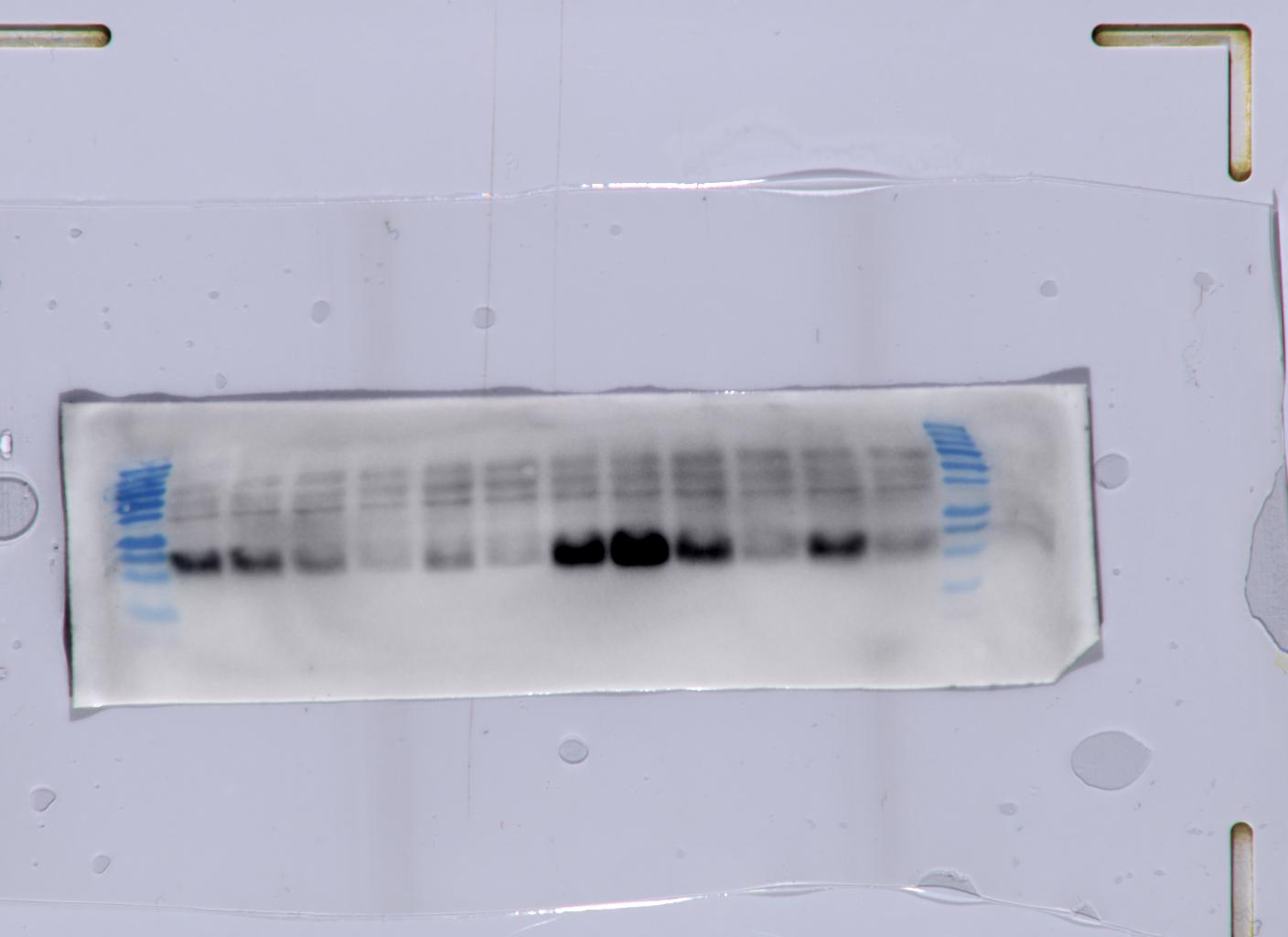

Supplement: Supplementary file 1 [file cancers-13-02778-s001.zip › Figure.S6/Figure5/BT474-BT474.rT3/p4EBP1 T70.jpg]

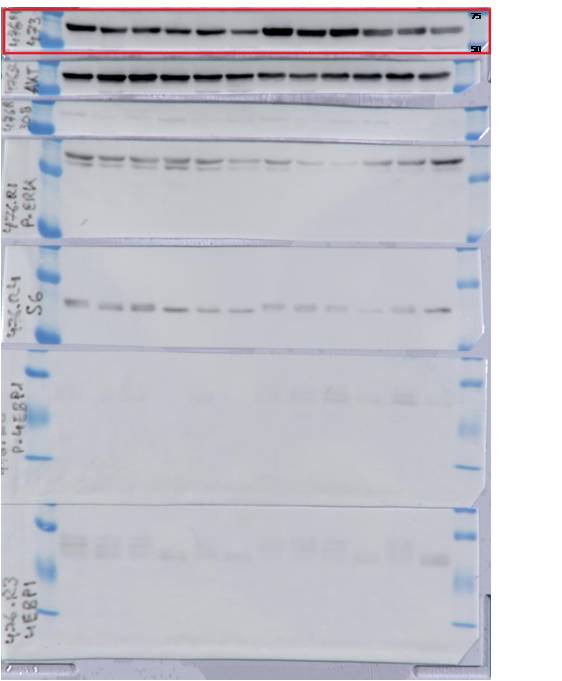

Supplement: Supplementary file 1 [file cancers-13-02778-s001.zip › Figure.S6/Figure5/BT474-BT474.rT3/pAKT 473 MW.jpg]

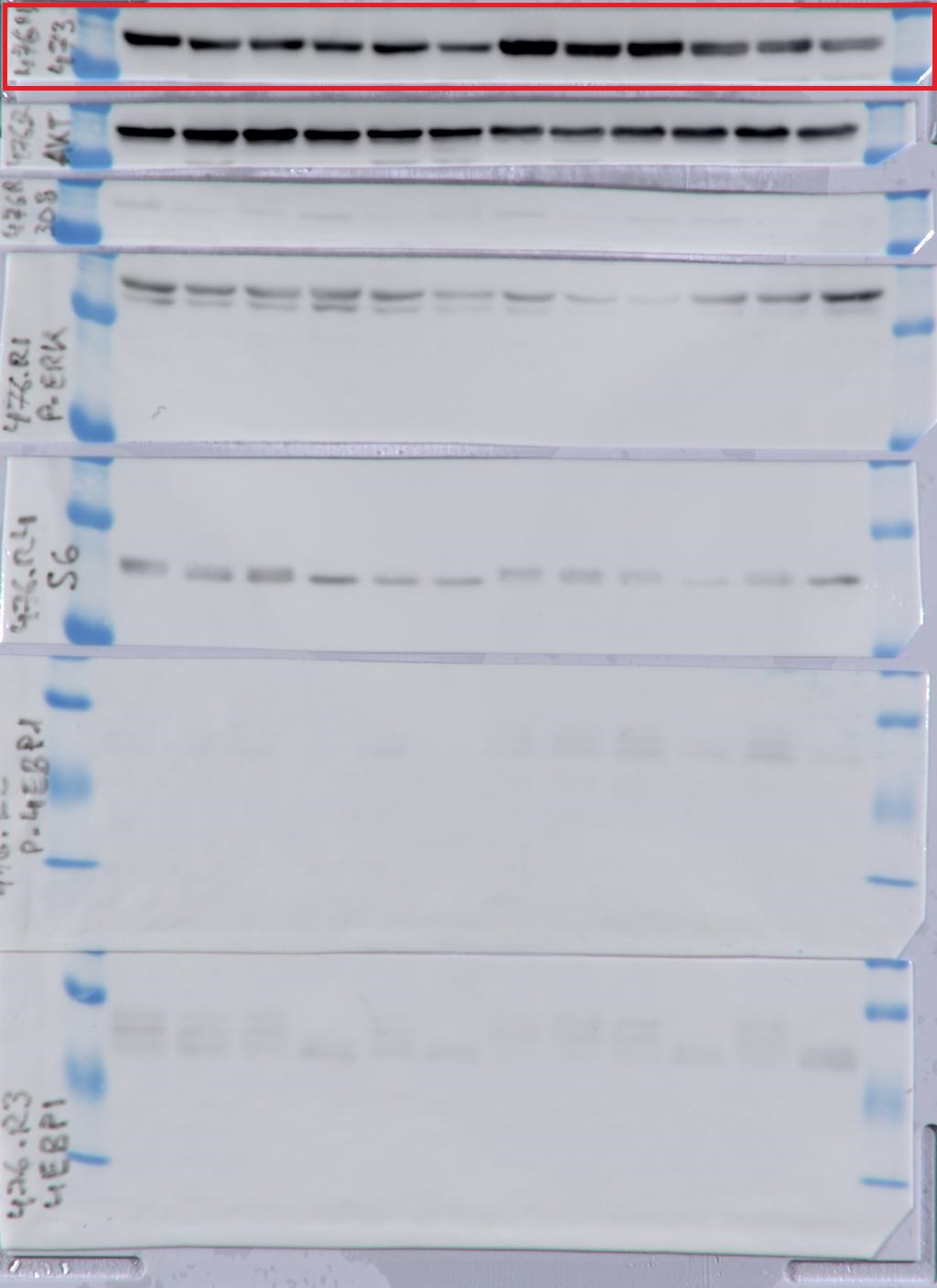

Supplement: Supplementary file 1 [file cancers-13-02778-s001.zip › Figure.S6/Figure5/BT474-BT474.rT3/pAKT 473.jpg]

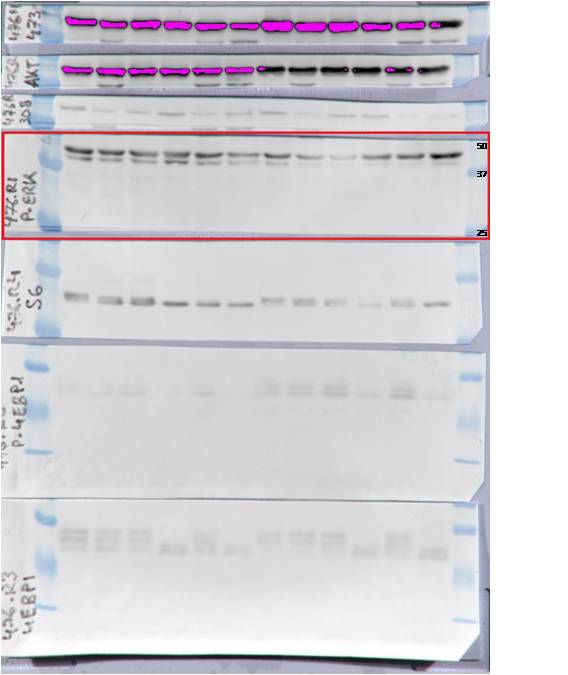

Supplement: Supplementary file 1 [file cancers-13-02778-s001.zip › Figure.S6/Figure5/BT474-BT474.rT3/pERK MW.jpg]

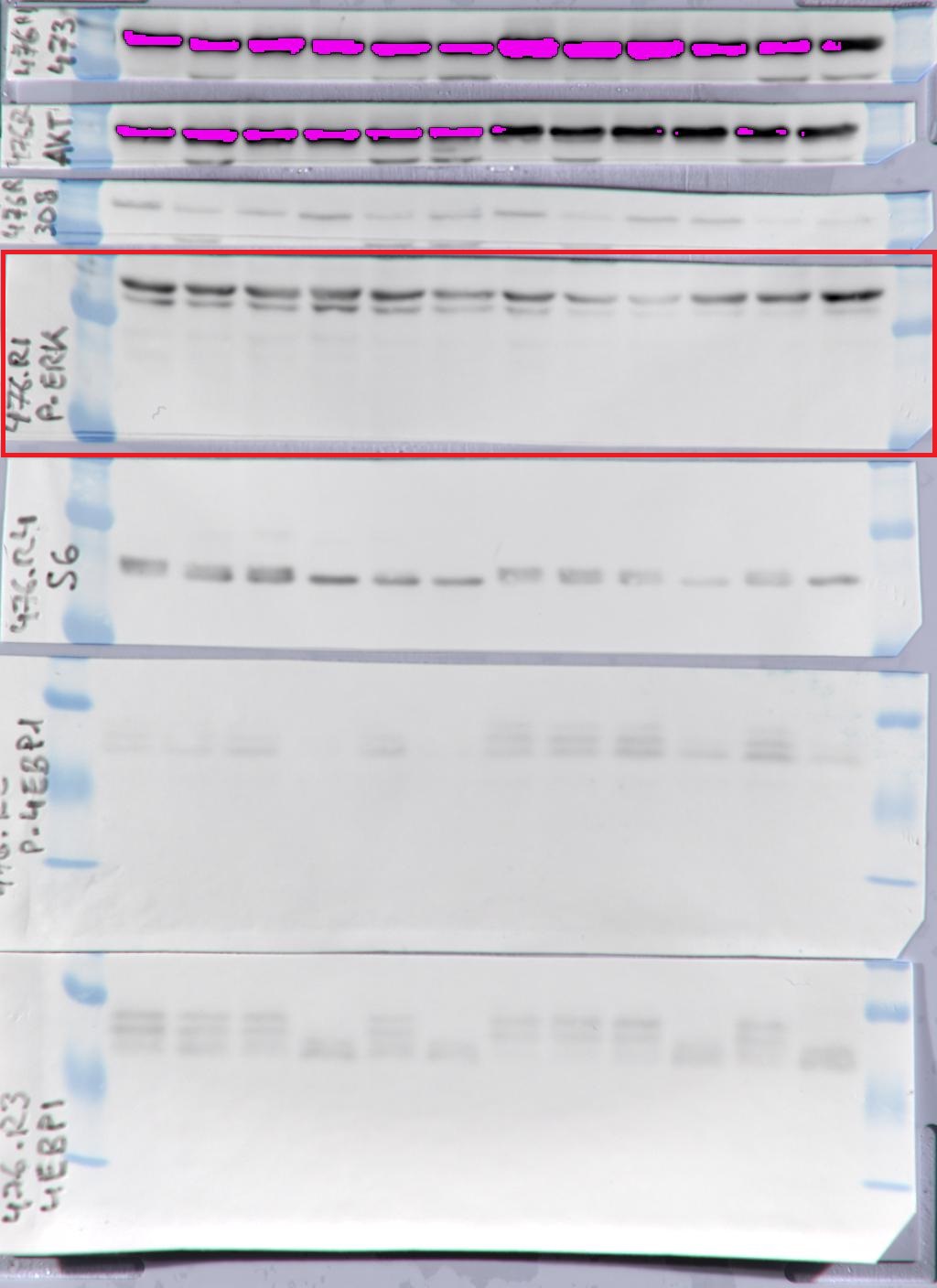

Supplement: Supplementary file 1 [file cancers-13-02778-s001.zip › Figure.S6/Figure5/BT474-BT474.rT3/pERK.jpg]

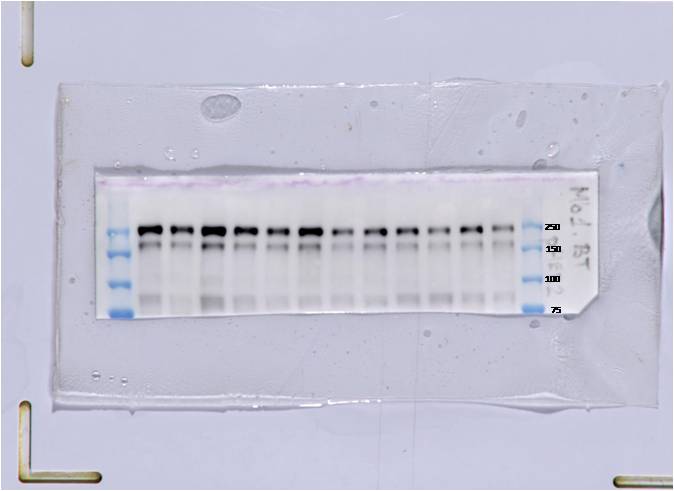

Supplement: Supplementary file 1 [file cancers-13-02778-s001.zip › Figure.S6/Figure5/BT474-BT474.rT3/pHER2 MW.jpg]

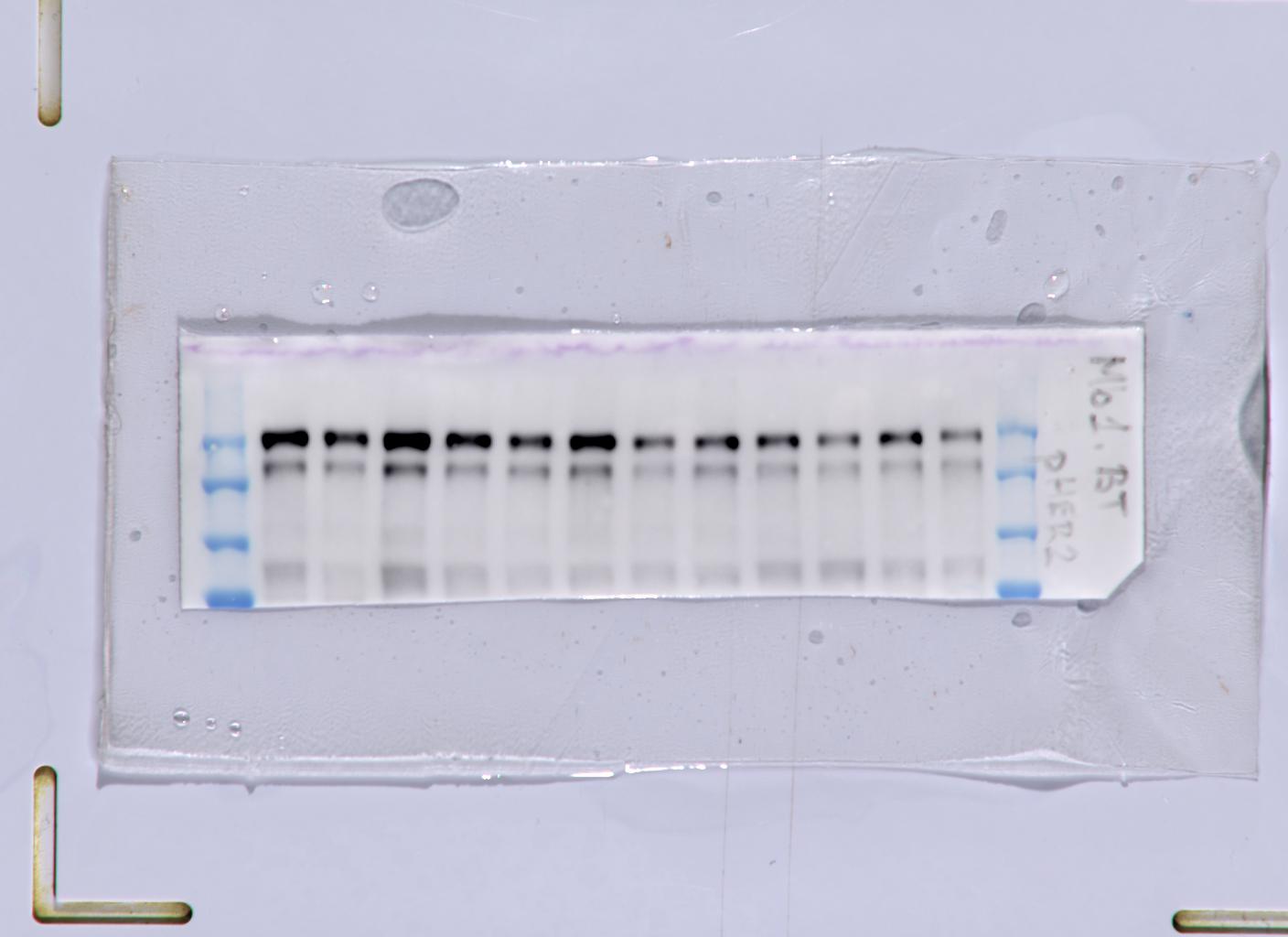

Supplement: Supplementary file 1 [file cancers-13-02778-s001.zip › Figure.S6/Figure5/BT474-BT474.rT3/pHER2.jpg]

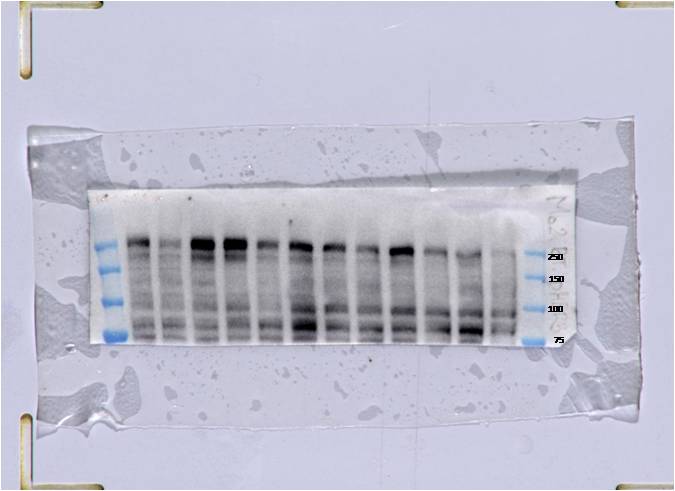

Supplement: Supplementary file 1 [file cancers-13-02778-s001.zip › Figure.S6/Figure5/BT474-BT474.rT3/pHER3 MW.jpg]

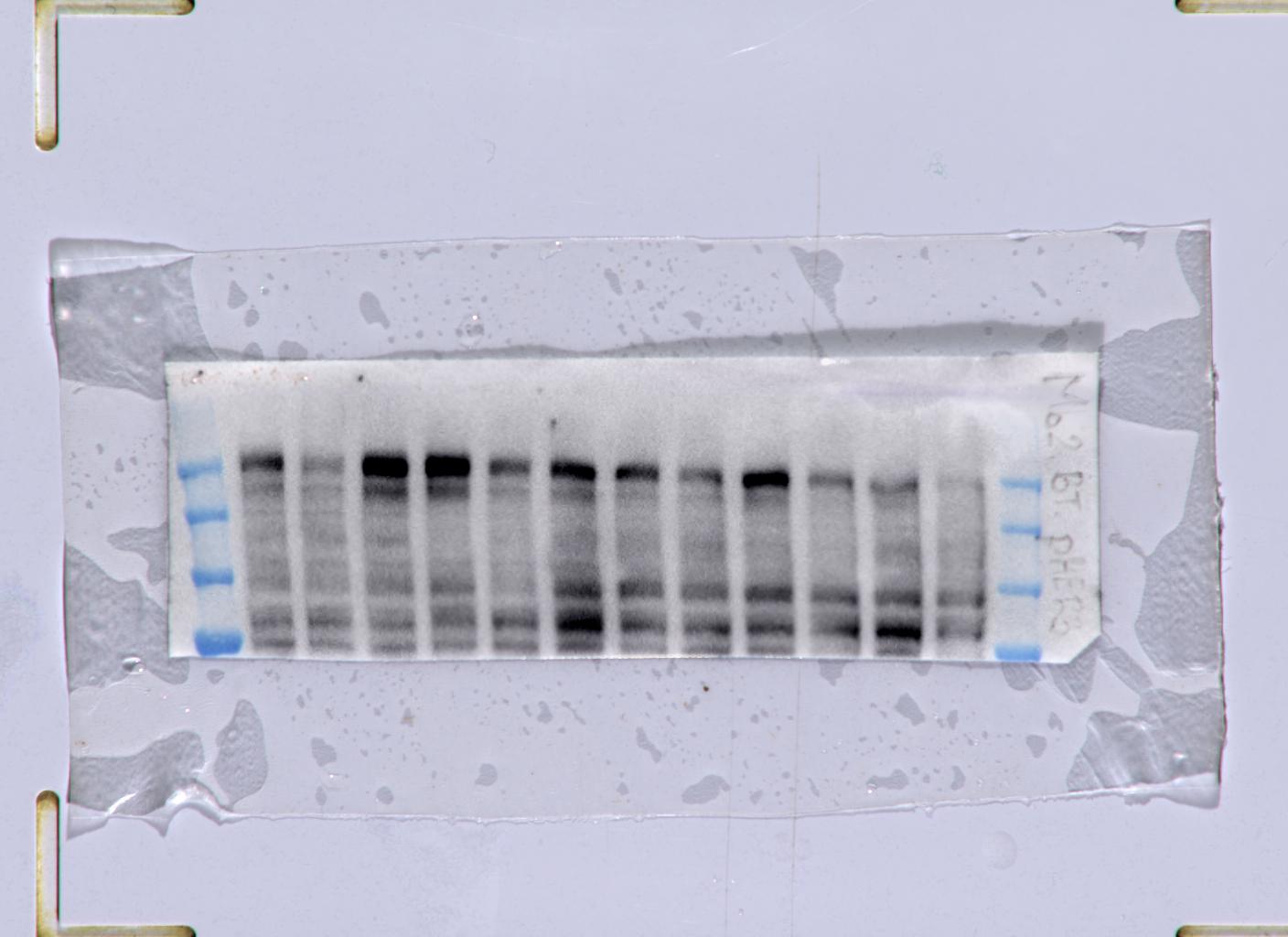

Supplement: Supplementary file 1 [file cancers-13-02778-s001.zip › Figure.S6/Figure5/BT474-BT474.rT3/pHER3.jpg]

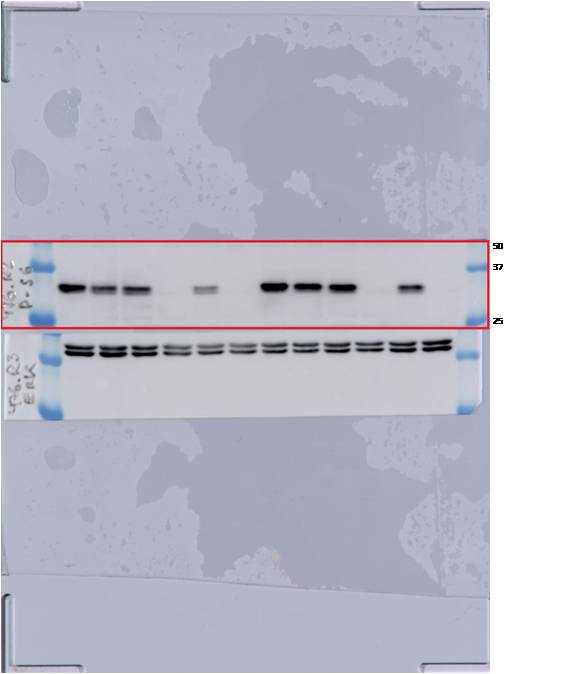

Supplement: Supplementary file 1 [file cancers-13-02778-s001.zip › Figure.S6/Figure5/BT474-BT474.rT3/pS6 MW.jpg]

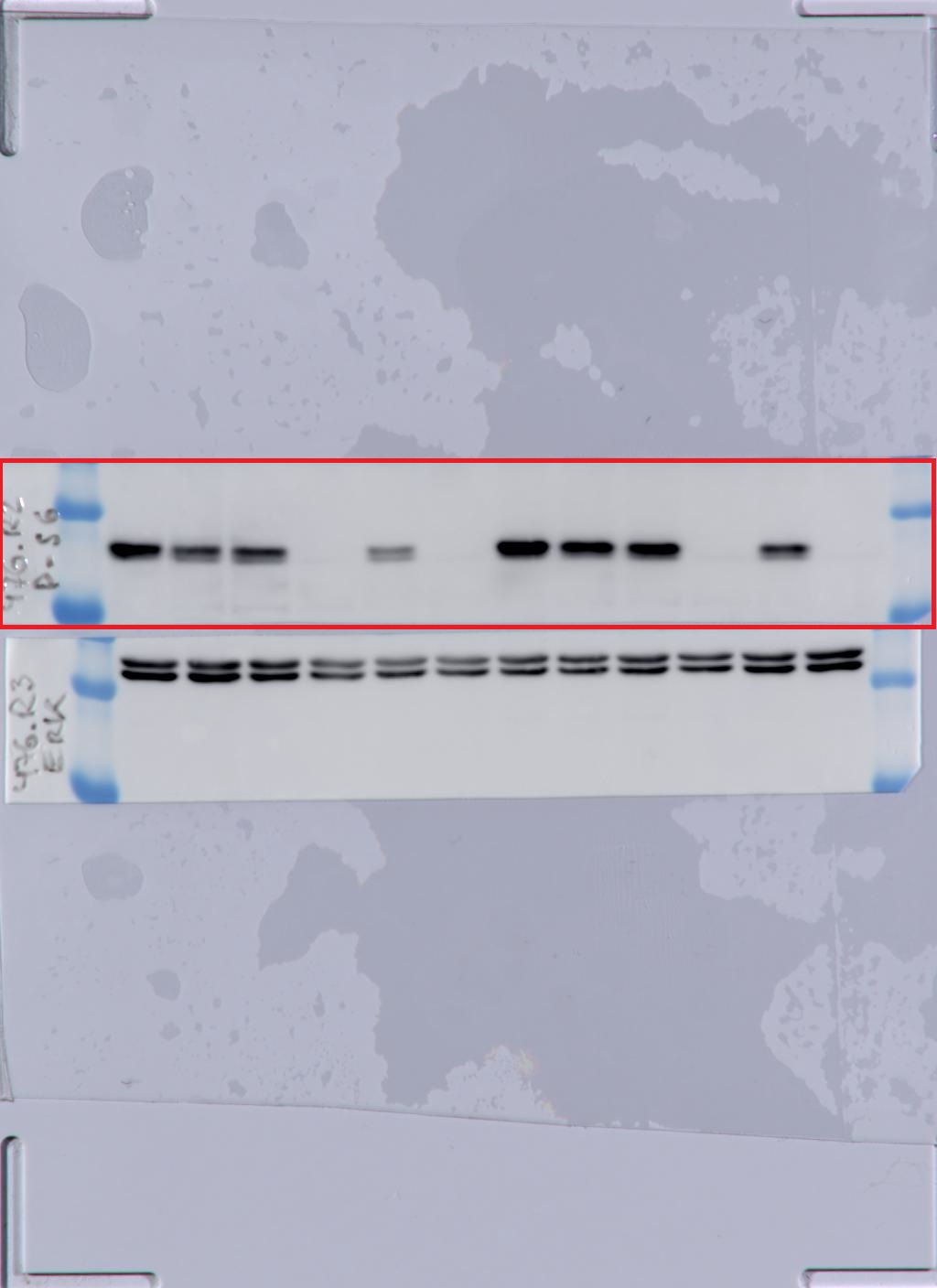

Supplement: Supplementary file 1 [file cancers-13-02778-s001.zip › Figure.S6/Figure5/BT474-BT474.rT3/pS6.jpg]

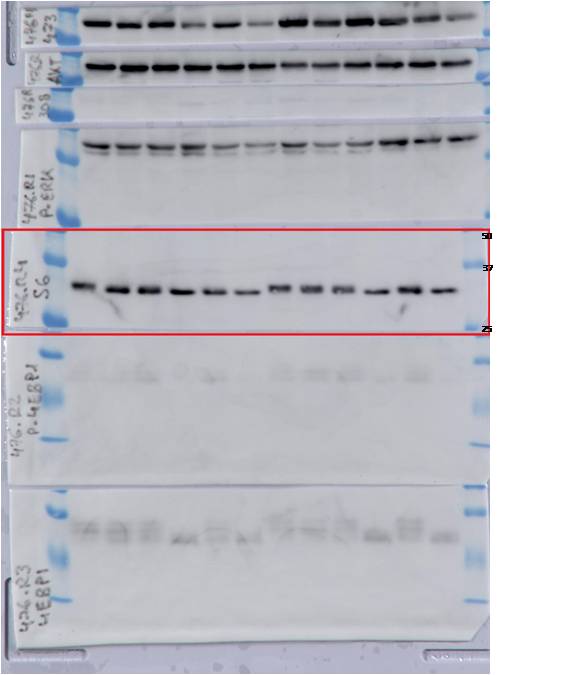

Supplement: Supplementary file 1 [file cancers-13-02778-s001.zip › Figure.S6/Figure5/BT474-BT474.rT3/S6 MW.jpg]

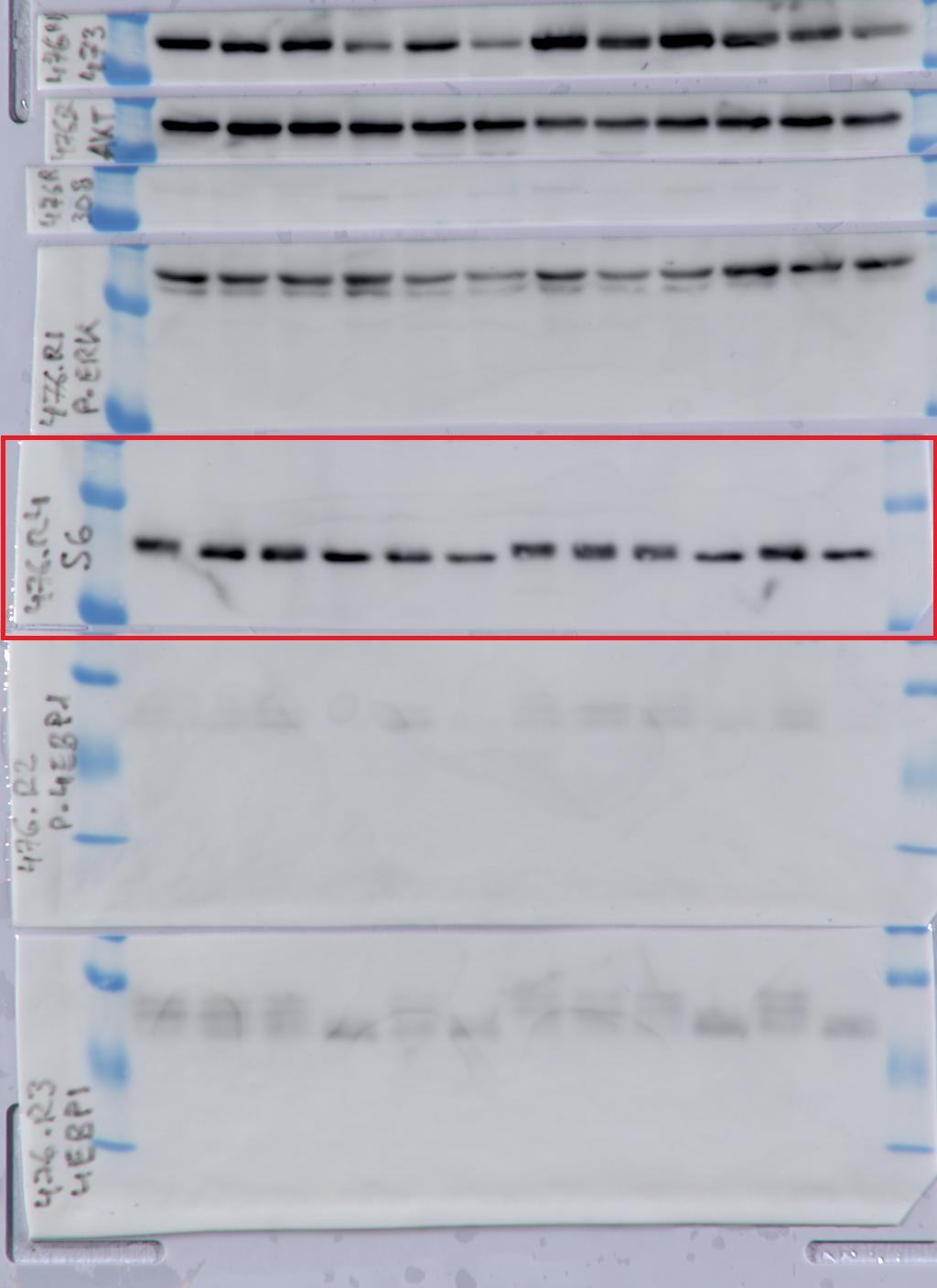

Supplement: Supplementary file 1 [file cancers-13-02778-s001.zip › Figure.S6/Figure5/BT474-BT474.rT3/S6.jpg]

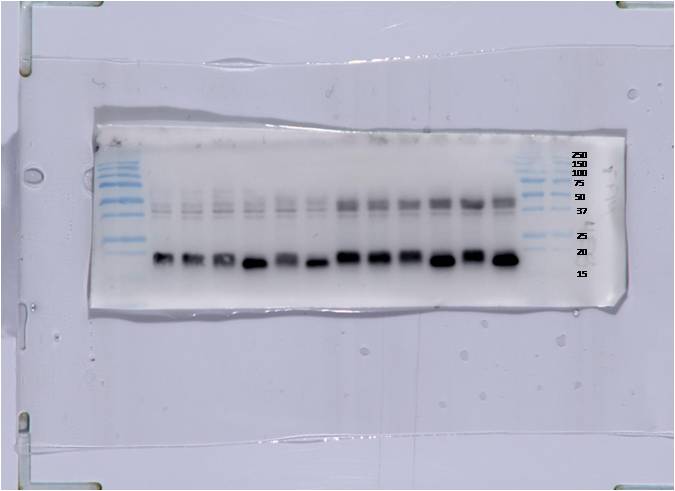

Supplement: Supplementary file 1 [file cancers-13-02778-s001.zip › Figure.S6/Figure5/EFM192A-EFM192A.rT1/4EBP1 MW.jpg]

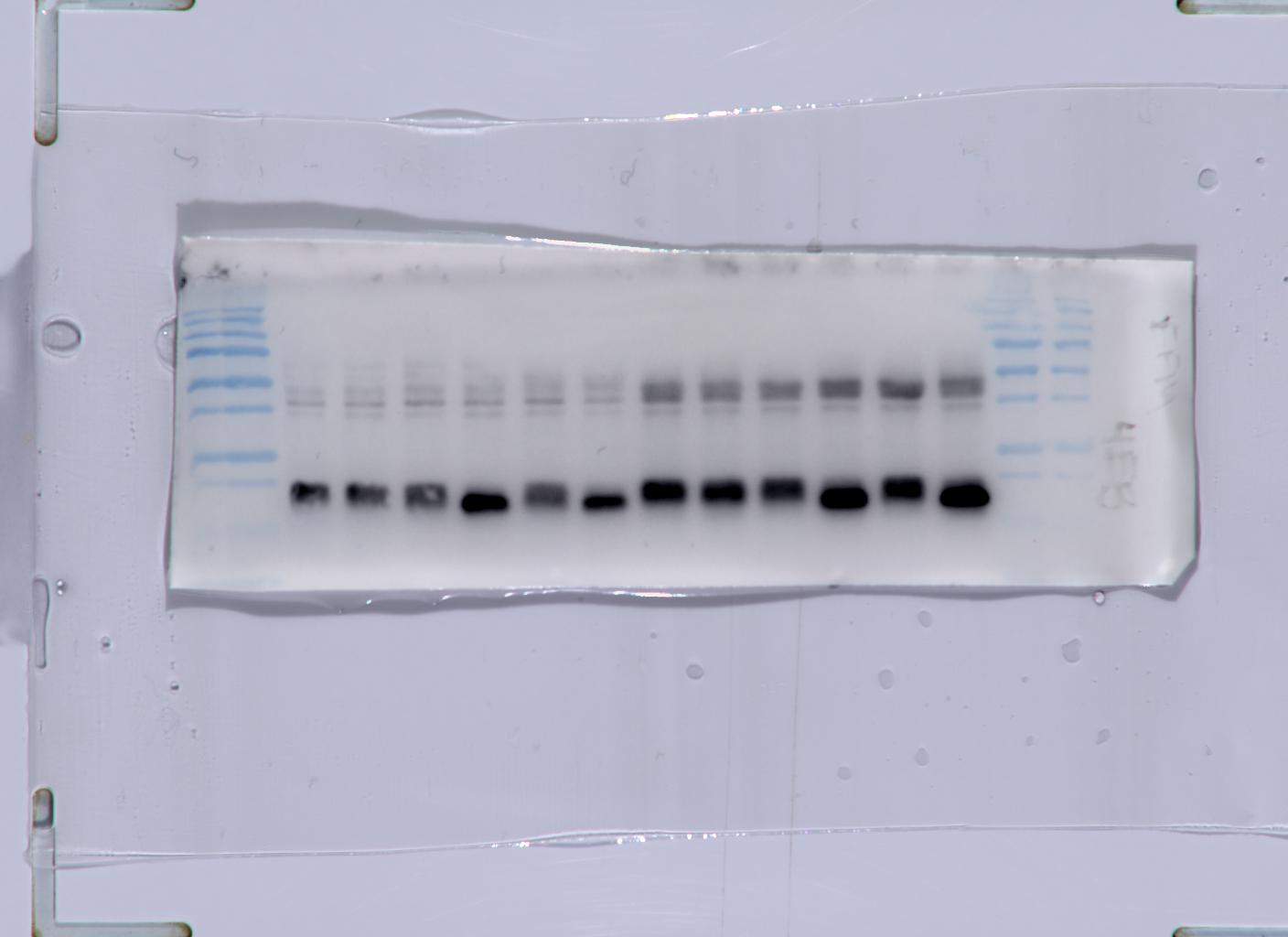

Supplement: Supplementary file 1 [file cancers-13-02778-s001.zip › Figure.S6/Figure5/EFM192A-EFM192A.rT1/4EBP1.jpg]

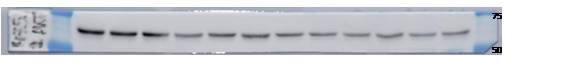

Supplement: Supplementary file 1 [file cancers-13-02778-s001.zip › Figure.S6/Figure5/EFM192A-EFM192A.rT1/AKT MW.jpg]

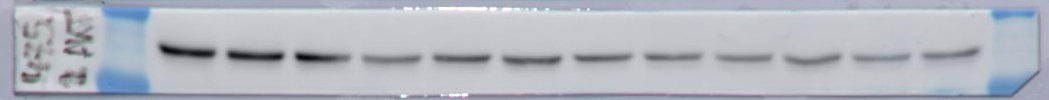

Supplement: Supplementary file 1 [file cancers-13-02778-s001.zip › Figure.S6/Figure5/EFM192A-EFM192A.rT1/AKT.jpg]

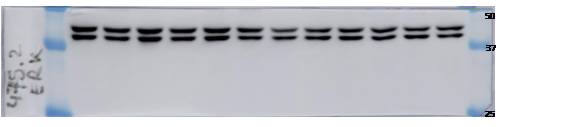

Supplement: Supplementary file 1 [file cancers-13-02778-s001.zip › Figure.S6/Figure5/EFM192A-EFM192A.rT1/ERK MW.jpg]

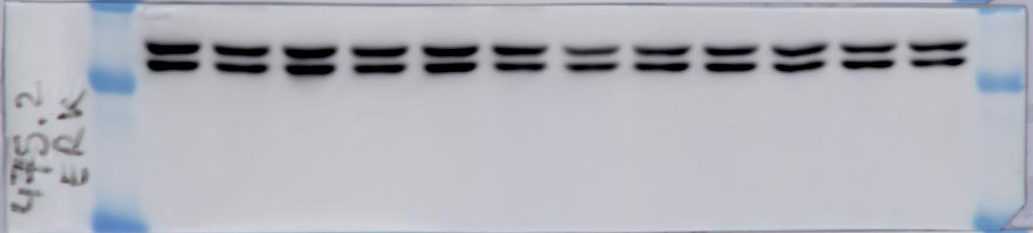

Supplement: Supplementary file 1 [file cancers-13-02778-s001.zip › Figure.S6/Figure5/EFM192A-EFM192A.rT1/ERK.jpg]

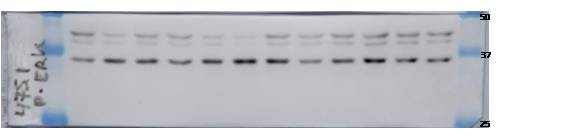

Supplement: Supplementary file 1 [file cancers-13-02778-s001.zip › Figure.S6/Figure5/EFM192A-EFM192A.rT1/GAPDH MW.jpg]

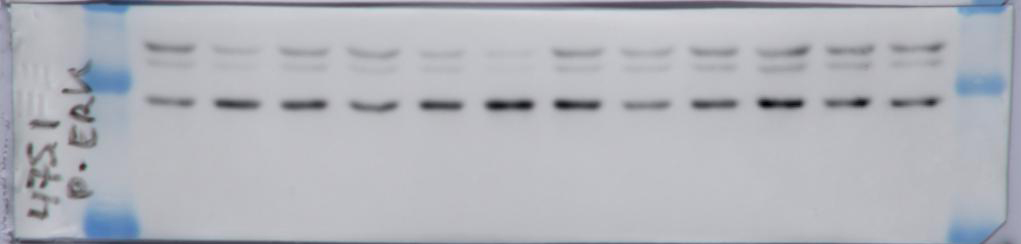

Supplement: Supplementary file 1 [file cancers-13-02778-s001.zip › Figure.S6/Figure5/EFM192A-EFM192A.rT1/GAPDH.jpg]

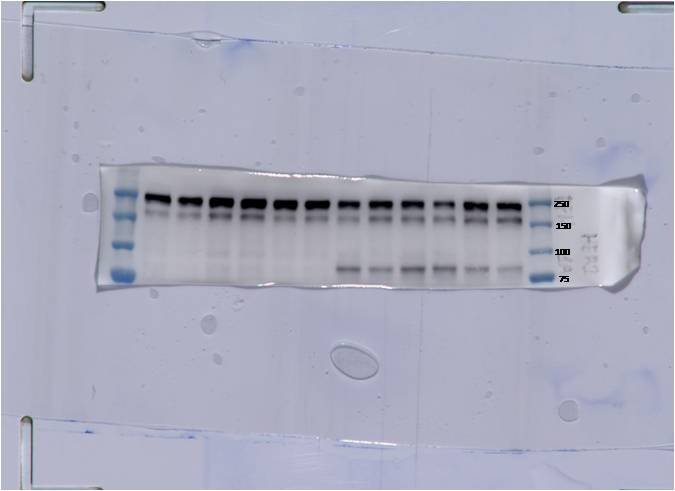

Supplement: Supplementary file 1 [file cancers-13-02778-s001.zip › Figure.S6/Figure5/EFM192A-EFM192A.rT1/HER2 MW.jpg]

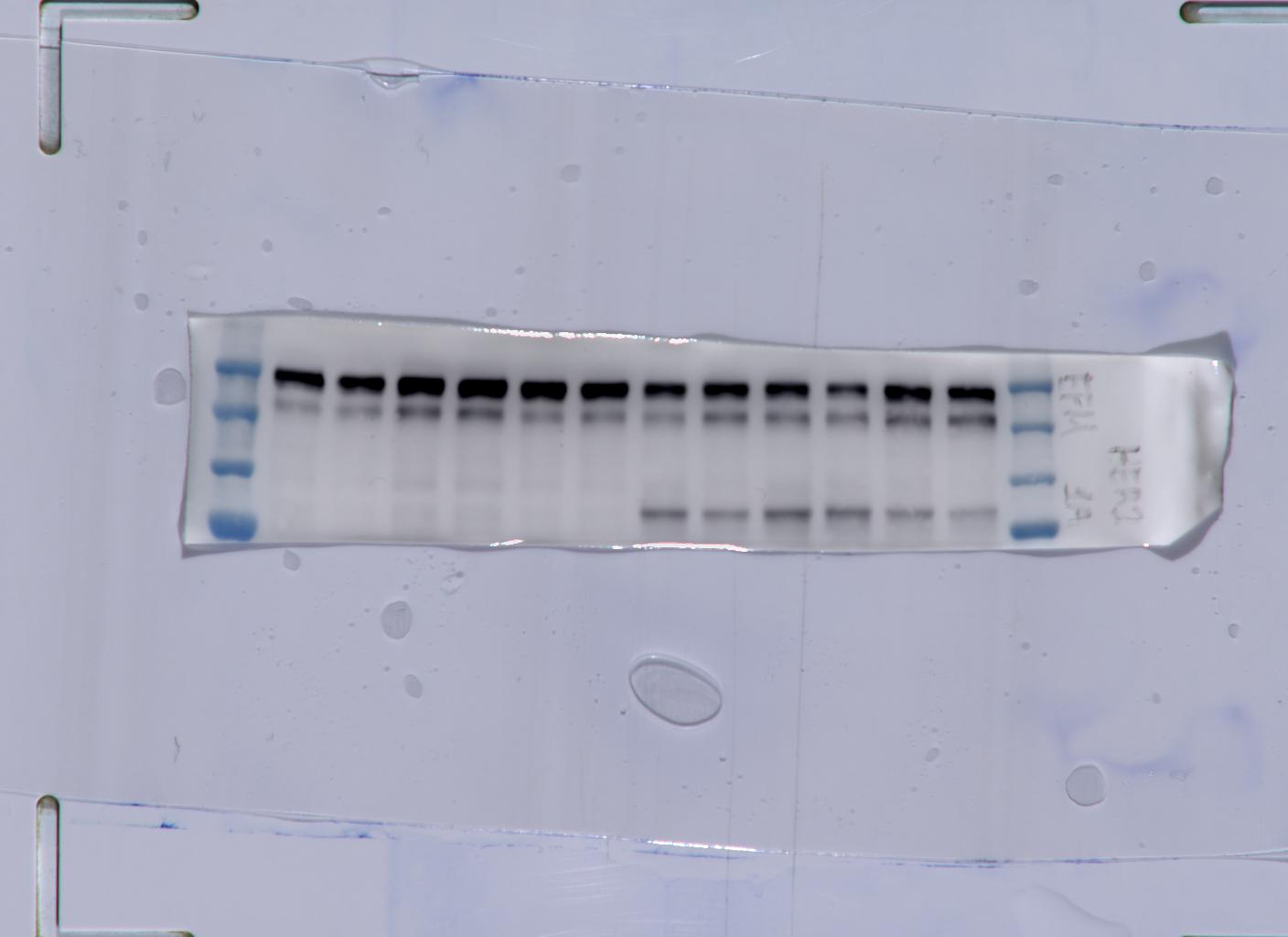

Supplement: Supplementary file 1 [file cancers-13-02778-s001.zip › Figure.S6/Figure5/EFM192A-EFM192A.rT1/HER2.jpg]

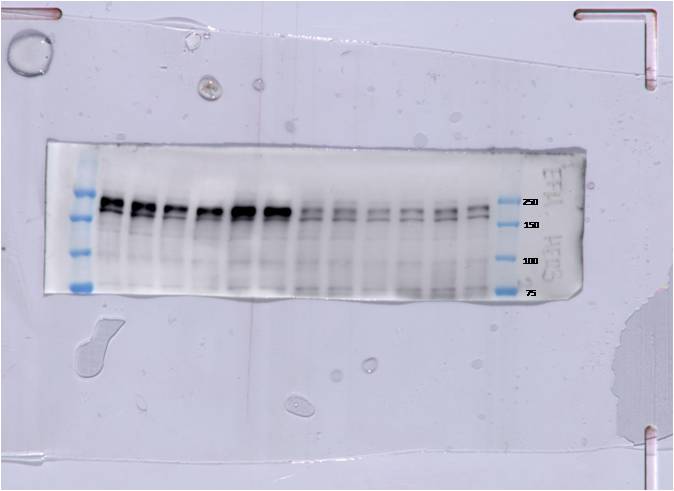

Supplement: Supplementary file 1 [file cancers-13-02778-s001.zip › Figure.S6/Figure5/EFM192A-EFM192A.rT1/HER3 MW.jpg]

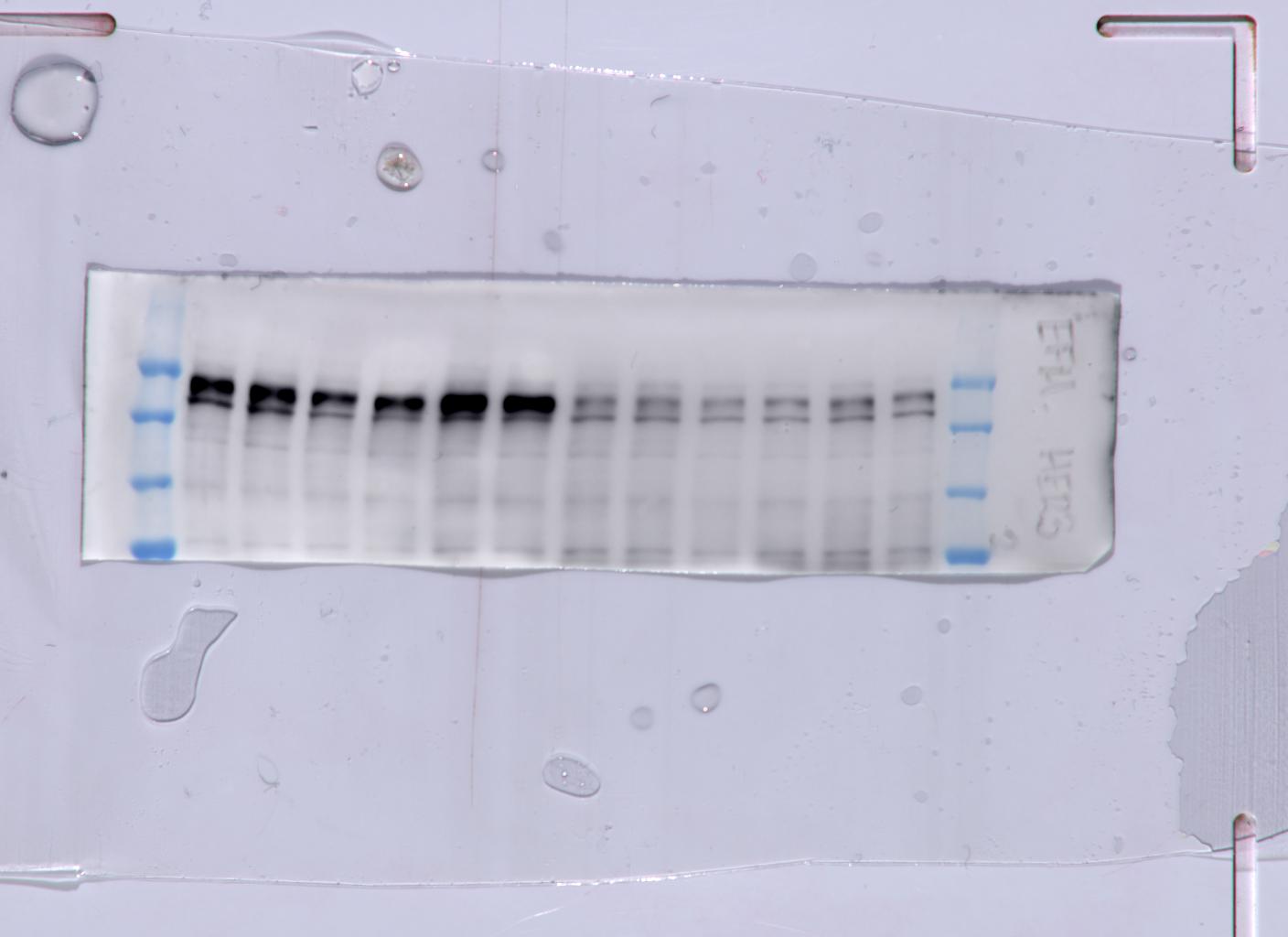

Supplement: Supplementary file 1 [file cancers-13-02778-s001.zip › Figure.S6/Figure5/EFM192A-EFM192A.rT1/HER3.jpg]

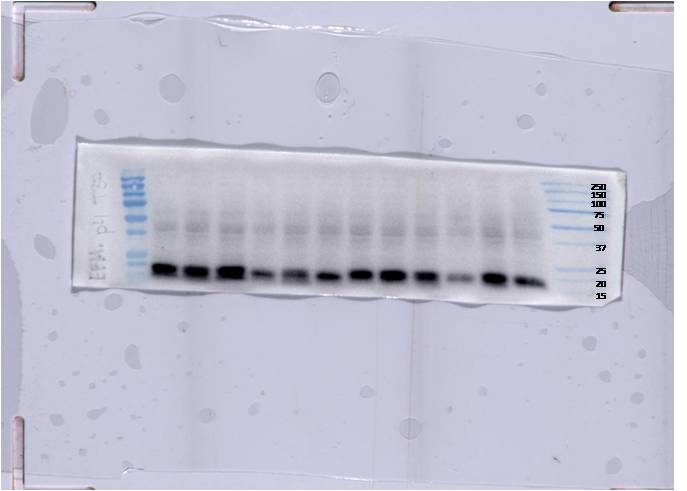

Supplement: Supplementary file 1 [file cancers-13-02778-s001.zip › Figure.S6/Figure5/EFM192A-EFM192A.rT1/p4EBP1 T37 46 MW.jpg]

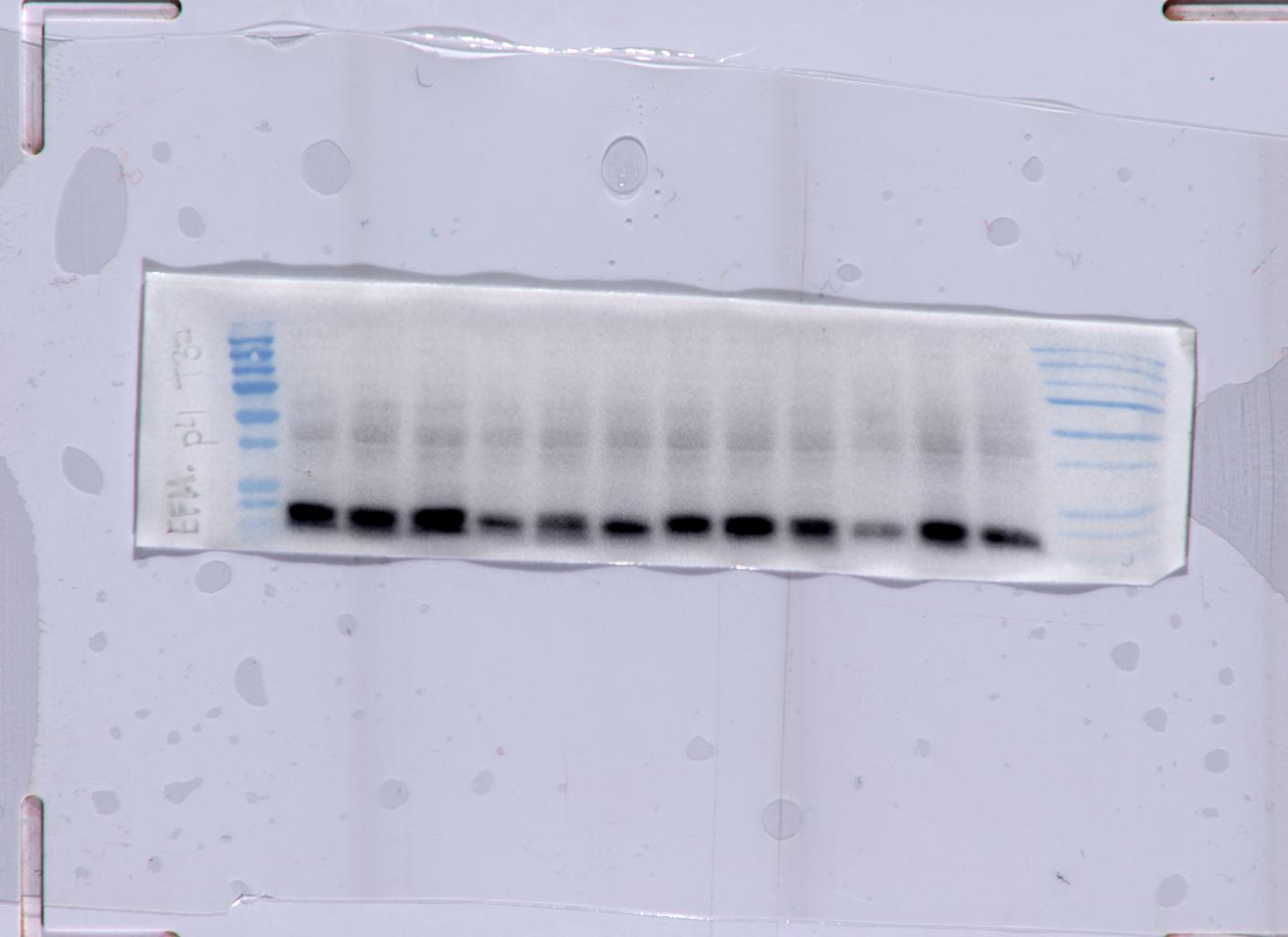

Supplement: Supplementary file 1 [file cancers-13-02778-s001.zip › Figure.S6/Figure5/EFM192A-EFM192A.rT1/p4EBP1 T37 46.jpg]

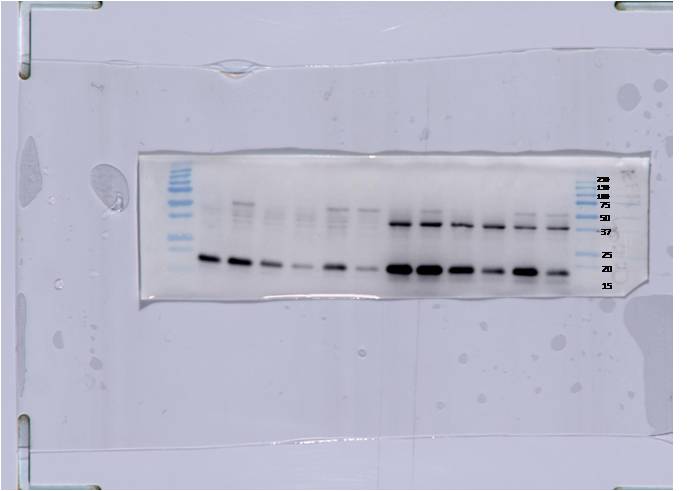

Supplement: Supplementary file 1 [file cancers-13-02778-s001.zip › Figure.S6/Figure5/EFM192A-EFM192A.rT1/p4EBP1 T70 MW.jpg]

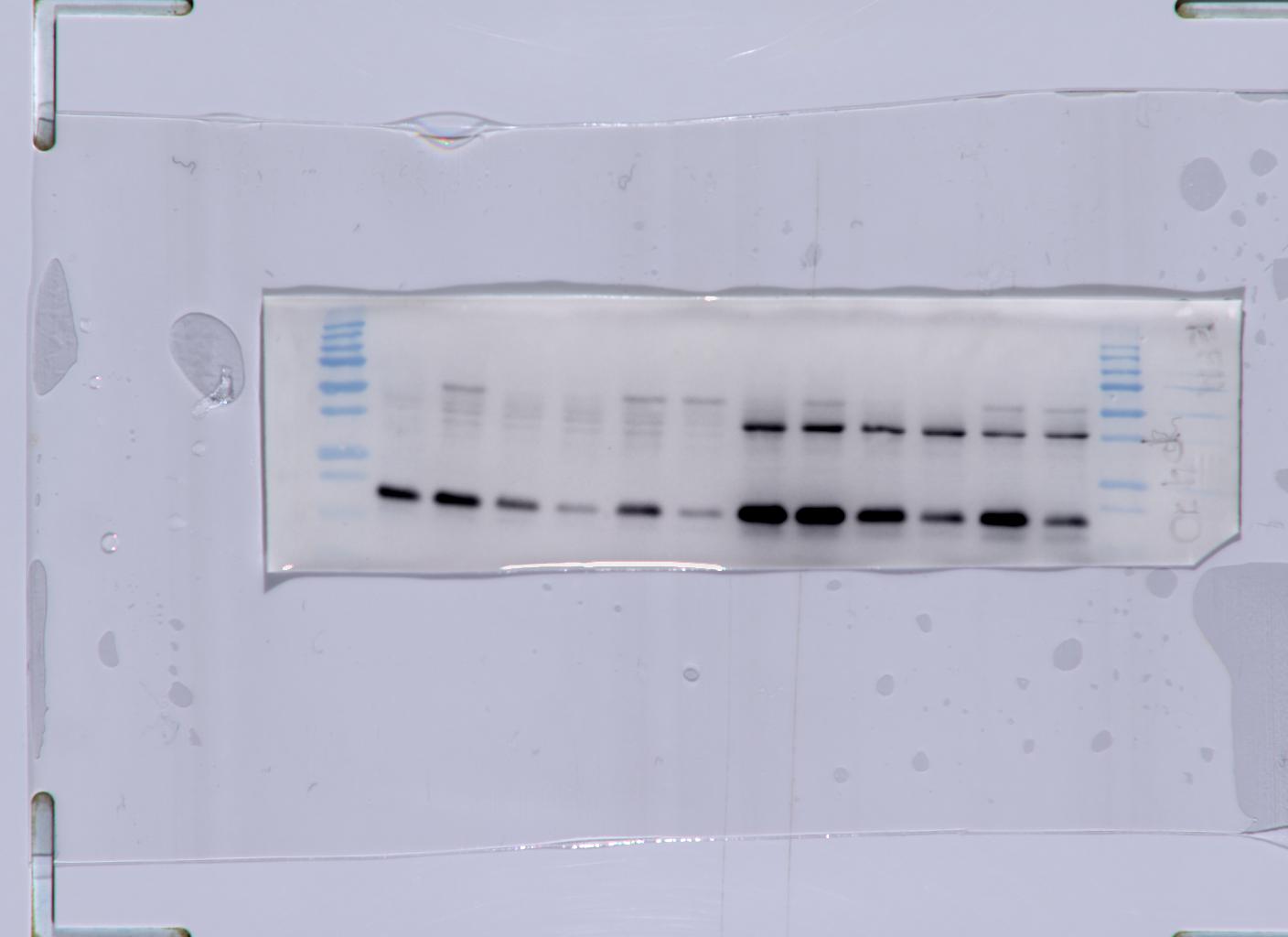

Supplement: Supplementary file 1 [file cancers-13-02778-s001.zip › Figure.S6/Figure5/EFM192A-EFM192A.rT1/p4EBP1 T70.jpg]

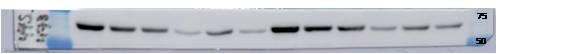

Supplement: Supplementary file 1 [file cancers-13-02778-s001.zip › Figure.S6/Figure5/EFM192A-EFM192A.rT1/pAKT 473 MW.jpg]

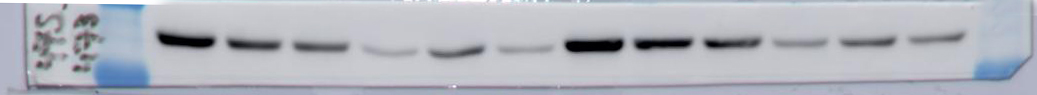

Supplement: Supplementary file 1 [file cancers-13-02778-s001.zip › Figure.S6/Figure5/EFM192A-EFM192A.rT1/pAKT 473.jpg]

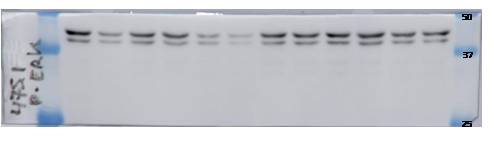

Supplement: Supplementary file 1 [file cancers-13-02778-s001.zip › Figure.S6/Figure5/EFM192A-EFM192A.rT1/pERK MW.jpg]

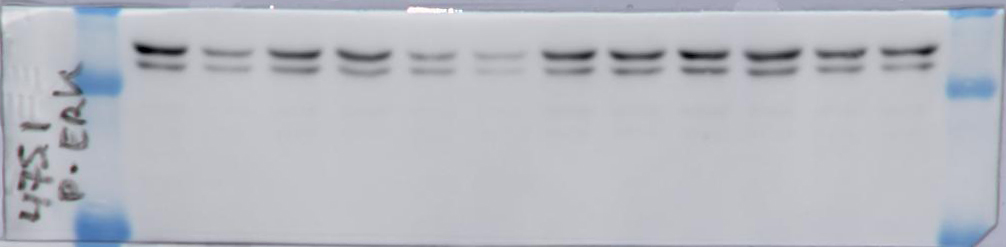

Supplement: Supplementary file 1 [file cancers-13-02778-s001.zip › Figure.S6/Figure5/EFM192A-EFM192A.rT1/pERK.jpg]

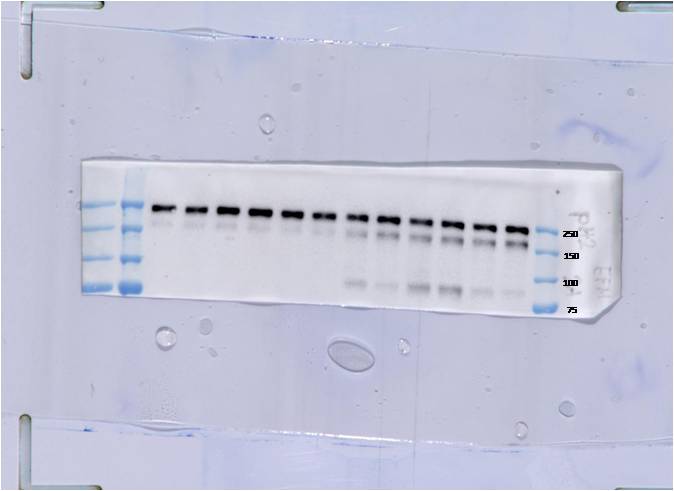

Supplement: Supplementary file 1 [file cancers-13-02778-s001.zip › Figure.S6/Figure5/EFM192A-EFM192A.rT1/pHER2 MW.jpg]

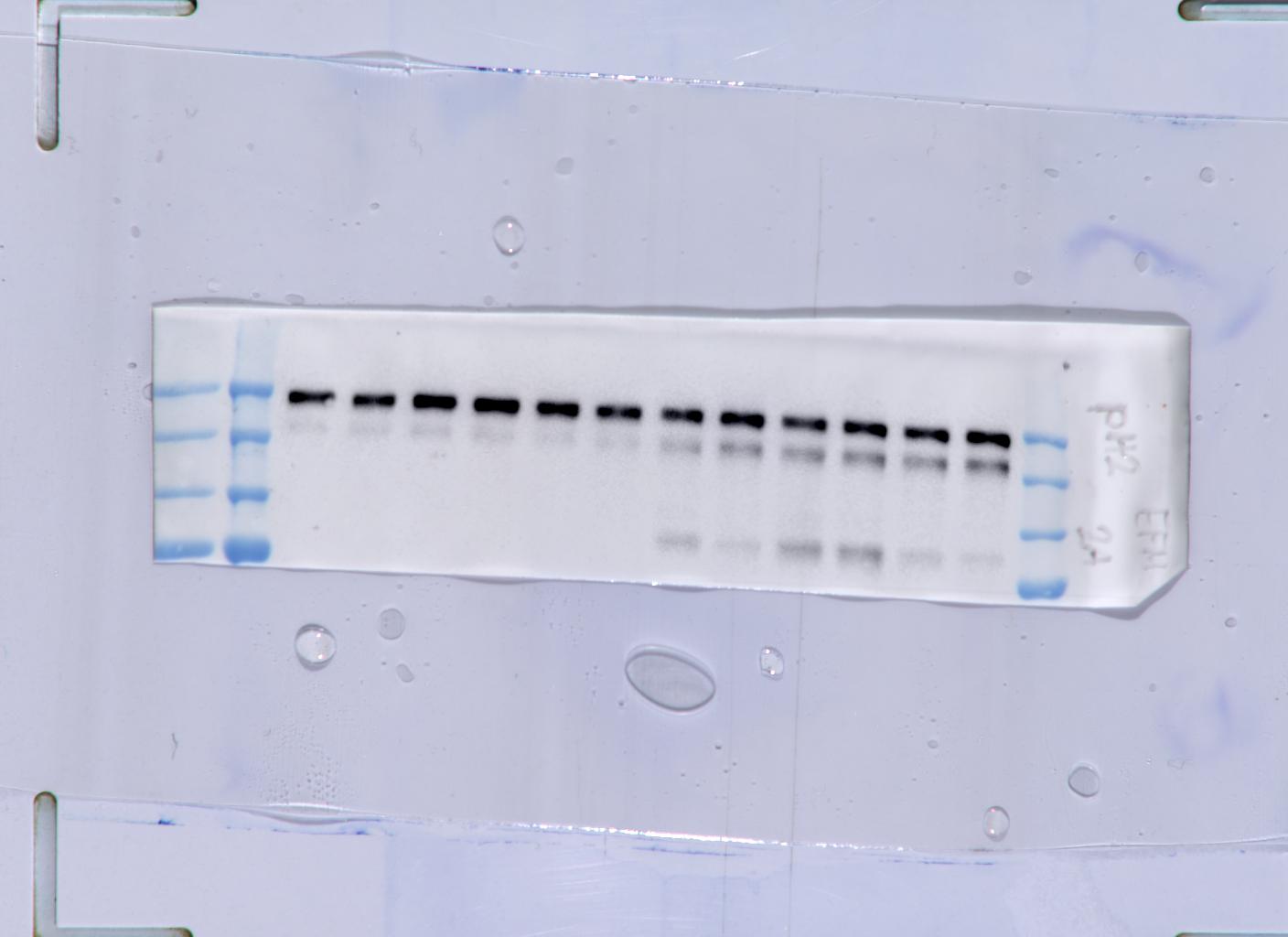

Supplement: Supplementary file 1 [file cancers-13-02778-s001.zip › Figure.S6/Figure5/EFM192A-EFM192A.rT1/pHER2.jpg]

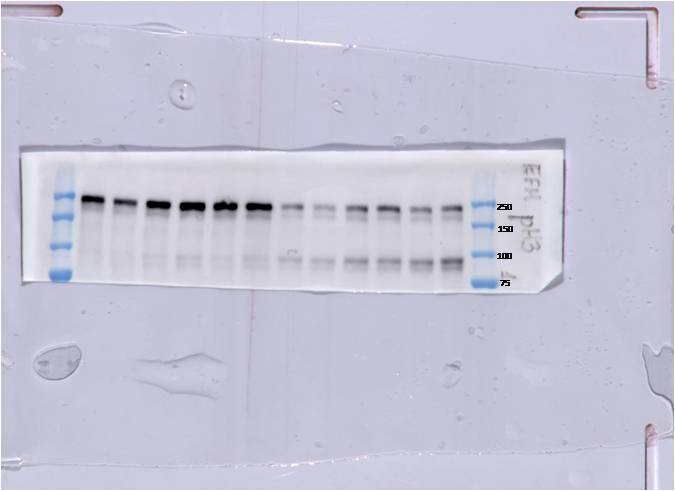

Supplement: Supplementary file 1 [file cancers-13-02778-s001.zip › Figure.S6/Figure5/EFM192A-EFM192A.rT1/pHER3 MW.jpg]

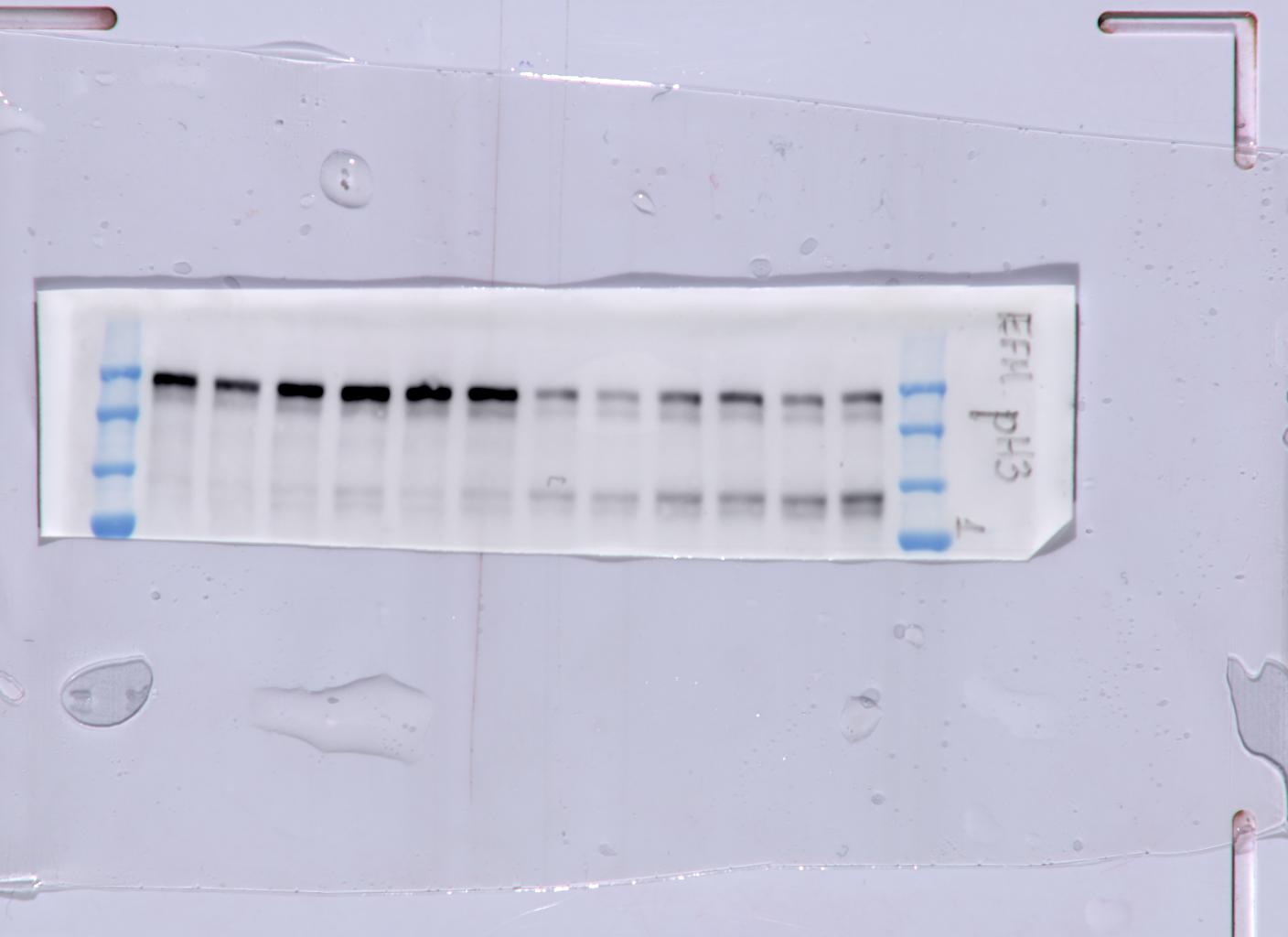

Supplement: Supplementary file 1 [file cancers-13-02778-s001.zip › Figure.S6/Figure5/EFM192A-EFM192A.rT1/pHER3.jpg]

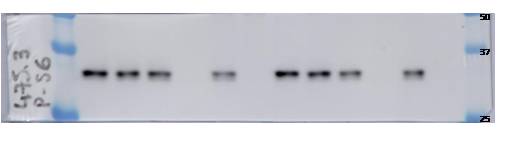

Supplement: Supplementary file 1 [file cancers-13-02778-s001.zip › Figure.S6/Figure5/EFM192A-EFM192A.rT1/pS6 MW.jpg]

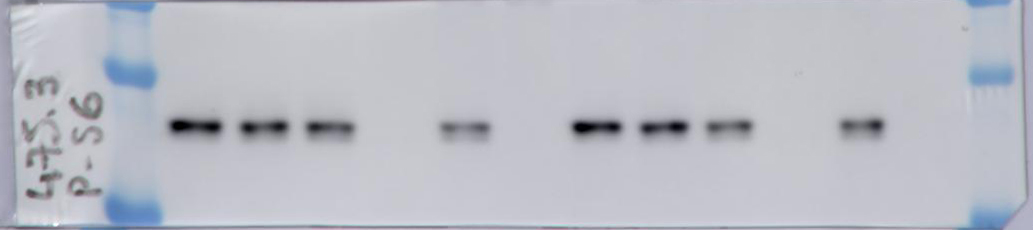

Supplement: Supplementary file 1 [file cancers-13-02778-s001.zip › Figure.S6/Figure5/EFM192A-EFM192A.rT1/pS6.jpg]

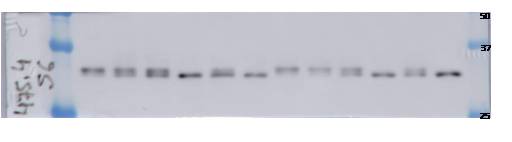

Supplement: Supplementary file 1 [file cancers-13-02778-s001.zip › Figure.S6/Figure5/EFM192A-EFM192A.rT1/S6 MW.jpg]

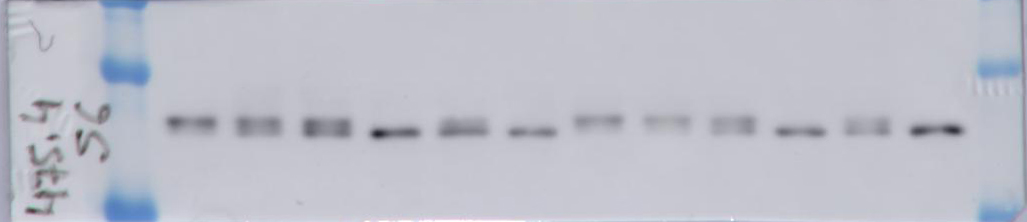

Supplement: Supplementary file 1 [file cancers-13-02778-s001.zip › Figure.S6/Figure5/EFM192A-EFM192A.rT1/S6.jpg]

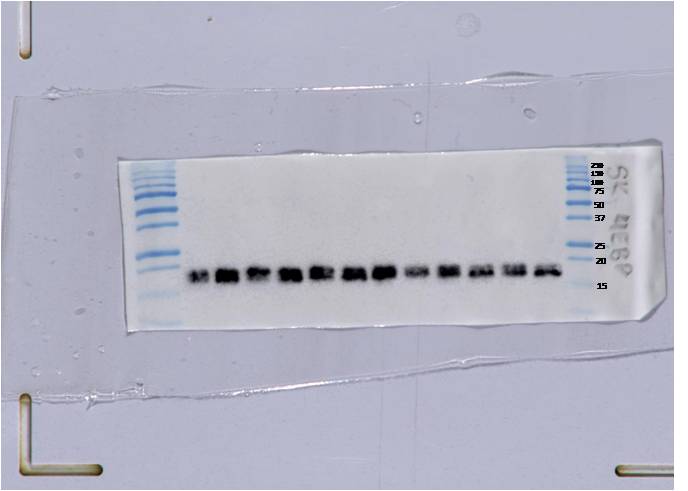

Supplement: Supplementary file 1 [file cancers-13-02778-s001.zip › Figure.S6/Figure5/SKBR3-SKBR3.rT1/4EBP1 MW.jpg]

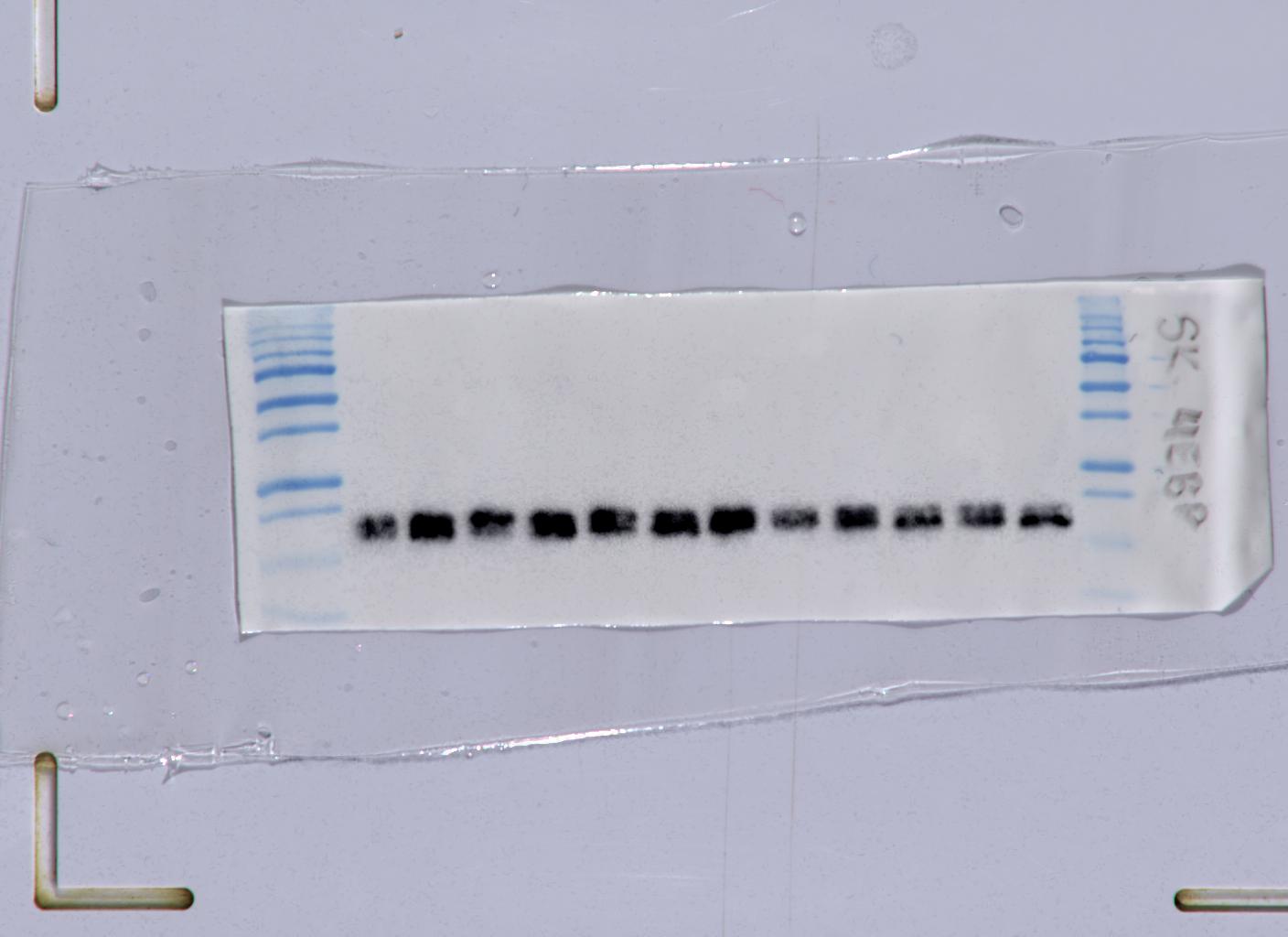

Supplement: Supplementary file 1 [file cancers-13-02778-s001.zip › Figure.S6/Figure5/SKBR3-SKBR3.rT1/4EBP1.jpg]

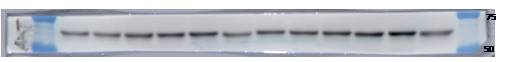

Supplement: Supplementary file 1 [file cancers-13-02778-s001.zip › Figure.S6/Figure5/SKBR3-SKBR3.rT1/AKT MW.jpg]

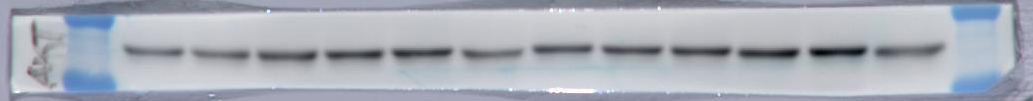

Supplement: Supplementary file 1 [file cancers-13-02778-s001.zip › Figure.S6/Figure5/SKBR3-SKBR3.rT1/AKT.jpg]

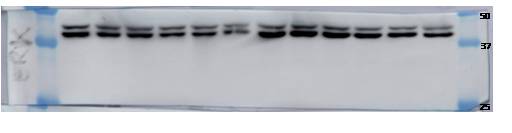

Supplement: Supplementary file 1 [file cancers-13-02778-s001.zip › Figure.S6/Figure5/SKBR3-SKBR3.rT1/ERK MW.jpg]

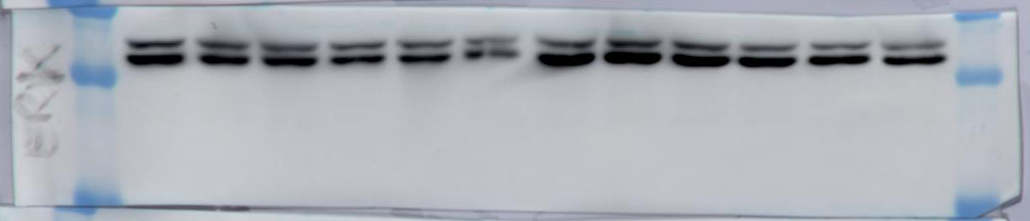

Supplement: Supplementary file 1 [file cancers-13-02778-s001.zip › Figure.S6/Figure5/SKBR3-SKBR3.rT1/ERK.jpg]

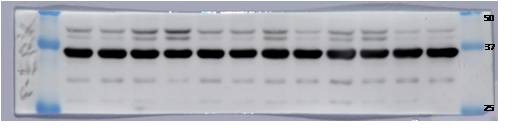

Supplement: Supplementary file 1 [file cancers-13-02778-s001.zip › Figure.S6/Figure5/SKBR3-SKBR3.rT1/GAPDH MW.jpg]

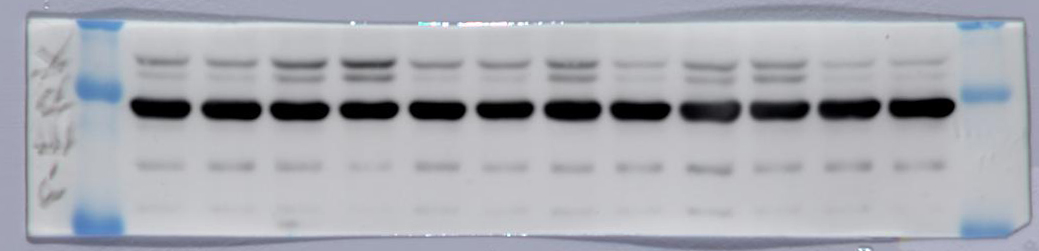

Supplement: Supplementary file 1 [file cancers-13-02778-s001.zip › Figure.S6/Figure5/SKBR3-SKBR3.rT1/GAPDH.jpg]

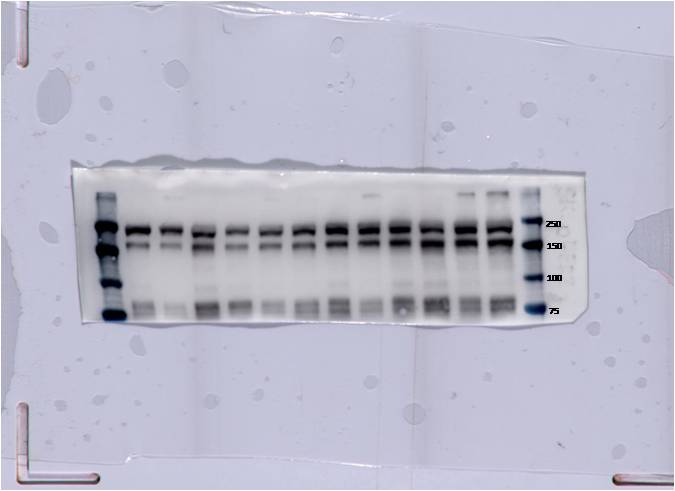

Supplement: Supplementary file 1 [file cancers-13-02778-s001.zip › Figure.S6/Figure5/SKBR3-SKBR3.rT1/HER2 MW.jpg]

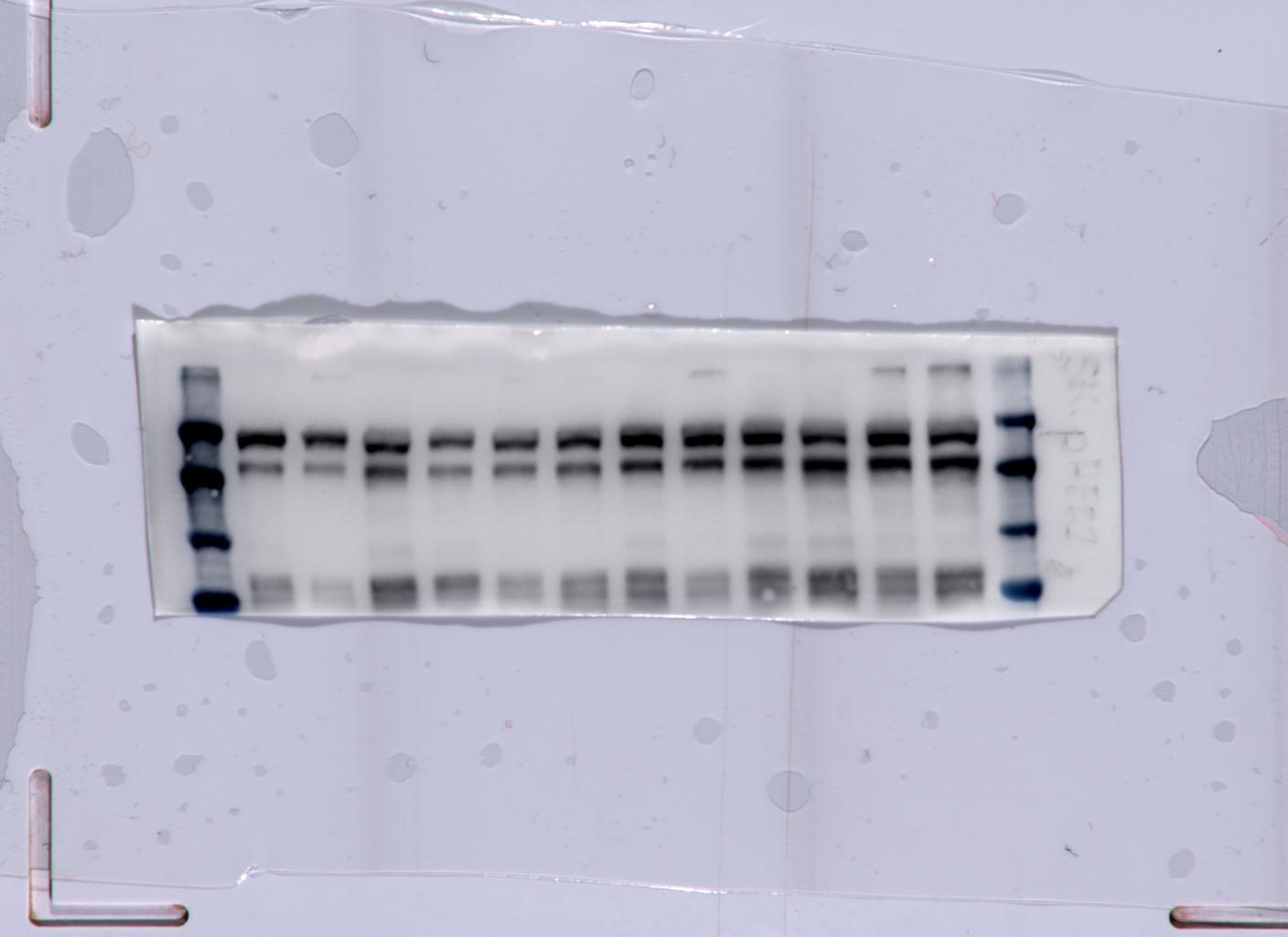

Supplement: Supplementary file 1 [file cancers-13-02778-s001.zip › Figure.S6/Figure5/SKBR3-SKBR3.rT1/HER2.jpg]

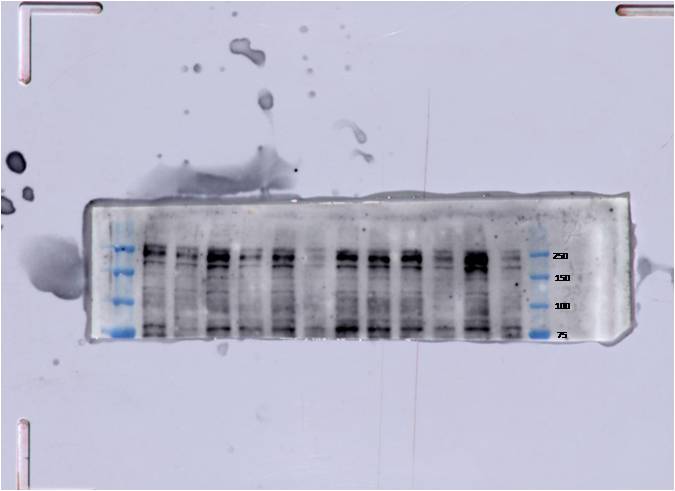

Supplement: Supplementary file 1 [file cancers-13-02778-s001.zip › Figure.S6/Figure5/SKBR3-SKBR3.rT1/HER3 MW.jpg]

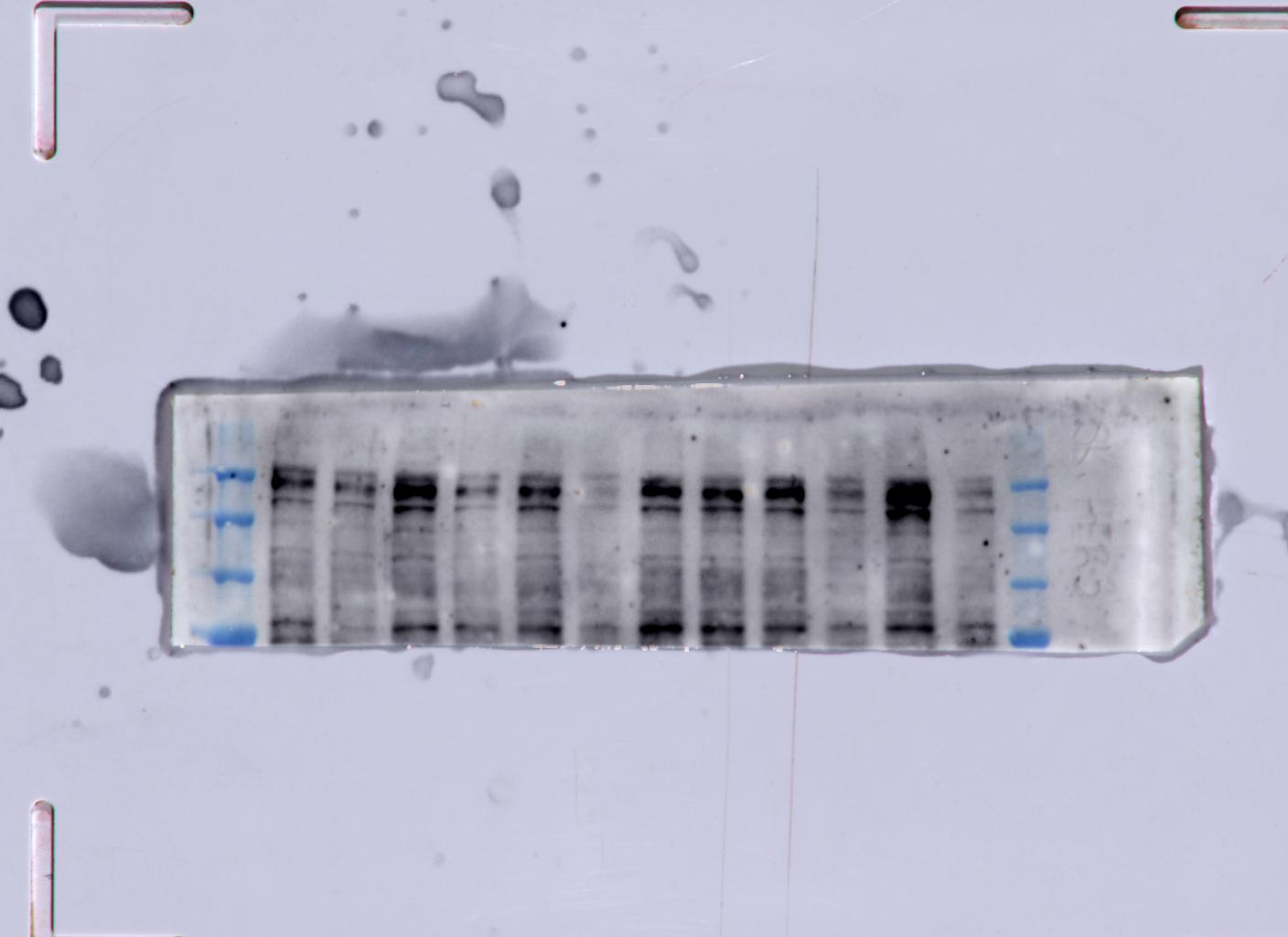

Supplement: Supplementary file 1 [file cancers-13-02778-s001.zip › Figure.S6/Figure5/SKBR3-SKBR3.rT1/HER3.jpg]

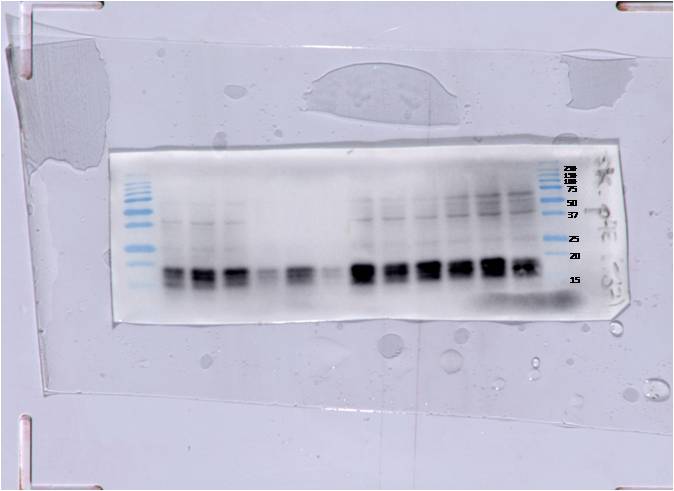

Supplement: Supplementary file 1 [file cancers-13-02778-s001.zip › Figure.S6/Figure5/SKBR3-SKBR3.rT1/p4EBP1 T37 46 MW.jpg]

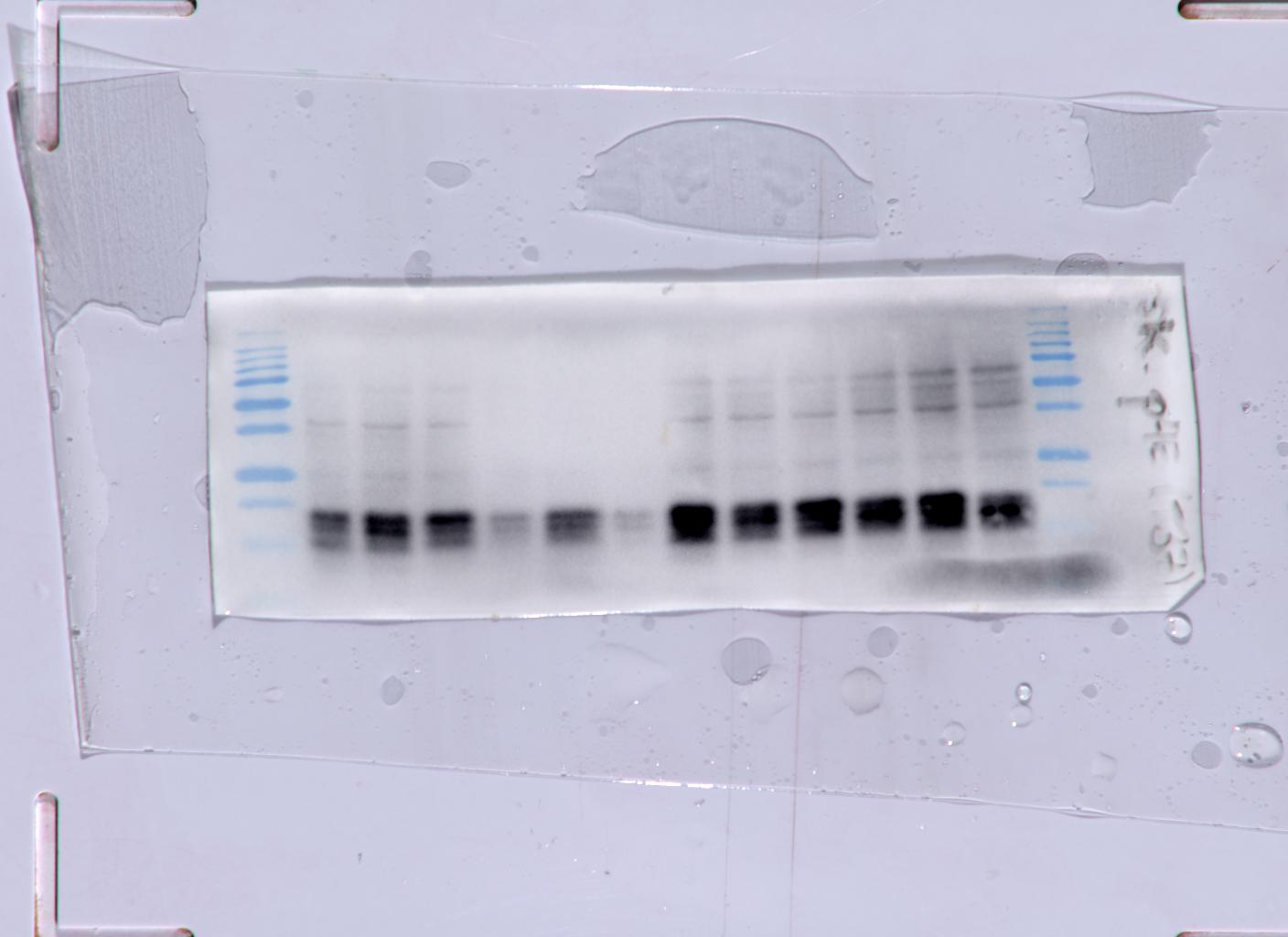

Supplement: Supplementary file 1 [file cancers-13-02778-s001.zip › Figure.S6/Figure5/SKBR3-SKBR3.rT1/p4EBP1 T37 46.jpg]

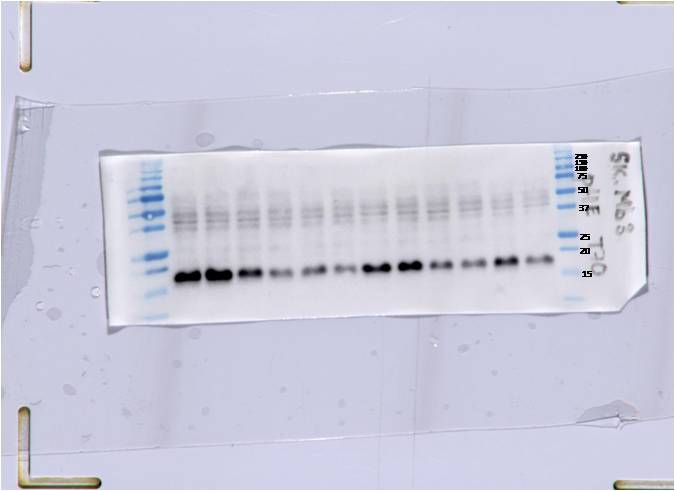

Supplement: Supplementary file 1 [file cancers-13-02778-s001.zip › Figure.S6/Figure5/SKBR3-SKBR3.rT1/p4EBP1 T70 MW.jpg]

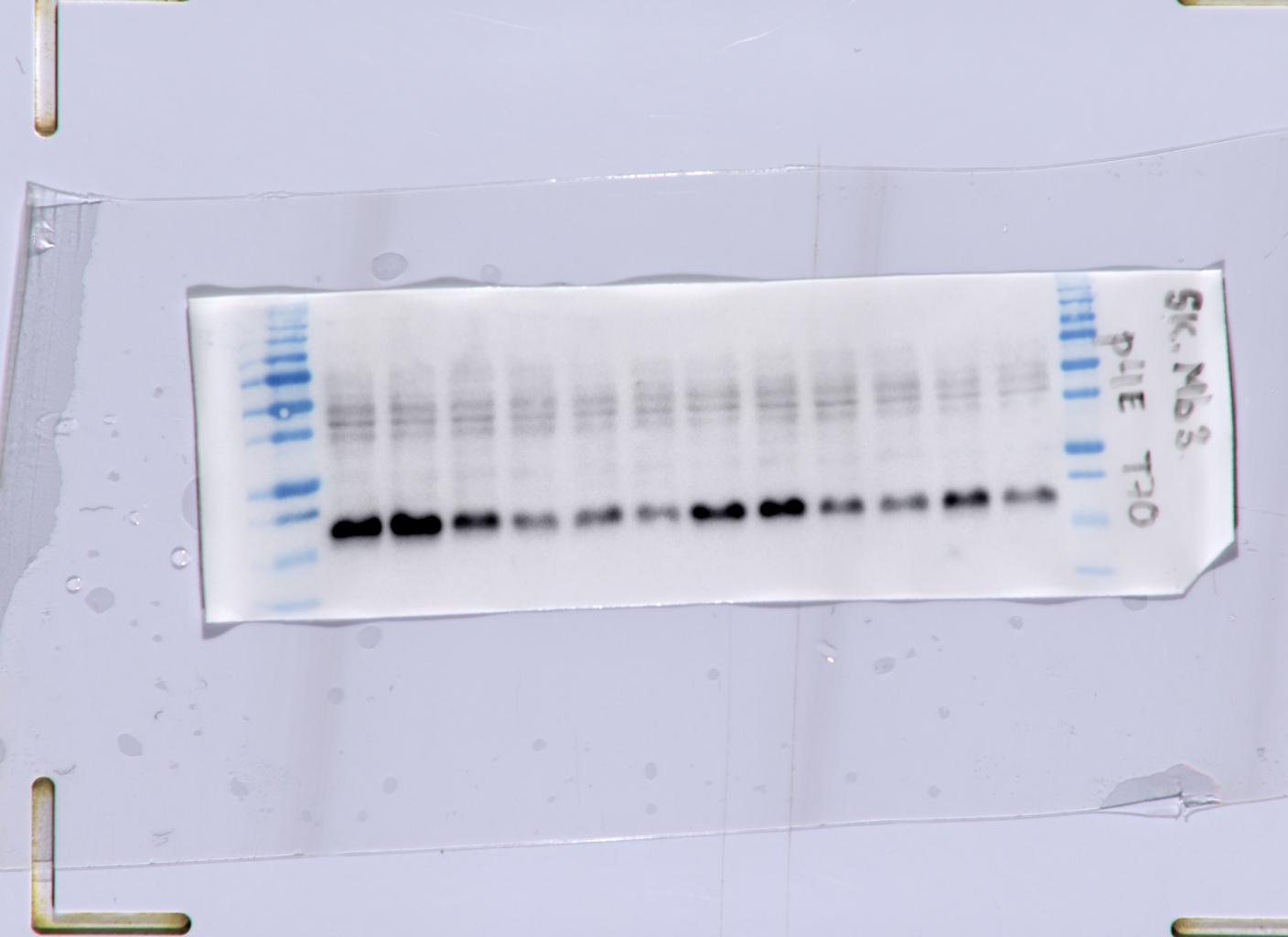

Supplement: Supplementary file 1 [file cancers-13-02778-s001.zip › Figure.S6/Figure5/SKBR3-SKBR3.rT1/p4EBP1 T70.jpg]
